# A vast space of compact strategies for effective decisions

**DOI:** 10.1126/sciadv.adj4064

**Published:** 2024-06-21

**Authors:** Tzuhsuan Ma, Ann M. Hermundstad

**Affiliations:** Janelia Research Campus, Howard Hughes Medical Institute, Ashburn, VA, USA.

## Abstract

Inference-based decision-making, which underlies a broad range of behavioral tasks, is typically studied using a small number of handcrafted models. We instead enumerate a complete ensemble of strategies that could be used to effectively, but not necessarily optimally, solve a dynamic foraging task. Each strategy is expressed as a behavioral “program” that uses a limited number of internal states to specify actions conditioned on past observations. We show that the ensemble of strategies is enormous—comprising a quarter million programs with up to five internal states—but can nevertheless be understood in terms of algorithmic “mutations” that alter the structure of individual programs. We devise embedding algorithms that reveal how mutations away from a Bayesian-like strategy can diversify behavior while preserving performance, and we construct a compositional description to link low-dimensional changes in algorithmic structure with high-dimensional changes in behavior. Together, this work provides an alternative approach for understanding individual variability in behavior across animals and tasks.

## Teaser

The space of strategies for solving a behavioral task can be huge, and many good strategies do not approximate the Bayesian ideal.

## INTRODUCTION

**Fig. 1.**
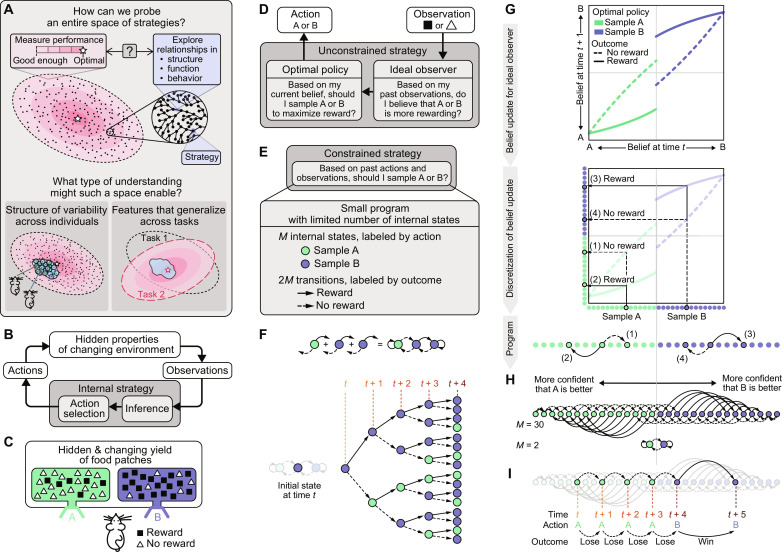
Constructing compact behavioral programs. (**A**) Top: The space of strategies for solving a task can be large, with many strategies that achieve good enough performance. Bottom: Studying relationships between strategies could provide insight into behavioral variability across animals and tasks. (**B**) General task setup: An animal makes inferences about hidden properties of the environment to guide actions. (**C**) Specific task setup: An animal forages from two ports whose reward probabilities change over time. (**D**) The optimal unconstrained strategy consists of an optimal policy coupled to a Bayesian ideal observer. (**E**) We formulate a constrained strategy as a small program that uses a limited number of internal states to select actions based on past actions and observations. (**F**) Each program generates sequences of actions depending on the outcomes of past actions. (**G**) The optimal unconstrained strategy (D) can be translated into a small program by discretizing the belief update implemented by the ideal Bayesian observer and coupled to the optimal behavioral policy. Top: Optimal belief update. Middle: Belief values can be partitioned into discrete states (filled circles) labeled by the action they specify (blue versus green). The belief update specifies transitions between states, depending on whether a reward was received (solid versus dashed arrows). Bottom: States and transitions represented as a Bayesian program. (**H**) Top: A 30-state program approximates the Bayesian update in (G) and has two directions of integration that can be interpreted as increasing confidence about either option. Bottom: The two-state Bayesian program, win-stay, lose-go (WSLG), continues taking the same action upon winning (i.e., receiving a reward) and switches actions upon losing (i.e., not receiving a reward). (**I**) Example behavior produced by the 30-state Bayesian program in (H).

To thrive in an uncertain and changing world, animals benefit from making inferences about hidden properties of the world to guide decisions and plan future actions. This is true of many tasks, such as localizing a food source from noisy measurements of odorants (*1*–*3*), predicting the location of a moving target during pursuit (*4*, *5*), or planning efficient routes through a set of subgoals (*6*, *7*). In these and other domains, there are in principle many possible strategies for making and using inferences to guide behavior and thus many possible ways to achieve good performance. A common theoretical approach is to derive the optimal strategy for maximizing performance on a particular task (*8*–*10*). This strategy can then be used as a benchmark to compare to behavioral data or dissected to understand the algorithmic features that enable optimal performance under different constraints. In the sensory domain, a long history of work has explored optimal coding schemes under bandwidth constraints (*11*–*13*); more recently, several lines of work have explored optimal decision-making strategies under constraints of time (*14*) or computational complexity (*15*). However, by focusing on a single strategy that achieves optimal performance, these approaches do not provide a way to understand the diversity of effective strategies that could be used to achieve ‘good enough’ performance. Humans adopt a variety of heuristic strategies when making decisions, often deviating from the theoretical optimum (*16*). In other animals, studies of decision-making typically characterize average performance, but individuals often exhibit a great deal of variability in behavior within and across tasks (*17*). To what extent this behavioral variability reflects differences in underlying strategy is less clear. A powerful approach to address this question is to study relationships between the many possible strategies for solving any given task. To this end, we develop a framework for building and exploring an entire space of strategies that vary in their computational complexity, behavior, and performance (Fig. 1A, top). Studying relationships in this space could provide insight into why different individuals might adopt different strategies for the same task domain, and how such strategies might generalize across different task domains (Fig. 1A, bottom).

To build a space of strategies, we consider a general scenario in which an animal makes observations about a changing environment and uses those observations to guide future actions that lead to rewards. To increase rewards, the animal can rely on an internal strategy to make inferences about hidden properties of the environment and use those inferences to guide more effective actions (Fig. 1B). This scenario forms the basis of a broad range of tasks; for specificity, we focus on a widely studied task used in humans (*18*, *19*), nonhuman primates (*20*, *21*), rodents (*19*, *22*–*25*), and flies (*26*) in which an animal forages for rewards from two ports whose reward probabilities change dynamically over time (Fig. 1C and Materials and Methods).

In such a scenario, the optimal strategy for maximizing rewar1ds can be derived in two sequential steps, via two complementary approaches (Fig. 1D) (*27*–*29*): (i) optimal inference, for which Bayesian techniques can be used to derive the ideal observer that uses incoming observations (e.g., the presence or absence of reward) to update an internal belief about hidden properties of the world (e.g., the identity of the most rewarding port), and (ii) optimal action selection, for which reinforcement learning techniques can be used to derive the behavioral policy (e.g., the port that should be sampled on any given trial) that maximizes rewards given the observer’s internal belief. Achieving optimal performance requires updating the internal belief with arbitrarily fine precision. Given limited precision to store and update this belief, there are no theoretical guarantees that the best strategy can be derived by separately optimizing an ideal observer and a behavioral policy.

We develop an alternative approach that circumvents this problem and that enables us to directly probe how such limitations affect performance. Instead of optimizing a single strategy, we enumerate all possible strategies that use a limited number of internal states to guide actions based on past observations (Fig. 1, E and F). The number of these internal states constrains the amount of memory that can be used to store information about the outcomes of past actions. In the limit that the number of states becomes infinite, we show that this formulation can exactly reproduce the optimal strategy described above, complete with the interpretation that actions are guided by an evolving internal belief. However, when the number of states is finite and small, we show that this formulation yields a wide diversity of resource-limited strategies that no longer lend themselves to the same interpretation but nevertheless achieve good performance. We refer to these strategies as “small programs,” each of which specifies a different algorithm for guiding actions based on past observations and can thus serve as a generative model of animal behavior.

## RESULTS

We construct the space of programs using the set of actions and outcomes that specify the task itself (Fig. 1E). For the specific dynamic foraging task that we consider, there are two actions that correspond to sampling from each of the two reward ports. For each action, there are two possible outcomes that correspond to the receipt or omission of reward. We use this set of actions and outcomes to specify the elements of our small programs: Each program consists of (i) a finite set of internal states that are labeled by action and (ii) a finite set of transitions between states that are labeled by outcome. For a program with *M* internal states, there are 2*^M^* possible labelings of these states and 2*M* transitions between states that can be arranged in up to *M*^2*M*^ different configurations. Together, the number and labeling of internal states and the configuration of transitions between states specify the “structure” of the program. Different program structures generate different actions depending on the outcomes of past actions; we use these sequences of outcome-dependent actions as a readout of the “behavior” of each program (Fig. 1F).

At one extreme, given an infinitely large number of internal states, the transitions between states can be chosen to exactly reproduce the belief update derived via Bayesian inference (Fig. 1G). In this case, the internal states can be sorted by belief and used to specify the optimal actions conditioned on belief (Fig. 1, H and I; illustrated for *M* = 30); in the limit that *M* → ∞, the resulting program achieves optimal performance. At the other extreme, given only two internal states, the best program achieves much lower, albeit better-than-chance, performance by implementing a “win-stay, lose-go” (WSLG) strategy in which it continues taking the same action upon receiving reward and switches to the alternative action upon an omission of reward (Fig. 1H; *M* = 2). WSLG is the smallest program that can approximate the Bayesian update. We use these two performance extremes to define and bound the space of “good” programs whose performance exceeds that of WSLG. The number of such programs depends on the parameters of the task; for example, in more volatile environments where the reward probabilities change more frequently, both WSLG and the optimal Bayesian strategy exhibit similar performance, and there is little room to improve upon the WSLG strategy (fig. S1). We thus chose task parameters that give rise to a large performance gap between these two strategies (Materials and Methods).

**Fig. 2.**
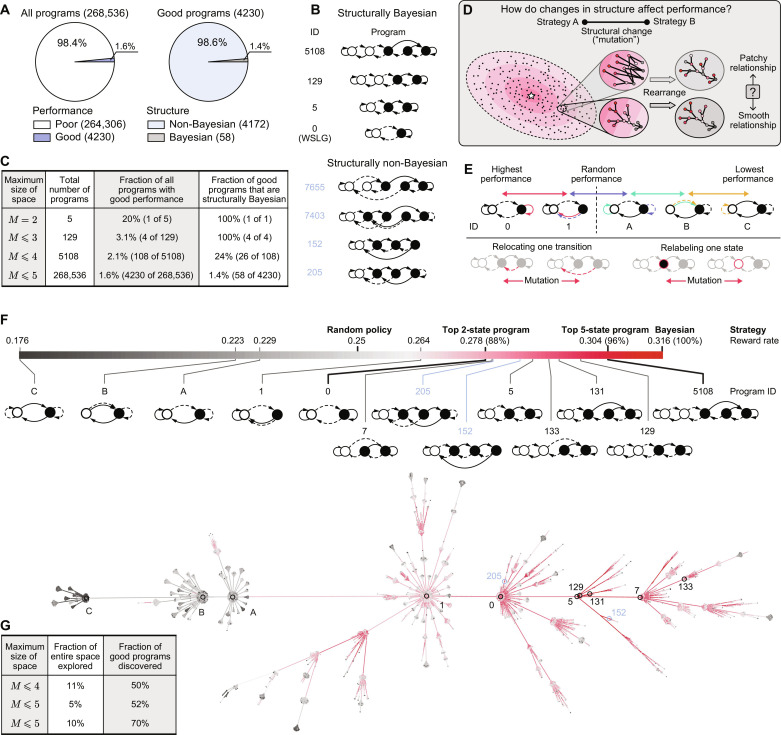
The space of behavioral programs is highly structured. (**A**) There exist 268,536 unique programs with up to *M* = 5 internal states; these can be grouped according to performance (left) and structure (right). Note that the optimal Bayesian strategy, with *M* → ∞, is not included in this ensemble. (**B**) Structurally Bayesian programs have two clear directions of integration that can be interpreted as increasing confidence about a particular option (see Fig. 1H); in contrast, no matter how one orders the program states, structurally non-Bayesian programs do not have such clear integration or interpretation. (**C**) Decomposition of program space for increasingly large programs. As the maximum program size increases, structurally Bayesian programs comprise an exponentially small proportion of all good programs. (**D**) Depending on the underlying organization of the program space, changes in structure could lead to patchier or smoother changes in performance. (**E**) Sorting the set of two-state programs by performance reveals that each neighboring pair of programs is related by a single algorithmic mutation (top; colored arrows), defined as a relabeling of a single state or a relocation of a single transition (bottom). The “win-go, lose-stay” program (program C) suffers from the worst performance; it requires three mutations for this program to exceed chance performance and four mutations to match WSLG (program 0). (**F**) We designed a tree embedding algorithm to extract relationships between program structure and performance, shown for all programs with up to *M* = 4 states (note that program 5108 is not in this embedding; see fig. S5 for a full visualization up to *M* = 5 states). In this embedding, each node corresponds to a single program, each edge corresponds to a single mutation, and colors indicate performance. (**G**) An evolutionary algorithm discovers a large fraction of all good programs by searching a small fraction of the entire program space.

For this set of task parameters, we enumerated and evaluated the performance of all unique programs that have up to five internal states (Materials and Methods). There are 268,536 such programs; of these, 4230 (1.6%) exhibit good performance (i.e., they exceed WSLG; Fig. 2A). Within the subset of good programs, only 58 (1.4%) are “structurally Bayesian” in nature, such that their state transitions can be obtained by approximating the belief space of a Bayesian strategy; the remaining 98.6% of good programs cannot be obtained by approximating a Bayesian strategy (Fig. 2, B and C). Thus, the majority of programs that we discovered through this enumeration are structurally distinct from the class of strategies that we would have devised through handcrafted approaches.

We next examined relationships between the structure and performance of individual programs across the entire program space. To this end, we asked whether small changes in program structure lead to small to changes in performance, as would be indicative of a smooth relationship between structure and performance (Fig. 2D). When we rank-ordered the set of two-state programs by performance, we found that neighboring programs are separated by a single algorithmic “mutation,” defined by relocating one transition or relabeling one state (Fig. 2E; see fig. S2 for an example of a single mutation between two programs of different sizes). We used this observation to design a tree embedding algorithm that captures the minimal relationships necessary to link changes in structure to changes in performance (figs. S3 and S4A). This algorithm iteratively links pairs of programs by assigning a “child” program to the smallest and highest-performing “parent” program within a single mutation. The result of this algorithm can be visualized as a two-dimensional (2D) tree whose nodes correspond to individual programs and whose edges correspond to single mutations between programs. We can then color individual nodes by performance (or any other attribute); if small changes in program structure give rise to consistent changes in performance, then we should then observe a smooth gradation in color across the entire tree. This is indeed what we find (Fig. 2F and fig. S5); this relationship is not as smooth when the embedding is performed with respect to a different attribute other than performance (fig. S6). Within this embedding, nearly all good programs form a single connected subtree; this subtree emerges across a wide range of performance thresholds (fig. S7) and even when we randomize performance prior to performing the embedding (fig. S8). Thus, single mutations to a good program will tend to result in another good program. This relationship was sufficiently strong that we were able to design an evolutionary search algorithm (fig. S9) that efficiently recovered a large majority of good programs by searching a small fraction of the entire program space (Fig. 2G).

**Fig. 3.**
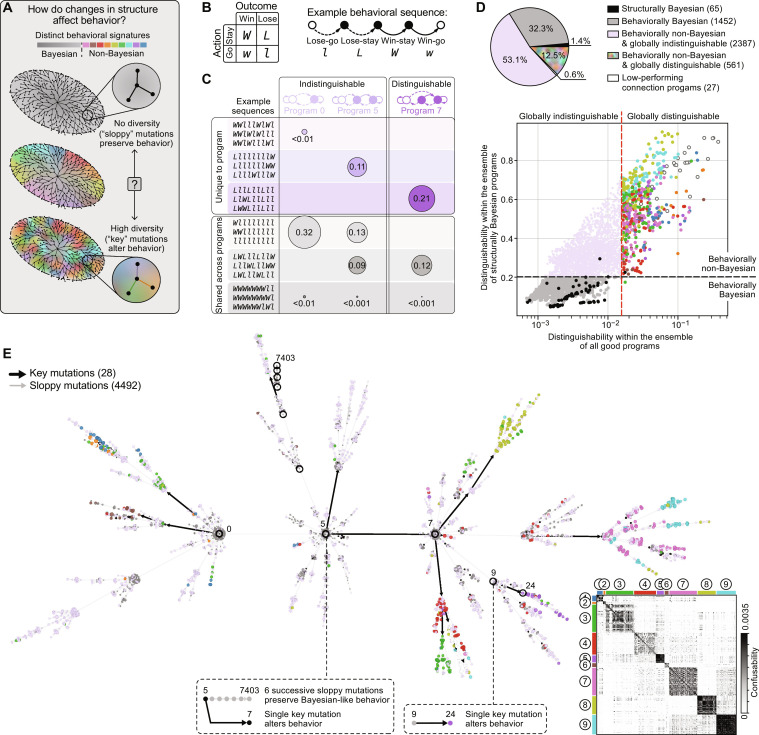
Behaviorally distinct programs emerge from a handful of key mutations. (**A**) Structural mutations could have differing impact on behavior depending on whether they create behavioral diversity. (**B**) Example behavioral sequence expressed in terms of actions (“stay” and repeat an action versus “go” and select a different action) conditioned on past outcomes (“win” versus “lose”). (**C**) Some behavioral sequences are unique to each program (top rows), and some are shared (bottom rows; circle sizes denote the relative probability of observing sequences within each category, computed across all sequences produced by the set of three programs). Program 7 is distinguishable because many of its most probable sequences are not shared. (**D**) We measure how easily each program can be distinguished among the ensembles of good programs and structurally Bayesian programs (*M* ≤ 5), and we use thresholds to select globally distinguishable and behaviorally non-Bayesian programs (colored points; Materials and Methods). We use 262 connection programs to embed good programs in a single connected subtree; 27 of these exhibit lower-than-random performance (low-performing; Materials and Methods). (**E**) A behavioral tree embedding relates program structure and behavior, shown for all programs in (D). As in Fig. 2D, nodes and edges correspond to programs and mutations, respectively. Node sizes denote global distinguishability; colors denote groupings in (D). The confusion matrix inset clusters globally distinguishable programs into nine behavioral subgroups; for visualization, the heatmap saturates at one SD above the mean. By traversing from the root node (WSLG; program 0) toward leaf nodes, a small number of key mutations can generate behavioral diversity, indicated by colored points (e.g., a single key mutation to program 9 alters the behavior of its descendant, program 24). Programs can also undergo sloppy mutations that do not substantially change behavior (e.g., several sloppy mutations to program 5 preserve the behavior of its descendant, program 7403).

These results highlight how altering the structure of individual programs can nevertheless preserve good performance. However, the fact that structurally distinct programs achieve similar performance does not necessarily imply that they use the same behavioral sequences to do so. We thus asked whether we could smoothly relate changes in program structure to the changes in behavior that enable high performance, and whether such structural changes tended to preserve or diversify patterns of behavior across the space of good programs (Fig. 3A).

To assess this, we explored how single mutations alter the distribution of behavioral sequences produced by each good program. These behavioral sequences can be defined in terms of outcome-action pairs; the contingencies “win-stay” (i.e., given that I received a reward, repeat the same action) and “lose-go” (i.e., given that I did not receive a reward, do not repeat the same action) are examples of such outcome-action pairs (Fig. 3B, top). We enumerated the sequences of outcome-action pairs produced by all good programs, up to a maximum sequence length of 10; we then used the steady-state distribution of these sequences as a description of the “behavioral repertoire” of each program (Materials and Methods).

We first isolated programs whose behavioral repertoire was sufficiently distinct so as to make them easily distinguishable from an ensemble of other programs [Fig. 3C and Materials and Methods; see also (*30*)]. We measured this distinguishability with respect to two ensembles of programs: the entire set of good programs and the subset of structurally Bayesian programs. We used the first measurement to select programs whose behavior was sufficiently unique so as to make them globally distinguishable within the ensemble of good programs; we used the second measurement to select programs whose behavior was sufficiently non-Bayesian so as to distinguish them within the ensemble of structurally Bayesian programs (Materials and Methods). We found that a majority of programs were not globally distinguishable based on their behavioral repertoire (Fig. 3D; gray and light pink points). However, a large subset (12.5%) of programs was both globally distinguishable and non-Bayesian in behavior (Fig. 3D; brightly colored points).

To understand how this behavioral diversity could arise through mutations in program structure, we designed a second tree embedding algorithm that preferentially assigns a child program to the smallest and most behaviorally similar parent program, again provided that they are within a single mutation of one another (figs. S3 and S4B; to construct a fully connected tree, note that we included a small number of “connection” programs with subthreshold performance; see Materials and Methods for details). This algorithm attempts to create a behaviorally smooth embedding, in which small variations in the structure of a program lead to small variations in behavioral repertoire. Enforcing such smoothness ensures that any behavioral discontinuities arising from single mutations cannot be removed through alternate embeddings; consistent with this, our performance-based embedding that does not impose behavioral similarity results in far more discontinuities (fig. S10). These mutations, which we refer to as “key” mutations, substantially alter the behavioral repertoire of a program; we distinguish these from “sloppy” mutations that largely preserve the behavioral repertoire of a program (Materials and Methods). Figure 3E shows this behavioral tree embedding performed on the ensemble of good programs, with nodes colored according to the classifications shown in Fig. 3D, and with key and sloppy mutations marked in black and gray, respectively. We find that the set of behaviorally Bayesian programs are clustered around the root of the tree; this root corresponds to WSLG, which is Bayesian in both its structure and behavior. In contrast, programs that produce distinct, non-Bayesian behaviors tend to occupy regions near the leaves of the tree, and they typically emerge following a small number of key mutations from the root. These behaviors can be further separated through a simple clustering algorithm (Fig. 3E, inset), which reveals that behaviorally similar subgroups tend to occupy different local regions of the tree. Together, this shows how structural and behavioral diversity across the entire space of programs emerges through an accumulation of mutations to a single root program: Near the root, sloppy mutations introduce structural diversity while preserving Bayesian-like behavior; moving away from the root, key mutations introduce behavioral diversity and endow the program space with non-Bayesian behavior, which is then preserved through subsequent sloppy mutations.

**Fig. 4.**
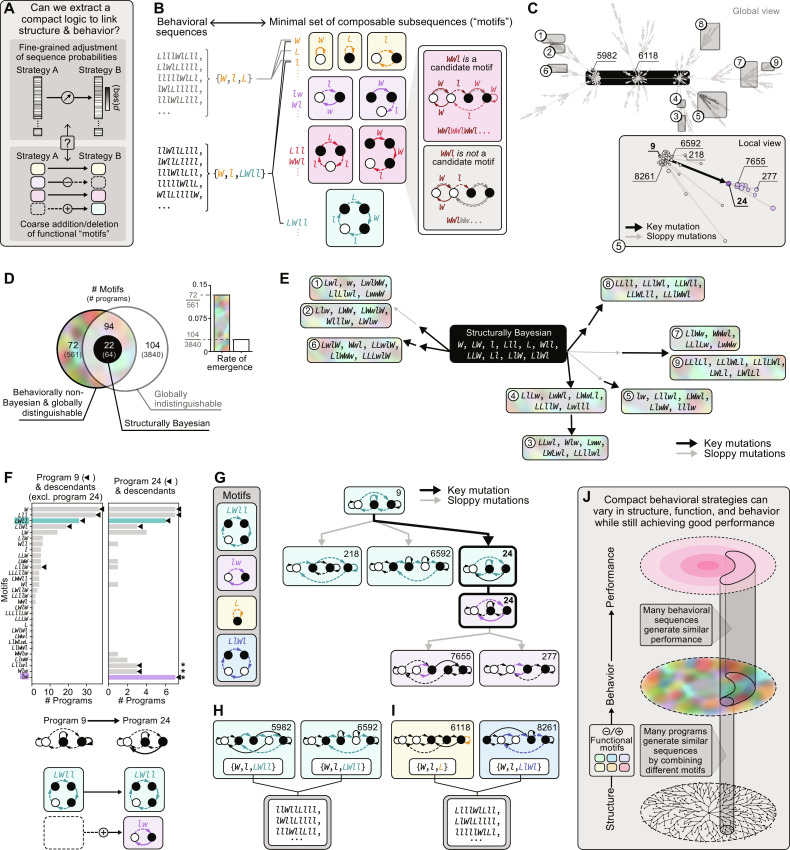
Functional motifs combine to generate diverse behavior. (**A**) We sought an interpretable functional logic to capture the high-dimensional distribution of sequence probabilities (top) using a small number of functional elements (bottom). (**B**) For each program, we extract a minimal set of behavioral subsequences, or functional motifs, that capture a majority of its behavioral sequences (Materials and Methods). Each motif can be visualized by traversing an action-outcome path through a program and must form a stable loop when repeated in succession (red box). (**C**) In (D) to (I), we use motifs to study behavioral features that are inherited along different lineages of the behavioral tree in Fig. 3E. (**D**) Distribution of motifs across different behavioral subgroups. Inset shows the rate at which new non-Bayesian motifs are generated by structurally non-Bayesian programs. (**E**) Top five non-Bayesian motifs shared by a majority of programs within each behavioral subgroup (note that subgroup 7 only expresses four non-Bayesian motifs). See fig. S11 for full characterization. (**F**) A key mutation to program 9 creates new non-Bayesian motifs within program 24 (starred bars; e.g., *lw*) that distinguish the descendants of programs 9 and 24 (left and right histograms, respectively). (**G**) Example program lineage. Motif *LWll* is inherited by the descendants of program 9 through sloppy mutations. Program 24 inherits this motif and creates a new motif, *lw*, via a key mutation; this is inherited by its descendants through sloppy mutations. (**H** and **I**) Two examples of functional convergence between a descendant of program 9 and a program on a distant lineage (C), where structurally distinct programs use the same (H) or different (I) combinations of motifs to generate the same sequences. (**J**) Good performance can be achieved through different behavior, which can be generated by different combinations of functional motifs that are themselves expressed by many structurally distinct programs.

Behavioral sequences provide a holistic description of each program that is directly linked to performance. However, this description requires specifying the probability of observing each of hundreds of thousands of sequences. We thus asked whether we could extract an interpretable functional logic that describes behavior through the addition and subtraction of a compact set of functional elements rather than through changes in a large set of sequence probabilities (Fig. 4A). To this end, we identified a minimal set of subsequences that capture the behavioral repertoire of each program. We refer to the elements in this minimal set as functional “motifs.” Each motif is a specific subsequence that can be visualized by traversing an action-outcome path through a program; when repeated in succession, we require that each motif must be able to generate a stable action-outcome loop (Fig. 4B). An individual program can then be described by the combination of motifs that it uses to generate a majority of its behavioral sequences (Materials and Methods).

We use these motifs to understand the emergence of behavioral diversity across the entire behavioral tree and to study the “heritable” elements of behavior that are passed through successive generations of programs within local branches of the behavioral tree (Fig. 4C). At the root of the tree, the ensemble of structurally Bayesian programs can be described by a compact set of 22 motifs; at the leaves of the tree, we require an additional 166 motifs to capture the behavior of globally distinguishable, behaviorally non-Bayesian programs (Fig. 4D). While a large fraction of these motifs are shared with the ensemble of globally indistinguishable programs, more than one-third of the motifs are specific to the ensemble of behaviorally non-Bayesian programs. These non-Bayesian motifs emerge at a much higher rate compared to the set of globally indistinguishable motifs (inset of Fig. 4D), and they are responsible for the distinct behavioral signatures that differentiate behavioral subgroups (Fig. 4E and fig. S11).

To illustrate how non-Bayesian motifs can arise through mutations in program structure, we focused on a local region of the behavioral tree that is anchored to a single key mutation (Fig. 4C). Figure 4F highlights one program (program 9) before a key mutation; all but one of its descendants produce a similar set of motifs after a sloppy mutation (Fig. 4F, left histogram). One of its descendants, program 24, differs from program 9 by a single key mutation that endows the program with a new set of non-Bayesian motifs (starred bars in the right histogram of Fig. 4F). These new motifs again persist within the descendants of program 24 and are among the motifs that define the behavioral subgroup to which program 24 belongs. Figure 4G illustrates how a single motif is inherited by structurally distinct descendants of each program, and how a single key mutation can introduce a new and heritable motif. The persistence of the same combinations of motifs within multiple structurally distinct programs ensures that these programs generate similar distributions of sequences despite variability in underlying structure (Fig. 4H). However, we also observe that the same distribution of sequences can be generated by different combinations of motifs (Fig. 4I). Both properties give rise to similarity in behavior across distance regions of program space (Fig. 4C).

Together, these results highlight how diversity within an ensemble of effective behavioral strategies arises at multiple different levels (Fig. 4J): through the structural mutations that give rise to different functional motifs; through the combinations of those motifs that give rise to a set of behavioral sequences; and through the different sets of behavioral sequences that underlie good performance.

## DISCUSSION

There are, in principle, many possible ways that the brain can leverage knowledge about an animal’s surroundings to guide behavior (*31*, *32*), depending on resource constraints and performance demands. Here, we used a well-studied dynamic foraging task (*18*–*26*) to illustrate the power of enumerating and studying relationships between a complete ensemble of compact strategies that achieve “good enough” performance. The focus on efficiency and robustness (*33*–*35*), rather than strict optimality, is conceptually similar to earlier artificial intelligence (AI) approaches that explored heuristics for efficiently solving particular tasks (*16*, *36*, *37*); the focus on the enumeration and discovery, rather than the construction, of candidate behavioral strategies bears similarity to the enumeration of structure-function relationships in neural networks (*38*, *39*) and to the deduction of models from animal behavior (*40*, *41*); the focus on a spectrum of good solutions, rather than a single optimum, is conceptually related to work that formalizes the degree of optimality of a system (*42*). By combining these distinct axes, we discovered a vast array of strategies that are structurally and behaviorally distinct from those typically derived from first principles or through the reverse engineering of optimized black-box models.

Our results highlight that the behavioral repertoire for a task can be large, and individual strategies can deviate substantially from the norm without appreciably compromising performance. We characterized these deviations relative to strategies that approximate the Bayesian optimum. Previous studies have identified heuristic strategies that closely approximate this optimum (*43*, *44*); here, we chose to define this optimum using a broad ensemble of strategies whose structure or behavior was indistinguishable from an approximate Bayesian strategy. This broad definition enabled us to identify highly non-Bayesian strategies that could not be explained by approximating the Bayesian optimum. To understand these diverse strategies, we did not attempt to average away variability among them; instead, we chose to study relationships between structure, function, behavior, and performance observed across the entire strategy space. A tree embedding algorithm revealed that this space is largely “smooth” in nature, with iterative mutations in structure often leading to small and consistent changes in behavior and performance. This enabled us to devise an efficient evolutionary search algorithm that could be used to build and study larger strategy spaces for more complex tasks, where full enumeration would be prohibitive. However, this tree embedding also revealed that not all regions of the space are smooth; as a result, we could describe the diversification of strategies in terms of key mutations that alter the behavioral repertoire among different lineages of programs and sloppy mutations that preserve this repertoire within individual lineages.

Together, our findings suggest an alternative methodology for studying individual variability in animal behavior, where rather than summarizing the consistent patterns observed across multiple animals, it is possible to study the entire ensemble of strategies that would be consistent with the behavior of any individual animal. The finding that a myriad of distinct strategies can explain the same behavioral patterns is similar to the concept of sloppiness observed in multiparameter models of other biological systems, where many different parameter combinations can achieve the same model output (*45*–*47*). How these algorithmic strategies might be implemented mechanistically, for example, in recurrent networks of neurons, will likely yield an additional level of sloppiness in which many different mechanistic implementations can generate the same algorithmic strategy. Because we found that structural relationships between strategies contain information about their behavioral differences, we were able to understand how non-Bayesian strategies can emerge through an accumulation of mutations away from a Bayesian strategy, with a small number of key mutations introducing new behavioral diversity and a large number of sloppy mutations preserving that diversity across strategies. This raises an intriguing possibility for studying animal learning in terms of an evolution through program space, where key mutations to an internal strategy could alter the behavioral repertoire of an animal in a manner that is discontinuous in time and appears as abrupt “aha” moments (*48*, *49*).

Moving forward, the approaches adopted here could be used to identify the minimal algorithmic components that enable strategies to generalize across different task domains, something that is not possible when studying single strategies in isolation (*50*, *51*). Observed suboptimalities in animal behavior could reflect a misunderstanding of the task that animals are trying to solve, just as our good but suboptimal strategies might in fact be optimal for tasks not considered here. As a result, deviations from optimality on a single task, which could negatively affect overall performance when accumulated over long timescales, could be offset when balanced across multiple tasks. By constructing and studying relationships in a joint task-strategy space, it might be possible to understand the many sources of robustness that enable different behavioral strategies to remain effective in the face of changing task demands.

## MATERIALS AND METHODS

Here, we briefly summarize key aspects of our methods, and we refer the reader to the Supplementary Materials for further details. In the Supplementary Text, we provide a discussion of alternative methodologies related to embeddings (section S1), sloppiness (section S2), compositionality (section S3), and efficient search (section S4). We also provide a comprehensive discussion of our specific methodologies (sections S5 to S10); we briefly describe these methodologies below, and we refer the reader to the associated Supplementary Materials sections for further details. Last, a description of the code base can be found in Supplementary Text, section S11.

### Behavioral task

We considered a dynamic foraging task (also called a nonstationary two-armed bandit task) in which an animal samples from two different ports to gather rewards (fig. S1A). On each time step, the animal selects one of two binary actions, *a* ∈ {*a*_−_, *a*_+_} (corresponding to sampling a left and right port, respectively) and can receive one of two binary outcomes, *o* ∈ {*o*_−_, *o*_+_} (unrewarding and rewarding, respectively). A hidden binary world state *s* ∈ {*s*_−_, *s*_+_} determines the probability that a given action will produce a given observation; we assume that *s* switches states at a fixed probability *h* ∈ [0.05, .5] per time step, such that each port yields rewards with a high probability *p*_high_ ≡ *p*(*o*_+_∣*s*_±_, *a*_±_) when *a* and *s* are aligned, and a low reward probability *p*_low_ ≡ *p*(*o*_+_∣*s*_±_, *a*_∓_) when they are misaligned. In other words, when the world is in state *s*_+_, taking the action *a*_+_ will yield reward with higher probability; conversely, when the world is in state *s*_−_, taking the action *a*_−_ will yield reward with higher probability. This task can be fully specified by the baseline reward rate of the two arms $\bar{p}=\left(p_{\text{high}}+p_{\text{low}}\right)/2$ (or, alternatively, the reward gain $\Delta \bar{p}=2\bar{p}-1$ ), the reward contrast between the two arms Δ*p* = *p*_high_ − *p*_low_, and the hazard rate *h*. The dynamics of the task are then governed by the following two parameterized conditional probability distributions

$$\begin{array}{cc} p\left(s_{t}|s_{t-1}\right)=\left(\begin{array}{cc} 1-h & h\\ h & 1-h \end{array}\right) & \text{world dynamics}\\ p\left(o|s,a\right)=\frac{1}{2}\left[1+o\left(sa\Delta p+\Delta \bar{p}\right)\right] & \text{reward delivery} \end{array}$$ (1)

### Ideal Bayesian observer

To choose the best action at any point in time, the optimal strategy involves inferring the hidden world state from the outcomes of past actions. To perform this inference, we construct an ideal Bayesian observer that has knowledge of $\bar{p}$ , Δ*p*, and *h* but does not know the current world state *s_t_* (i.e., it does not know the identity of the more rewarding port). The observer maintains a belief *u_t_* ≡ *p*(*s_t_* = *s*_+_ ∣ …) − *p*(*s_t_* = *s*_−_ ∣ …) about the current world state, where *u_t_* ∈ [−1,1]. Upon selecting an action *a* and observing an outcome *o*, the observer can iteratively update its belief according to the following equation (Fig. 1G and fig. S1B)

$$u_{t+1}=\left(1-2h\right)\cdot \frac{a_{t}o_{t}\Delta p+\left(1+o_{t}\Delta \bar{p}\right)u_{t}}{a_{t}o_{t}\Delta pu_{t}+\left(1+o_{t}\Delta \bar{p}\right)}\equiv U\left(u_{t},a_{t},o_{t}\right)$$ (2)

### Optimal Bayesian strategy

Our task is an example of a partially observable Markov decision process (POMDP). Using Bayesian reinforcement learning (RL), we can factorize this POMDP into the two separate problems of (i) deriving the optimal inference to update a belief about the hidden world state and (ii) finding the optimal behavioral policy (also referred to in the text as optimal action selection) to select actions based on the current belief (*27*–*29*). The former can be done using Bayesian formalism, as discussed above. The latter is equivalent to a standard RL problem with a fully observable MDP defined over belief states. With this equivalence, standard RL algorithms such as value iteration can be used to efficiently find the optimal policy π(*a*∣*u*) that maximizes cumulative reward 〈*o* = *o*_+_〉. To derive the optimal policy, we discretize the belief space into 200 non-overlapping, equally sized bins, and we use value iteration to optimize the value function *v*(*u*, *t*):

$$v\left(u,t+1\right)=\underset{a}{\text{max}}\underset{q\left(u,a\right)}{\underbrace{\underset{u\prime ,o}{\sum ‍}p\left[u\prime ,r\left(o\right)|u,a\right]\left[\frac{r\left(o\right)}{t+1}+\frac{t}{t+1}v\left(u\prime ,t\right)\right]}}$$ (3)

where *r*(*o*) = 1 if *o* = *o*_+_ and 0 otherwise. Note that, here, we modify standard value iteration (*29*) with a built-in running average of reward over an infinite horizon. The optimal policy is then a* = argmax*_a_ q*(*u*, *a*) = sgn *u*. This corresponds to a deterministic greedy policy in which the optimal action is always to sample from the reward port that aligns with the observer’s current belief (i.e., it selects *a* = *a*_+_ if *u_t_* > 0 and *a* = *a*_−_ if *u_t_* < 0).

### Discretizing the optimal Bayesian strategy

To construct discretized Bayesian strategies (referred to in the text as structurally Bayesian programs; Figs. 1, G to I, and 2B), we discretized the belief space into *M* ∈ {2,3,4,5} equal non-overlapping bins spanning *u* ∈[ − α*u*_ub_, α*u*_ub_]. Here, *u*_ub_ is the fixed-point belief value achieved upon continual winning (see section S5.5 for derivation), and α ∈ (0,1] is a parameter used to control the range of discretization. Given a set of discrete bins labeled by states *m* = 0, …, *M* − 1, we derived the transition matrix *p*(*m*′∣*m*, *o*) by finding, for each initial state *m* (corresponding to initial belief *u*), the final state *m*′ that is closest to the updated belief value *u*′ upon taking the optimal action a* = sgn *u* and observing an outcome *o*. Because the transitioned belief value can fall between two neighboring discrete states, we additionally considered transition matrices that include all combinations of nearest and next-nearest state transitions. For each transition matrix *p*(*m*′∣*m*, *o*), we assigned a deterministic action to each state as specified by the optimal policy [i.e., we constructed the discrete-state policy π(*a*∣*m*) from the optimal policy π(*a*∣*u*) = sgn *u*]. This process generates a set of discrete Bayesian programs that we then filter using a set of “rule-out rules” to eliminate invalid programs (discussed in the next section). Note that this approach is guaranteed to generate programs that have ordered state transitions consistent with Bayesian belief integration. For more details regarding the Bayesian formalism described above, see Supplementary Text, section S5.

### Selecting task parameters

All results were generated using the following parameter setting: $\bar{p}=0.3$ , Δ*p* = −0.5, and *h* = 0.05. These parameters were chosen by first examining the behavioral difference between the optimal Bayesian strategy and the smallest and best-performing resource-constrained program, the WSLG program. The behavioral difference between these programs serves as a proxy for the number of small programs that can achieve good performance; in certain parameter regimes (e.g., large hazard rates; fig. S1D), the optimal Bayesian strategy converges to WSLG, and the space of good programs collapses. We thus chose task parameters that generate a large behavioral difference between these two programs. For more details regarding the collapse of the good program space, see Supplementary Text, section S6.

### Enumerating a complete ensemble of unique programs

Our behavioral programs are deterministic Markov chains, which are graphs without any inherent notion of node ordering. However, to enumerate over graphs, one has to impose a labeling over nodes. This, together with other sources of symmetry, leads to multiple repeated programs. We eliminate these repeated programs through the following set of rule-out rules:

1) Remove identical programs under node permutation.

2) Remove reducible programs that contain sinks (a subset of nodes that absorb all occupancy during a random walk) and drains (the subset of remaining nodes).

3) Remove periodic programs whose node occupancy distribution does not converge over time.

4) Remove identical programs under node inversion (this corresponds to exchanging *a*_+_ and *a*_−_, which generates identical behavior under the symmetric task that we consider).

5) Remove programs that contain sets of nodes that, when combined, do not alter the behavior of a program (we refer to these nodes as “merger nodes” and the corresponding programs as “merger programs”).

The first three rules are task independent and relate only to generic features of Markov chains. Rules 2 and 3, which eliminate ill-behaved Markov chains, can be checked using an open python library: QuantEcon (*52*). The last two rules are specific to the task we consider here and eliminate redundant programs that generate identical action-outcome sequences to other programs in the ensemble. Note that rule 5, when applied in reverse, can be used to generate equivalent programs of different sizes (discussed in more detail below).

To optimize the enumeration of unique programs, we apply these rule-out rules in a particular order. We first enumerate the action labels for a fixed node ordering. For programs of size *M* = 2 to *M* = 5, this constrains the unique action labels to the following set: [(−, +), (−, +, +), (−, +, +, +), (−, −, +, +), (−, +, +, +, +), and (−, −, +, +, +)] (note that, using rule 4, we do not include the symmetric set of action labels). For any given program size *M*, we enumerate the *M*^2*M*^ possible ways of assigning outcome-dependent transitions to the set of nodes, and we remove all transition matrices that have individual sinks and drains (rule 2; this step is fast, does not depend on the node labels, and eliminates a majority of invalid programs). We then include node labels and remove those programs that have merger nodes (rule 5). From this reduced set of programs, we remove programs that are repeated under node permutations; this step is slow, because it requires checking a single program against a list of valid programs. Last, we remove programs that generate periodic behavior or that have coupled sinks and drains (rules 2 and 3). We leave this step for last because it is the slowest in this process.

This enumeration reduces the set of 2*^M^* node labelings and *M*^2*M*^ node transitions to a much smaller set of valid programs (see Table 1). We can easily enumerate all programs for *M* ≤ 5; this becomes difficult for *M* ≤ 6 and infeasible for *M* ≤ 7. This highlights the importance of constraining an enumeration either through an evolutionary algorithm (discussed below) or by leveraging additional aspects of task structure. For more details regarding program enumeration, see Supplementary Text, section S7.

**Table 1.** Number of unique labelings versus unique programs.

*M*	# Unique labelings (2*^M^M*^2*M*^)	# Unique programs
2	64	5
3	5832	124
4	1,048,576	4979
5	312,500,000	263,428

### Evaluating program performance

The most common way to evaluate the performance of a Markov chain is to use Monte Carlo simulations, which necessitates long behavioral trajectories for reliable convergence. We circumvent this by propagating an entire belief distribution over time, which ensures orders-of-magnitude faster convergence at the cost of storing a full belief distribution in memory and allows us to account for the probabilistic nature of world-state transitions. Note that the initial program state transiently affects the belief distribution, but this impact dissipates after ∼1/*h* = 20 time steps. Here, we consider the steady-state belief distribution that stabilizes after these initial transients. We refer to this algorithm as belief distribution propagation (BDP).

To compute the steady-state belief distribution for the optimal Bayesian strategy (fig. S1C), we first initialize the distribution of belief values using a uniform distribution across *u* ∈ [−1,1], discretized into 200 bins. We then propagate the probability of each belief value upon taking each action and receiving each outcome (i.e., we propagate across all four action-outcome pairs); this then generates four new belief values at *t* = 1 with corresponding probabilities. For a given belief value indexed by *i*, the updated belief value at time *t* + 1 is found by summing across all actions, outcomes, and previous belief values that could have led to the given belief

$$\begin{array}{c} p\left[u_{t+1}^{\left(i\right)},s_{t+1}^{\left(j\right)}\right]=\sum_{i^{\prime },j^{\prime }}‍p\left[u_{t}^{\left(i^{\prime }\right)},s_{t}^{\left(j^{\prime }\right)}\right]\underset{\text{world dynamics}\left(\text{Eq}.1\right)}{\underbrace{p\left[s_{t+1}^{\left(j\right)}|s_{t}^{\left(j^{\prime }\right)}\right]}}\sum_{k,l}‍\underset{\text{reward delivery}\left(\text{Eq}.1\right)}{\underbrace{p\left[o_{t}^{\left(k\right)}|s_{t}^{\left(j^{\prime }\right)},a_{t}^{\left(k\right)}\right]}}\\ \underset{\text{Bayesian inference}\left(\text{Eq}.2\right)}{\underbrace{\delta \left\{u_{t+1}^{\left(i\right)}-U\left[u_{t}^{\left(i^{\prime }\right)},a_{t}^{\left(k\right)},o_{t}^{\left(l\right)}\right]\right\}}}\underset{\text{optimal policy}\left(\text{Eq}.3\right)}{\underbrace{\pi \left[a_{t}^{\left(j\right)}|u_{t}^{\left(i^{\prime }\right)}\right]}} \end{array}$$ (4)

This update takes an analogous form for small programs, with the program state *m* exchanged with the belief state *u* in Eq. 4. Given the steady-state belief distribution *p*(*u*, *s*) [or analogously, *p*(*m*, *s*)], it is straightforward to compute the corresponding steady-state reward rate

$$\langle R\rangle =\sum_{u,s,a}‍p\left(o_{+}|s,a\right)\pi \left(a|u\right)p\left(u,s\right)$$ (5)

We defined good performance as having a steady-state reward rate that exceeded that of the WSLG program, and we referred to set of programs that meet this criterion as “good programs.” We used sequence distribution propagation (an extended version of BDP) to compute the steady-state probabilities of observing different behavioral sequences. As with BDP, we consider the steady-state distribution of behavioral sequences that stabilizes after initial transients. We computed this distribution for sequences of length *l*_max_ = 10, which corresponds to 4^10^ = 1,048,576 distinct sequences. Note that the belief distribution derived through our BDP algorithm is equivalent to computing the leading eigenvector of the transition matrix *T* with joint distribution *p*(*s*, *m*, *a*, *o*). While the eigenvector approach is faster than BDP, it is more difficult to scale up to the larger joint distributions that we encounter when computing sequence probabilities. For more details regarding program evaluation, see Supplementary Text, section S8.

### Mutating between programs

When two programs are of the same size, we define the structural distance between them as the minimal number of algorithmic operations, or “mutations,” that are needed to permute one program into the other, assuming symmetry to the inversion of actions. Here, we define a single mutation as either the reassignment of one transition or the relabeling of one node. When two programs differ in size, we first compute all merger programs (discussed above) that are behaviorally equivalent to the smaller program but are identical in size to the larger program, and then we identify the merger program with the smallest structural distance to the larger program (fig. S2).

### Tree embedding algorithm

To capture relationships between the structure and performance of behavioral programs, we designed a tree embedding algorithm (fig. S4A). Given an ensemble of small programs, we began by first sorting programs according to size (small to large) and then sorting according to performance (high to low). This sorting places WSLG at the top of the program list; this program defines the root of the tree. We then proceeded through the ordered list of programs, assigning each successive child program *i* in the list to a single parent program that is within a single mutation. If there are multiple candidate programs within a single mutation, we select the smallest and highest-performing program to be the parent of program *i*.

When we applied this tree embedding algorithm to the entire ensemble of small programs with *M* ≤ 5 (Fig. 2F and figs. S5 and S8), we found that nearly all good programs were closely connected to form a single subtree, with a small number of disconnected programs. To construct a single connected subtree of good programs, we included an additional 262 connection programs whose performance was below the threshold performance of WSLG. This set of 4492 programs comprise what we call the “good program network” (GPN) (Fig. 3 considers the behavioral properties of this network).

We visualized this tree using Gephi (*53*), with the Y. Hu layout (*54*). To briefly summarize this layout, all pairs of nodes are assigned a repulsive force, and connected pairs of nodes are assigned an attractive (spring) force. The layout algorithm tries to (i) find a 2D embedding that minimizes the energy from attractive and repulsive force between all pairs of nodes; (ii) partition the 2D space into hierarchical regions, so that the forces from many distant nodes can be quickly computed in a coarse-grained manner; and (iii) adaptively cool the layout using a faster initial rearrangement and a slower final refinement. For the tree-like network structures considered here, this layout will try to find a spatial embedding in which the more densely connected root of the tree is displayed near the center of the layout, and the more sparsely connected leaves of the tree are displayed near the edges of the layout.

### Comparing tree embeddings

To quantify the smoothness of a tree embedding, we computed a variant of the *z*-score, measured with respect to a given embedding attribute *a* observed between parent and child programs

$$z\left(\text{prog}\;i\right)=\frac{a_{\text{prog}\;i}-a_{\text{parent}\left(\text{prog}\;i\right)}}{\text{std}\left(a\right)}$$ (6)

where std(*a*) measures the SD in the attribute across the entire ensemble of programs. Note that the numerator of Eq. 6 depends on the embedding, but the denominator does not. In fig. S6D, we compared the distribution of *z*-scores for two different attributes: the performance and the wiring length of individual programs. Both histograms were computed across the ensemble of programs with *M* ≤ 5.

To compute the wiring length of individual programs, we assigned a distance *D* ∈ {0,1,2,3,4} to each transition in the program, where *D* = 0 denotes a self-loop. This distance depends on the ordering of nodes in the program; we thus identified the node ordering that minimizes the net summed transition distance, measured across all transitions in the program. We refer to this net distance as the wiring length of the program. To demonstrate that the tree embedding algorithm is sensitive to different program attributes, we performed a tree embedding that links a child program to the parent program with minimal wiring length (rather than maximal performance; this is shown in fig. S6).

### Efficient search via an evolutionary algorithm

We designed an evolutionary algorithm for efficiently discovering a space of good programs (fig. S9). This algorithm has three key features that distinguish it from generic evolutionary algorithms (discussed in more detail below): (i) At each generation, we consider all possible mutations from a given set of programs; (ii) we use a flexible performance threshold to retain good mutants; and (iii) we “hibernate” unselected mutants to be reconsidered at a later generation.

This algorithm operates on a reservoir of programs that are each labeled with one of three status labels: (i) “morph” (programs that are ready to be mutated); (ii) “frozen” (programs that have previously been mutated); and (iii) “idle” (programs in hibernation). In a given generation, we first select all programs with the label morph. For each program in this set, we generate all programs that are within a single mutation; for a program of size *M*, this includes all merger programs of size *M* + 1 that are within a single mutation. We then filter these mutants using the rule-out rules described above, remove any mutants that are redundant with existing programs in the reservoir, and set the status of all remaining mutants to idle; the status of their parent programs is set to frozen. We then select the top-performing ∼ log (*N*_idle_) programs with label idle and update their status to morph to be mutated in the next generation. All other idle programs remain in the reservoir to be considered at a later generation. After a fixed number of generation, we count the total number of programs in the reservoir (including morphed, frozen, and idle programs), and we measure the fraction of these whose performance exceeds that of WSLG. Figure 2G reports the fraction of good programs that were discovered after 16 generations, beginning with an initial program reservoir that consisted of programs 0 and 1. For more details regarding the tree embedding and evolutionary search algorithms, see Supplementary Text, section S9.

### Behavioral tree embedding algorithm

To quantify the behavioral similarity between programs, we first define a confusion matrix $C_{\mathrm{ij}}^{\ell }\left(\{P\}\right)\equiv p_{\left\{P\right\}}\left(\text{prog}\;i|\text{prog}\;j\right)$ that specifies how likely it is to mistake program *j* for program *i* within a given ensemble of programs {P}, given the distribution of length-ℓ outcome-action sequences that each program generates

$$\begin{array}{c} C_{\mathrm{ij}}^{\ell }\left(\{P\}\right)\equiv p_{\left\{P\right\}}\left(\text{prog}\;i|\text{prog}\;j\right)=\\ \sum_{{\left(o,a\right)}^{\ell }}‍\frac{p\left[{\left(o,a\right)}^{\ell }|\text{prog}\;i\right]p\left[{\left(o,a\right)}^{l}|\text{prog}\;j\right]}{\sum_{i\prime \in \left\{P\right\}}‍p\left[{\left(o,a\right)}^{\ell }|\text{prog}\;i\prime \right]} \end{array}$$ (7)

where *p*[(*o*, *a*)^ℓ^∣prog *i*] specifies the distribution of outcome-action sequences of length ℓ produced by program *i*. Note that the entries of this matrix are always normalized with respect to a particular ensemble of programs {P}, specified by the normalization factor in the denominator. We use $C_{\mathrm{ij}}^{\ell }\left(\{P\}\right)$ to define the behavioral similarity between all pairs of programs within a given ensemble {P}. For a given program *i*, we identify the top $n_{i}^{\ell }=\text{round}\left[1/C_{ii}^{\ell }\left(\{P\}\right)\right]$ programs that are most easily confused with program *i*. These programs then specify binary entries in a behavioral similarity matrix $B_{\mathrm{ij}}^{\ell }\left(\{P\}\right)$ . We define the total behavioral similarity $B_{\mathrm{ij}}\left(\{P\}\right)=\sum_{\ell =1}^{10}‍B_{\mathrm{ij}}^{\ell }\left(\{P\}\right)\in \left[1,10\right]$ by accounting for all sequences up to a maximum length ℓ = 10.

We used *B_ij_*({P}) to perform a behavioral tree embedding of all programs in the GPN (see fig. S4B for a visual summary of this algorithm). Here, {P} is the ensemble of 4230 good programs and 262 connection programs. Analogous to the tree embedding algorithm, we first constructed a list of program pairs that are separated by a structural distance of 1 (*d_ij_* = 1, *M_i_* ≤ *M_j_*). We then sorted these pairs first according to their behavioral similarity (high to low) and then according to their performance (high to low). We then proceeded through this list by selecting, for each child program *j*, the most behaviorally similar and highest-performing parent program *i* from the list of candidate child-parent pairs. This algorithm successfully finds a smooth embedding in which 4464 program pairs are maximally similar (*B_ij_*({P}) = *B*_max_ = 10) and 28 pairs are dissimilar (*B_ij_*({P}) < *B*_max_). The mutations that link these pairs of programs define the sets of sloppy and key mutations, respectively (Fig. 3E).

### Classifying program behavior

We categorized programs based on their behavior using two different thresholds on the “distinguishability” of individual programs (Fig. 3D). These thresholds were used to describe and visualize the features of the good program space and were not used to perform any tree embeddings or to extract functional motifs.

To define the distinguishability of each program, we used the diagonal entries of the confusion matrix $C_{\mathrm{ii}}^{\ell =10}\left(\{P\}\right)$ (Fig. 3C). To define the set of “globally distinguishable” programs, we measured the distinguishability of each program within the “GPN” (i.e., we defined {P} = GPN to be the ensemble of 4230 good programs and 262 connection programs). We defined the total summed distinguishability of this ensemble to be $C_{\text{GPN}}^{\text{tot}}=\sum_{i}‍C_{\mathrm{ii}}^{\ell =10}\left(\text{GPN}\right)$ ; we then defined a threshold value of distinguishability $C_{\text{GPN}}^{*}$ as

$$\sum_{\left\{i|C_{\mathrm{ii}}^{\ell =10}\left(\text{GPN}\right)<C_{\text{GPN}}^{*}\right\}}‍C_{\mathrm{ii}}^{\ell =10}\left(\text{GPN}\right)=\frac{C_{\text{GPN}}^{\text{tot}}}{2}$$ (8)

where $C_{\mathrm{ii}}^{\ell =10}$ is sorted in descending order. In other words, $C_{\text{GPN}}^{*}$ differentiates two sets of programs that each account for half of the total summed distinguishability across the entire ensemble: a minimal set of globally distinguishable programs [ $C_{\mathrm{ii}}^{\ell =10}\left(\text{GPN}\right)\geq C_{\text{GPN}}^{*}$ ] and a maximal set of “globally indistinguishable” programs [ $C_{\mathrm{ii}}^{\ell =10}\left(\text{GPN}\right)<C_{\text{GPN}}^{*}$ ]. Note that in computing $C_{\text{GPN}}^{\text{tot}}$ and $C_{\text{GPN}}^{*}$ , we excluded the set of 27 “low-performing” connection programs whose performance is lower than random but whose distinguishability is among the highest. This threshold ( $C_{\text{GPN}}^{*}=0.0156$ ) is displayed as the red vertical dashed line in Fig. 3D.

To define the set of “behaviorally Bayesian” programs, we measured the distinguishability of each program within the GPN with respect to the subset of structurally Bayesian (“SB”) programs; i.e., we defined {P} = SB to be the ensemble of 65 structurally Bayesian programs within the GPN. We defined a threshold value of distinguishability $C_{\text{SB}}^{*}$ as the maximum value of $C_{\mathrm{ii}}^{\ell =10}\left(\text{SB}\right)$ observed within the set of structurally Bayesian programs. This threshold selects the set of programs that are no more distinguishable from the ensemble of structurally Bayesian programs than the structurally Bayesian programs were from themselves. In defining this threshold, we excluded two outlier programs; one outlier (program 236) has a much higher value of $C_{\mathrm{ii}}^{\ell =10}\left(\text{GPN}\right)$ than all other structurally Bayesian programs; the second outlier (program 11) has a much higher value of $C_{\mathrm{ii}}^{\ell =10}\left(\text{SB}\right)$ than all other structurally Bayesian programs. We therefore used the remaining 63 structurally Bayesian programs to define $C_{\text{SB}}^{*}$ . All programs that fell below this threshold were labeled behaviorally Bayesian [ $C_{\mathrm{ii}}^{\ell =10}\left(\text{SB}\right)<C_{\text{SB}}^{*}$ ], and the remaining programs were labeled “behaviorally non-Bayesian” [ $C_{\mathrm{ii}}^{\ell =10}\left(\text{SB}\right)\geq C_{\text{SB}}^{*}$ ]. This threshold value ( $C_{\text{SB}}^{*}=0.202$ ) is displayed as the black horizontal dashed line in Fig. 3D. Among the group of programs that are both globally distinguishable and behaviorally non-Bayesian, we further clustered the behavioral sequences of these programs using the Python package community_louvain (Fig. 3E, inset).

### Decomposing behavioral sequences into motifs

We highlighted how a minimal set of functional motifs could be used to understand behavioral diversity among the ensemble of good programs (Fig. 4). We capture this behavioral diversity using the distributions of distinguishable behavioral sequences produced by different programs. Analogous to our definition of program distinguishability, we define sequence distinguishability as

$$C_{\mathrm{ii}}^{\text{seq}\;k}\left(\{P\}\right)=\frac{p{\left(\text{seq}\;k|\text{prog}\;i\right)}^{2}}{\sum_{i\prime \in \left\{P\right\}}‍p\left(\text{seq}\;k|\text{prog}\;i\prime \right)}$$ (9)

As with program distinguishability, we define sequence distinguishability with respect to a particular ensemble of programs {P}. To select the subset of distinguishable sequences, we rank-ordered sequences according to this distinguishability, and we selected the set of top-ranking sequences that together accounted for half of the distinguishability across the ensemble of sequences. We represent each of these sequences in terms of the variables *L* (lose-stay), *W* (win-stay), *l* (lose-go), and *w* (win-go).

We next extracted a minimal set of subsequences that could be combined to generate distinguishable behavioral sequences produced by a given program. We use the term “motif” to refer to each subsequence within this minimal set. To extract a set of motifs, we began by first considering all subsequences of length ℓ < 10. For each subsequence, we used all cyclic permutations of the subsequence to generate length-10 sequences. For example, for a subsequence *Wlw* (with permutations *Wlw*, *lwW*, and *wWl*), the corresponding length-10 sequences are *WlwWlwWlw*, *lwWlwWlwW*, and *wWlwWlwWl*. If all of these sequences exist with nonzero probability within the top 95% of distinguishable sequences for a given program, then the subsequence *Wlw* becomes a candidate motif for that program.

Given an ensemble of candidate motifs for a program, we selected the minimum number of shortest motifs that could account for all elements of a given sequence. For example, consider decomposing a sequence *lLWllwlLl* in terms of the candidate motifs {*W*, *Lll*, *LWll*, *LlWl*, *Lllwl*, *Wlw*, *lw*}. We first sorted these motifs by length and then iteratively checked the additional fraction of the entire sequence that can be explained by each successive motif. With this approach, the sequence *lLWllwlLl* can be generated by four motifs (*W*, *lw*, *Lll*, *and LWll*), which each contributes additional explanatory power beyond the previous motifs. That is, *W* explains one of nine of the sequence (the underlined snippet *lLWllwlLl*), *lw* another three of nine (the underlined snippet *lLWllwlLl*), *Lll* another two of nine (the underlined snippet *lLWllwlLl*, where one of three of this snippet has already been explained by previous motifs, as indicated in gray), and lastly *LWll* another three of nine (three of four of the underlined snippet *lLWllwlLl*). We used this approach to extract the smallest set of motifs that could account for the set of most distinguishable sequences for a given program (i.e., those sequences that together account for half of the total distinguishability of the program). A given combination of motifs can be used to generate multiple distinct behavioral sequences.

### Assigning motifs to behavioral subgroups

In Fig. 4 (D and E), we used motif statistics to dissect non-Bayesian behavior within the ensemble of globally distinguishable programs. The structurally Bayesian programs, of which there are 64 (excluding program 11, which is behaviorally dissimilar to the rest of the ensemble), together express 22 motifs. We refer to these as “Bayesian motifs”; we refer to all other motifs as “non-Bayesian motifs.” These motifs are shared with the other ensembles of programs highlighted in Fig. 3D. We divide these remaining ensembles into two groups: the ensemble of 561 globally distinguishable programs (which expresses a total of 188 motifs) and the remaining ensemble of 3840 globally indistinguishable programs (which expresses a total of 220 motifs). These two ensembles share 116 motifs, including all 22 Bayesian motifs, but they also each express sets of motifs that are unique to each ensemble. The globally distinguishable programs express 72 unique motifs, and the globally indistinguishable programs express 104 unique motifs. We used these to define the rate at which programs generate new motifs, given by the number of motifs that are unique to an ensemble, scaled by the number of programs in that ensemble. The results are reported in Fig. 4D.

Given the set of unique motifs within the ensemble of globally distinguishable programs, we assigned each motif *i* to a behavioral subgroup *k* based on the fraction of programs within the subgroup that express the motif [see Fig. 3 (D and E) for behavioral subgroups]. We refer to this as the prevalence of a motif *i* in a group *k*

$$\begin{array}{c} \text{prevalence}\left(\text{motif}\;i,\text{group}\;k\right)\equiv \\ \frac{\text{number of programs in cluster}k\;\text{that express motif}\;i}{\text{number of programs in cluster}k} \end{array}$$ (10)

We then assigned each motif to the behavioral subgroup with the highest prevalence

$$\text{group}\left(\text{motif}\;i\right)\equiv \underset{\text{group}\;k}{\text{argmax}}\left[\text{prevalence}\left(\text{motif}\;i,\text{group}\;k\right)\right]$$ (11)

In Fig. 4E and fig. S11, we used this procedure to assign the 166 non-Bayesian motifs produced by the ensemble of globally distinguishable programs to each of the nine behavioral subgroups shown in Fig. 3 (D and E). In Fig. 4E, we displayed the top five motifs with the highest prevalence within each subgroup. This fraction is displayed for all motifs in fig. S11B and used to specify the size of markers in fig. S11C.

To compute the specificity of a motif *i*, we normalized the prevalence in Eq. 10 to compute the categorical probability of each motif across all behavioral subgroups, *p*_motif *i*, group *k*_ = prevalence(*i*, *k*)/∑*_k_* ‍ prevalence(*i*, *k*). We then used the categorical entropy of this probability to define the specificity of each motif

$$\begin{array}{cc} \text{specificity}\left(\text{motif}\;i\right) & \equiv 1-\text{entropy}\left(p_{i,k}\right)\\ & =1+\sum_{\text{group}\;k}‍\frac{p_{i,k}\text{log}\;p_{i,k}}{\text{log}n_{\text{groups}}} \end{array}$$ (12)

This specificity is shown by the radial distance and opacity of markers in fig. S11C. For more details regarding the behavioral tree embedding algorithm and behavioral classifications, see Supplementary Text, section 10.

## Supplementary Materials

### This PDF file includes:

Supplementary Text Figs. S1 to S19 Tables S1 to S21 References
