# Supplementary material for: A vast space of compact strategies for effective decisions
Source: Sci Adv. 2024 Jun 21;10(25):eadj4064. doi: 10.1126/sciadv.adj4064 (PMC11192086; doi:10.1126/sciadv.adj4064)
Supplement: Supplementary file 1 — Supplementary Text Figs. S1 to S19 Tables S1 to S21 References [file sciadv.adj4064_sm.pdf]

Science Advances
AAAS

Supplementary Materials for

A vast space of compact strategies for effective decisions

Tzuhsuan Ma and Ann M. Hermundstad

Corresponding author: Tzuhsuan Ma, mat@janelia.hhmi.org;
Ann M. Hermundstad, hermundstada@janelia.hhmi.org

Sci. Adv. **10**, eadj4064 (2024)
DOI: 10.1126/sciadv.adj4064

This PDF file includes:

Supplementary Text
Figs. S1 to S19
Tables S1 to S21
References

SUPPLEMENTARY FIGURES

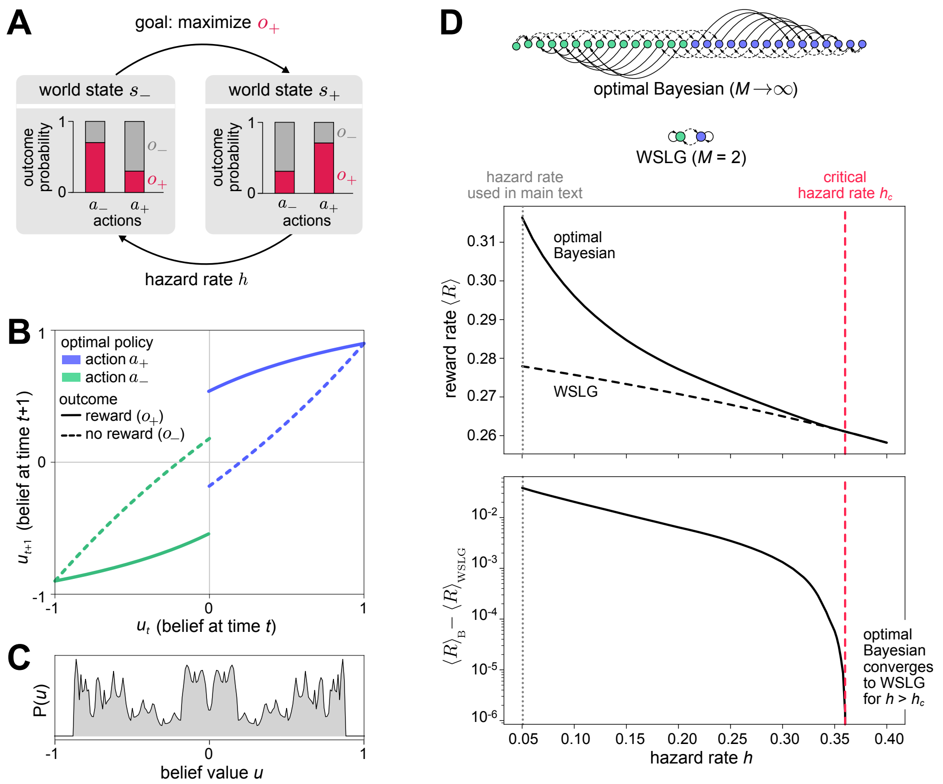

Figure S1: Constructing and bounding the space of good behavioral programs. **(A)** Problem setup. A binary world state switches between two values (s_+ and s_-) with a fixed probability per timestep h. At each timestep, an animal takes one of two actions, a_+ or a_-, and receives one of two outcomes, o_+ (rewarding) or o_- (unrewarding). The current world state determines the probability of each outcome conditioned on each action. We derive the optimal strategy that maximizes average reward. **(B)** Illustration of a single step of the Bayesian belief update under the optimal policy; the belief value is defined as the difference in the posterior belief about each of the two world states—i.e., $u \equiv p(s = s_+ \mid \cdots) - p(s = s_- \mid \cdots)$. This update rule can be qualitatively understood as monotonically increasing/decreasing the belief value u upon winning/losing. **(C)** We obtain the infinite-horizon distribution of belief values (see SI Section 8.1), which can then be used to compute the reward rate in panels E and F. The distribution of belief values is normalized such that it sums to one; for visualization purposes, the vertical scale was chosen to highlight the shape of the distribution. **(D)** The performance gap between the optimal Bayesian strategy and the win-stay, lose-go (WSLG) program decreases with an increasing hazard rate, and drops to zero at a critical hazard rate $h_c = 0.36$ (red dashed line). The large performance gap at lower hazard rates suggests the existence of many good programs that could achieve intermediate performance; we use this observation to select a hazard rate of $h = 0.05$ for evaluating and studying the performance of these programs (gray dotted line). See Methods and Supp. Text Part II, Section 6.5, for more details about the relationship between task parameters and the space of good programs.

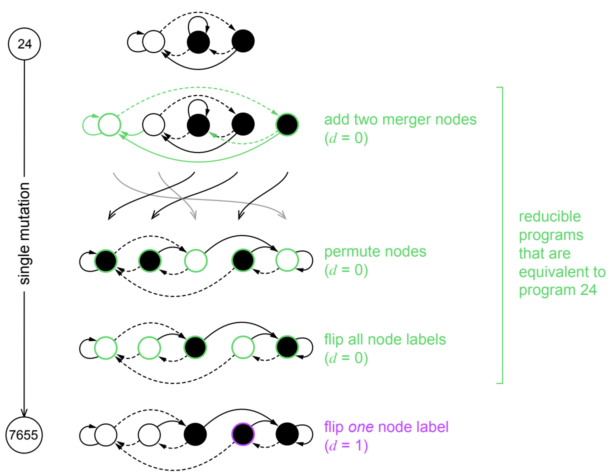

Figure S2: Defining mutations between programs of differing sizes. This example illustrates how two programs of differing size, programs 24 and program 7655, are separated by a single mutation. Before mutating program 24, we first 1) grow the program by adding two redundant "merger" nodes that have identical transitions and action labels as existing nodes in the program, 2) permute the nodes of the program, and 3) flip all action labels within the program, none of which modify the behavior of the program (i.e., these are not considered mutations, and the corresponding programs are separated by structural distances of $d = 0$). In the final step, we flip a single action label, giving rise to the new program 7655. This program is separated from program 24 by a single mutation, and thus a structural distance of $d = 1$. See Methods and Supp. Text Part II, Section 9.1, for more details about computing structural distances between programs.

A

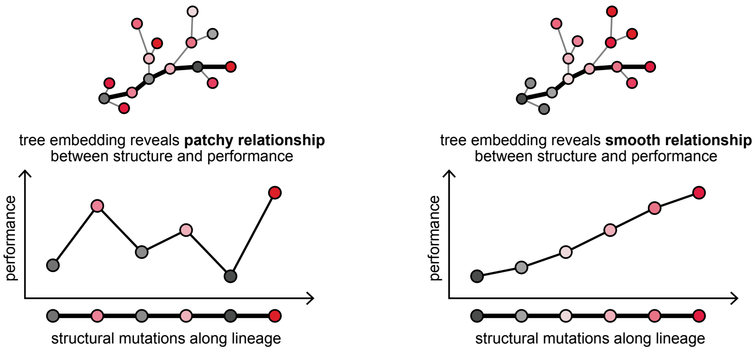

B

type	tree embedding	reduced tree embedding	behavioral tree embedding	evolutionary tree
**figures**	• Fig 2F<br>• SI Fig S6A,C,D	• SI Fig S5<br>• SI Fig S6E<br>• SI Fig S7	• Fig 3E<br>• SI Fig S10A,C,D	• SI Fig S9
**space**	• $M \leq 4$	• $M \leq 5$	• $M \leq 5$; good programs	• $M \leq 5$
**embedding attributes**	• link programs within a single mutation<br>• prioritize high-rank programs, ranked by:<br>(1) size<br>(2) performance<br>(or alternative attribute)	• link programs within a single mutation<br>• prioritize high-rank programs, ranked by:<br>(1) size<br>(2) performance<br>(or alternative attribute)	• link programs within a single mutation<br>• prioritize high-rank programs, ranked by:<br>(1) size<br>(2) behavioral similarity<br>(3) performance	• purely a visualization of the progression of the evolutionary algorithm
**goal of embedding**	• visualize how changes in program structure relate to changes in performance across entire space of programs	• visualize large program spaces, including how the network of good programs is related to the rest of the space	• visualize how changes in program structure relate to changes in behavior within the space of good programs	• visualize successive generations of evolutionary algorithm
**visual readouts**	• smoothness in color gradation across tree	• topological relationship between good programs, connection programs, and all other programs	• clustering of behaviorally-similar programs along different branches of the tree<br>• emergence of new behavioral clusters following multiple key mutations	• number and connectedness of branches
**quantification**	• histogram of z-scores (smaller spread = smoother relationship)	• number of connection programs needed to glue good programs into a single connected tree (fewer connection programs = smoother relationship)<br>• rate of change in number of connection programs with increasing performance threshold	• number of key mutations (fewer key mutations = smoother relationship)<br>• histogram of behavioral similarity (higher values = smoother relationship)	
**variants**	• alternative attribute SI Fig S6B,C,D<br>• randomized reward SI Fig S8	• alternative attribute SI Fig S6F	• unsorted behavioral similarity SI Fig S10B,C,D	
**takeaways**	• the program space is smooth in structure and performance	• the network of good programs is stable (i.e., nearly all good programs form a single connected tree) across a wide range of performance thresholds<br>• the more smoothly that program structure relates to a given feature, the fewer the number of connection programs that are needed to glue programs into a single connected tree	• enforcing behavioral similarity within the embedding:<br>• makes it possible to extract a minimal set of mutations that capture the emergence of behavioral diversity<br>• leads to clustering of similar behaviors along branches of the tree<br>• without enforcing behavioral similarity, mutations between dissimilar programs do not capture behavioral diversity across the tree	• the high efficiency of the evolutionary algorithm (i.e., a small search recovers a large fraction of good programs) further verifies the smooth relationship between structure and performance

Figure S3: Summary of different tree embedding algorithms. (A) Schematics of hypothetical outcomes of the tree embedding algorithm, copied from Fig. 2D. Tracing a particular lineage along the tree (thick black line in upper tree) can reveal abrupt changes or smooth changes in performance (lower plot); we refer to these as "patchy" and "smooth" relationships between structure and performance, respectively. **(B)** Comparison between different tree embedding algorithms used in the main text.

TE algorithm

A

268,533 programs (bottom 3 two-state programs are excluded)

program ID	program size	reward rate
4	2	0.277911
3	2	0.263867
1	2	0.228866
...	...	...
35	3	0.288177
34	3	0.284349
50	3	0.279354
...	...	...
4139	4	0.298633
4138	4	0.294878
650	4	0.293921
...	...	...
134773	5	0.304524
134774	5	0.30245
134793	5	0.30227
...	...	...

program *i* (child)

select a parent program from the top of the list that is within a single mutation of program *i*

first sort programs based on size

then sort based on performance

output

program ID	program size	reward rate	parent ID
0	2	0.277911	1
1	2	0.263867	0
2	2	0.228866	1
3	2	0.223205	2
4	2	0.176363	3
5	3	0.288177	0
6	3	0.284349	0
7	3	0.279354	5
8	3	0.277751	1
9	3	0.274851	7
10	3	0.274725	0
11	3	0.274236	1
12	3	0.272603	0
13	3	0.272248	0
14	3	0.270959	1
15	3	0.270891	0
16	3	0.270099	7
17	3	0.266456	1
18	3	0.266218	1
19	3	0.266163	1
20	3	0.265791	7
...	...	...	...

some parents have many children (e.g., program 0: WSLG)

bTE algorithm

B

32,986 single mutation pairs from the Good Program Network (GPN)

program ID	candidate parent ID	behavioral similarity	reward rate
5108	129	10	0.304529
5108	131	10	0.304529
5109	132	10	0.302449
5109	5108	10	0.302449
5110	129	10	0.302275
...	...	...	...
72	8	1	0.237052
3177	154	1	0.235647
3326	187	1	0.234207
3549	244	1	0.231537
3591	176	1	0.231109

program *i* (child)

beginning at the top of the list, check if program *i* is already committed to a parent program; if not, assign it to the most behaviorally similar and highest performing parent program

first sort program pairs pairs based on behavioral similarity

then sort based on performance

output

program ID	program size	reward rate	behavioral similarity	parent ID
0	2	0.277911	10	0
1	2	0.263876	4	0
5	3	0.288197	10	0
6	3	0.28436	10	0
7	3	0.279354	3	5
8	3	0.277753	10	6
9	3	0.274851	10	7
10	3	0.274725	10	7
11	3	0.274218	10	1
12	3	0.272601	10	0
13	3	0.272275	6	0
14	3	0.270958	7	11
15	3	0.270891	10	9
16	3	0.270099	8	7
17	3	0.266459	10	14
18	3	0.266222	10	14
19	3	0.266191	6	11
20	3	0.265801	10	19
21	3	0.263868	10	11
22	3	0.263647	10	16
...	...	...	...	...

most selected pairs are maximally similar in their behavior (behavioral similarity =10)

28 key mutations (behavioral similarity<10) in the GPN

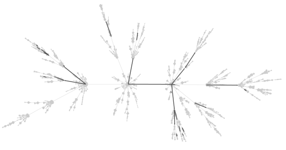

Figure S4: Algorithmic steps of tree Embedding (TE) & behavioral Tree Embedding (bTE) algorithms. (A) Our TE algorithm consists of two primary algorithmic steps: 1) sort a list of programs according to their attributes, and 2) for each child program in the list, find a parent program from the top of the list that is within a single mutation of the child. The tree embedding algorithm shown in Fig. 2D was performed using the single attribute of performance. **(B)** Our bTE algorithm differs from our TE algorithm in that it begins by sorting a list of program pairs that are within a single mutation of one another; each pair consists of one candidate child program and one candidate parent program, defined such that the parent program is no bigger than the child program. Program pairs are sorted first by behavioral similarity, and then by performance. As with the TE algorithm, each child program is assigned a parent program from the top of the list, thereby prioritizing behavioral similarity between parents and children. Inset: the resulting tree shows that the majority of mutations preserve high behavioral similarity. A small fraction (28 out of 4492) of mutations lead to a reduction in behavioral similarity; we define these as "key" mutations. See Methods and Supp. Text Part II, Sections 9.2 and 10.3, for more details about the basic and behavioral tree embedding algorithms, respectively.

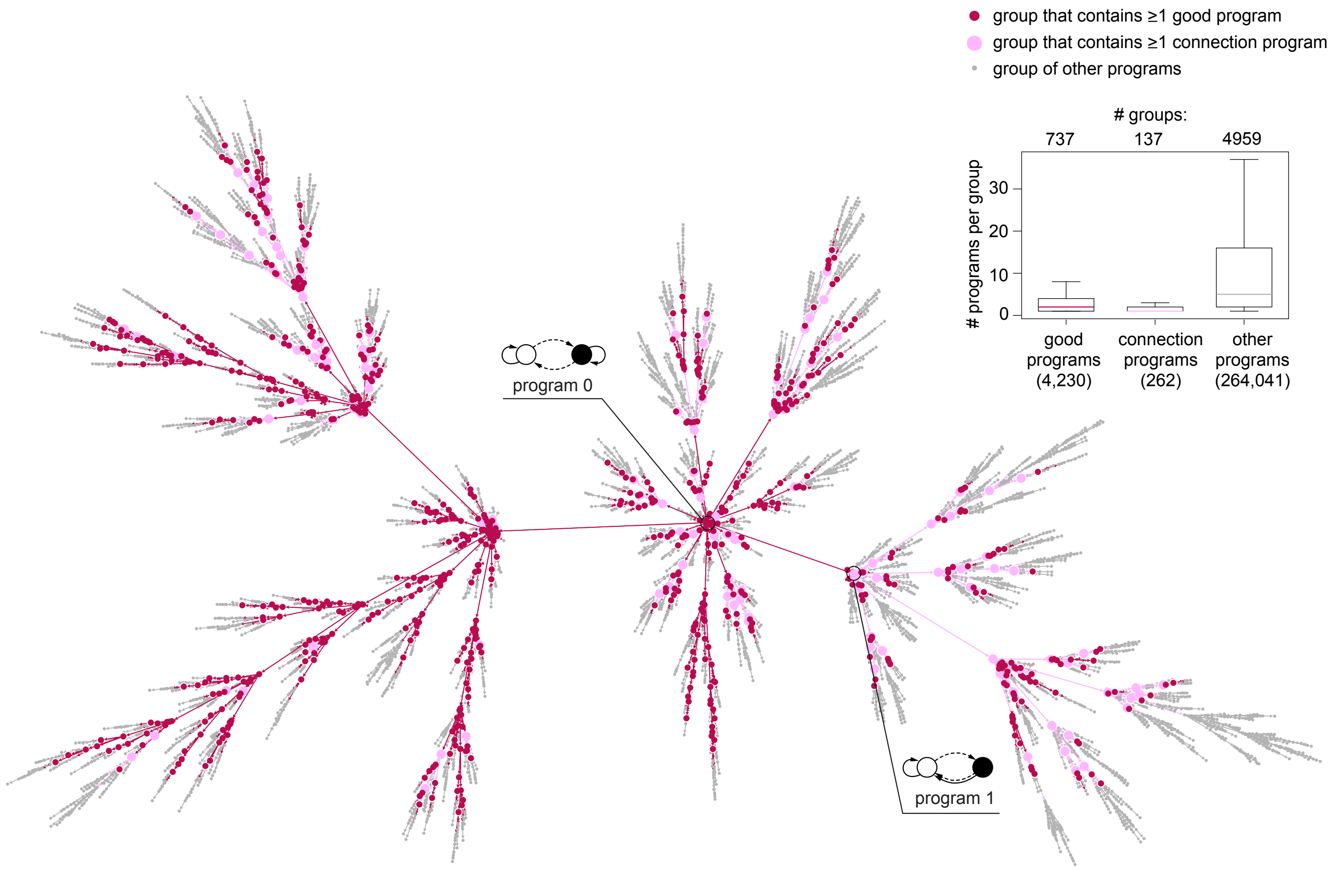

Figure S5: Visualizing the full program space via a reduced Tree Embedding. In the main text, we described a tree embedding algorithm that we used to relate structure and performance across the entire program space. Visualizing such an embedding, with 268,533 programs in total, is challenging; we therefore used a reduced tree embedding to group nodes that are similar in their relational structure on the original tree. Specifically, a group of programs are grouped if both 1) their positions relative to the root node are the same, and 2) their positions relative to the leaf node are the same. Note that we keep the set of connection programs (whose performance is lower than the WSLG program) in order to form a single connected network. In this reduced embedding, each group of programs is colored according to the programs that it contains; groups with one or more good programs are colored in red, and groups with one or more connection programs are colored in pink. Note that this visualization over-represents the proportion of good programs (which account for only 1.7% of the full program space). See Methods and Supp. Text Part II, Section 9.5, for more details about performing the reduced tree embedding.

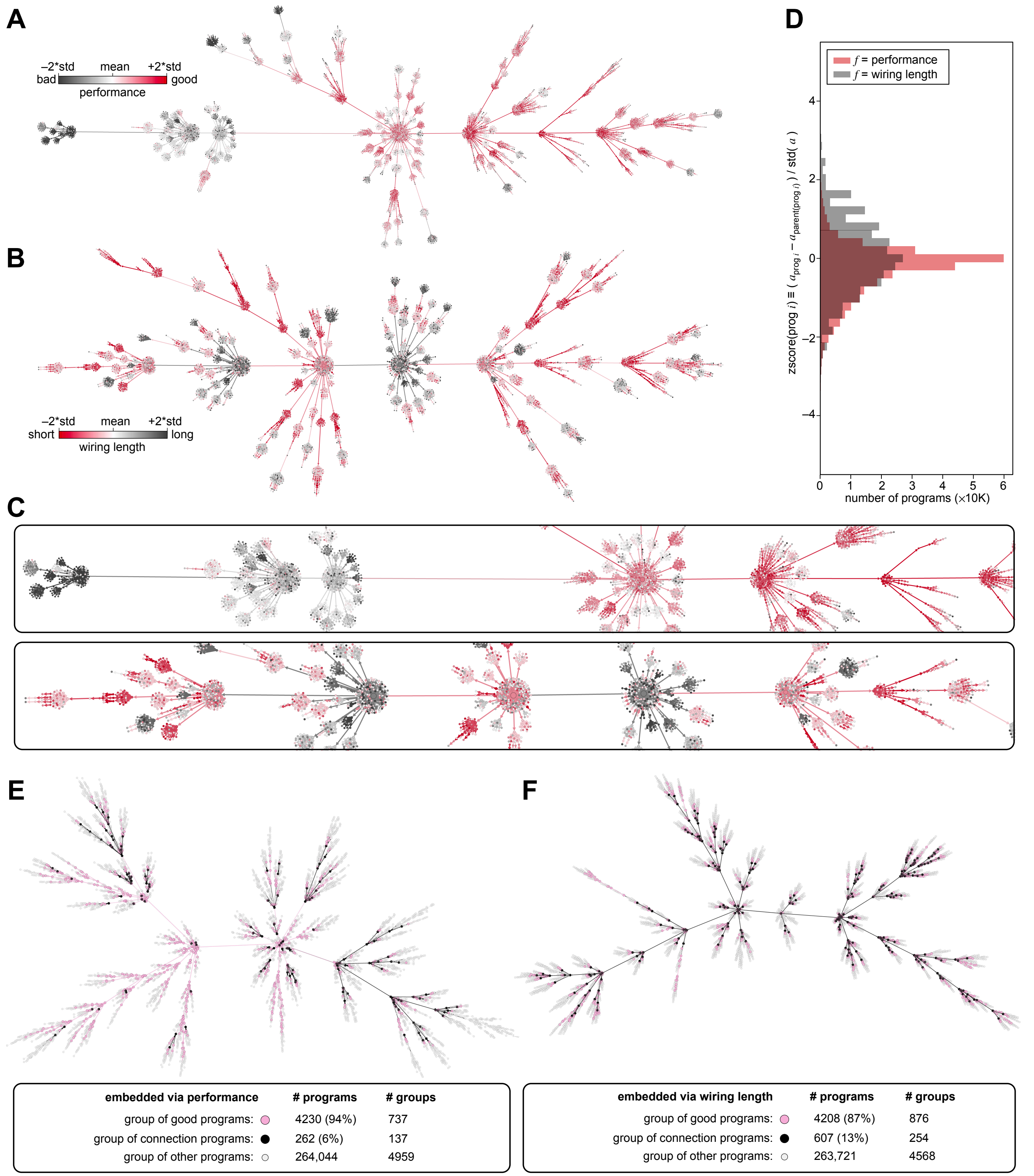

Figure S6: Comparison of tree embeddings performed with different choices of embedding attributes. (A) Tree embedding of the full program space with $M \leq 4$, copied from Fig. 2F and performed using the performance of each program. Note that we adjusted the color bar for a better comparison to the tree shown in panel B; we also used the "no overlap" layout in Gephi in order to highlight the color heterogeneity across the tree. **(B)** Same as panel A, but the tree embedding was performed using an alternative attribute that quantifies the wiring length of each program (Methods). Longer wiring lengths are correlated with lower performance; we thus flipped the color axis in this panel for ease of comparison with panel A. The coloration of the resulting tree embedding is much patchier than the tree embedding in panel A, with series of mutations leading to more variable changes in wiring length (quantified in panel D). **(C)** Zoomed-in view of the tree embeddings performed using performance (upper) and wiring length (lower), highlighting the difference in the color patchiness across the tree. **(D)** Histograms of the difference

Figure S6: (continued from previous page) in a given attribute f measured between the child and parent programs in each tree embedding, normalized by the standard deviation of f. Note that the embedding performed via wiring length leads to a wider distribution, indicating a less smooth embedding with more color patchiness. **(E)** Visualization of the full program space ($M \leq 5$) embedded via performance, copied from SI Fig. S5 and recolored to highlight the number of connection programs required to glue the ensemble of good programs into a single connected network. **(F)** Same as panel E, but embedded via wiring length. For the same approximate number of good programs (~ 4200), the tree embedded via wiring length requires more than twice as many connection programs to form a single connected subtree, in agreement with the increased color patchiness observed in panels B-D.

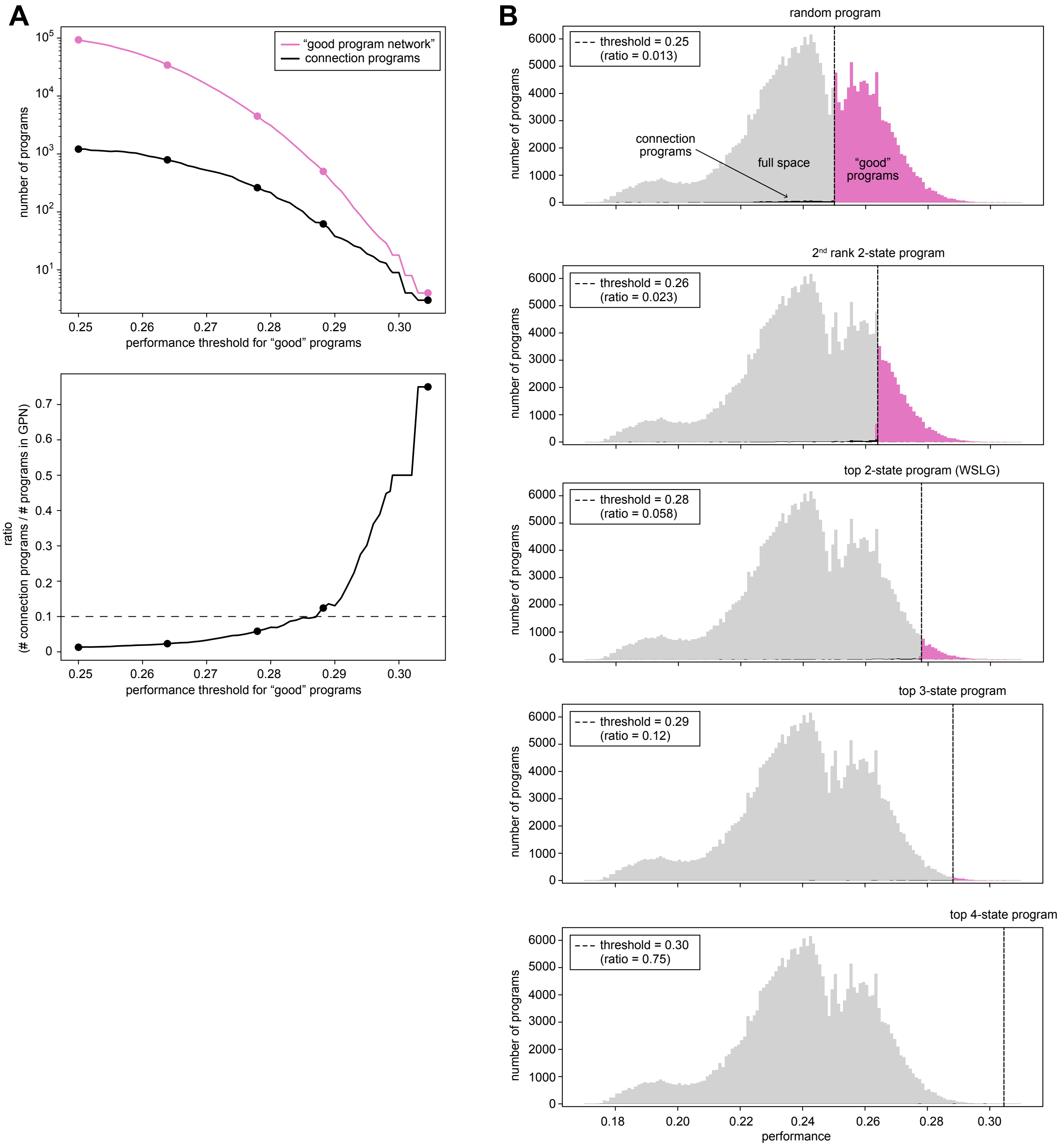

Figure S7: Robustness to different thresholds for "good enough" performance. (A) We vary the performance threshold used to select the ensemble of "good" programs with up to 5 internal states, sweeping between random performance ($\langle R \rangle_{\text{rand}} = 0.25$) and the performance of the best 5-state program ($\langle R \rangle_{\text{max}, M \leq 5} = 0.304$). A given ensemble will require some fraction of "connection programs", whose performance is lower than the performance threshold, to glue the ensemble of good programs into a single connected network (the "good program network", or GPN; Methods). Higher performance thresholds lead to smaller ensembles of good programs (upper panel); these ensembles require a greater fraction of connection programs to form a single connected network (lower panel). However, this fraction remains small across a wide range of performance thresholds. **(B)** Performance histograms for different threshold choices marked in panel A.

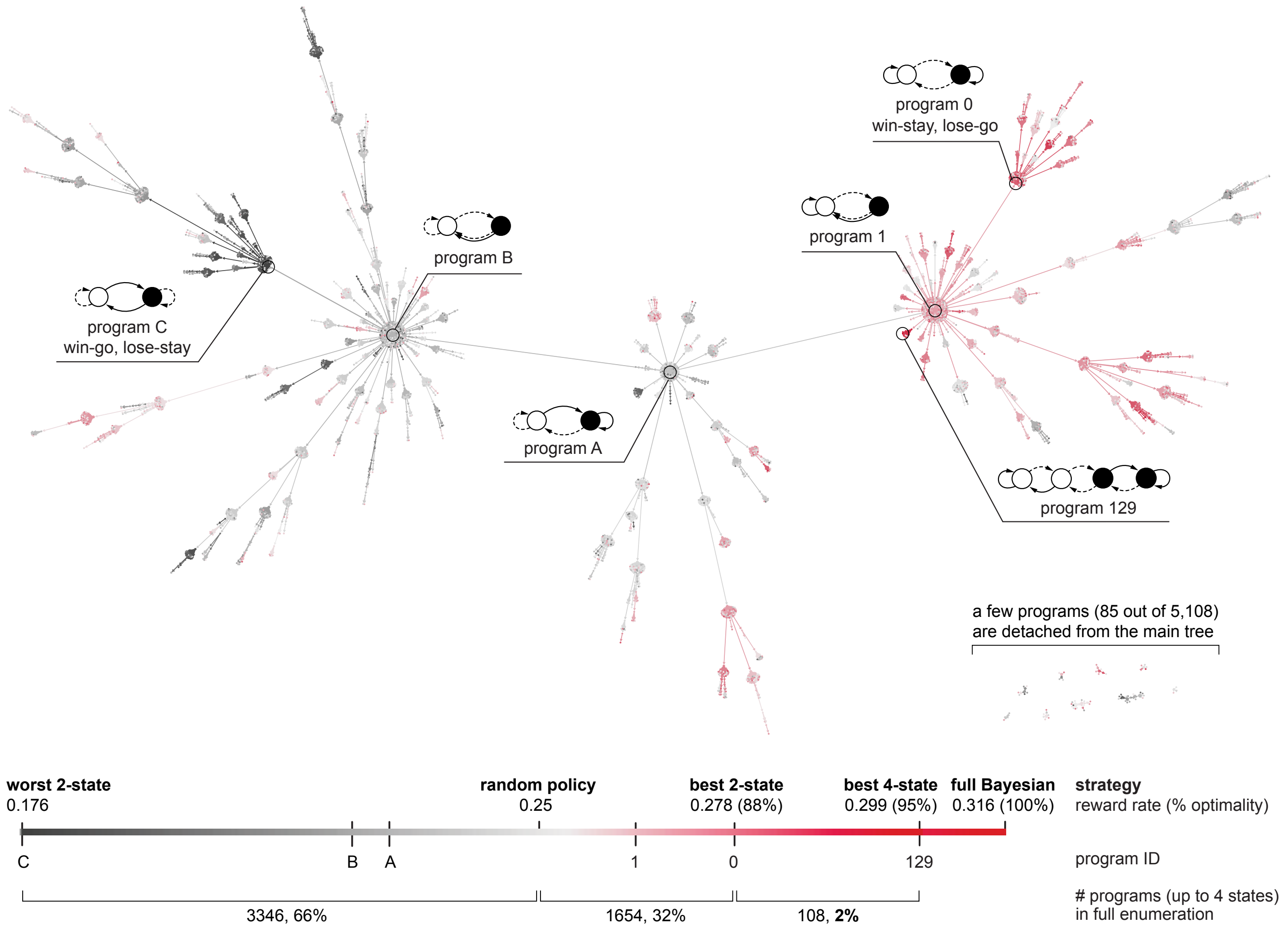

Figure S8: Tree embedding with randomized reward. In contrast to the tree embedding shown in Fig. 2D, here we do not sort programs according to their performance; that is, a child program is no longer biased toward selecting a high-performing parent. The fact that nearly all good programs are still closely connected to form a single subtree suggests that the observed structure in the program space is not an artifact of our embedding algorithm. See Methods and Supp. Text Part II, Section 9.3, for more details about performing this embedding with randomized reward.

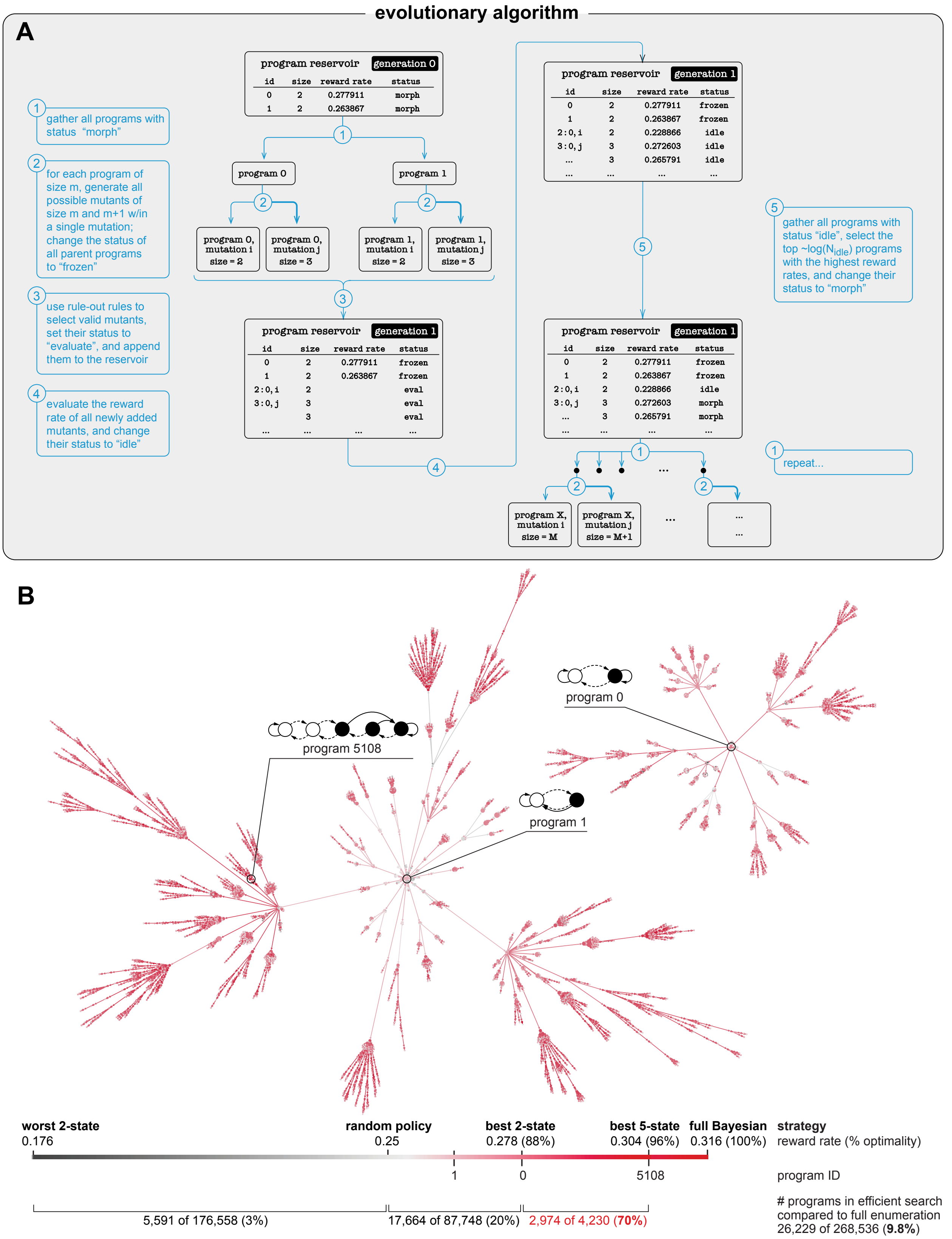

Figure S9: An evolutionary algorithm for efficient search. (A) Algorithmic steps for our evolutionary algorithm. For each generation, a set of new "mutant" programs is validated, evaluated, and selected for the next generation (note that we evaluate all possible mutants without any sub-sampling). The algorithm adopts a flexible selection criterion that allows more that just the top-performing program to generate offspring.

Figure S9: (continued from previous page) (B) Visualization of the ensemble of programs discovered through this algorithm after 16 generations. Note that this ensemble of programs was discovered without consulting any of the previous tree embeddings; the tree shown here is used to visualize the progression of the algorithm, and was not used in the algorithm itself. The algorithm explores only 10% of the full space but discovers 70% of all good programs. Note that the larger tree corresponds to a root program (program 1) with lower performance, indicating that it is beneficial to relax the performance criterion during the search. See Methods and Supp. Text Part II, Section 9.4, for more details about the evolutionary search algorithm.

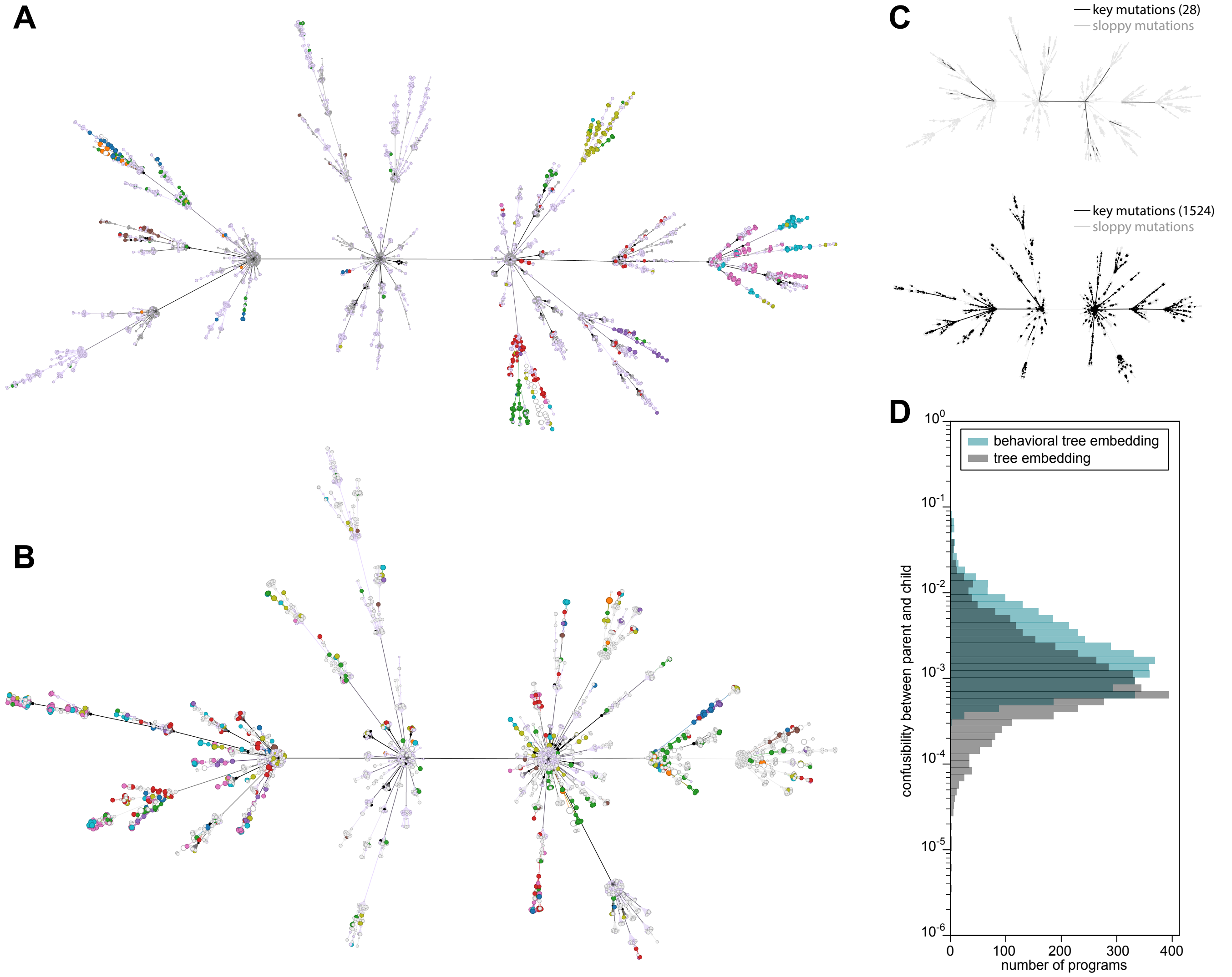

Figure S10: Comparison of behavioral tree embedding performed with or without accounting for behavioral similarity. **(A)** Behavioral tree embedding of the good program space ($M \leq 5$), copied from Fig. 3E and colored according to the behavioral subgroups highlighted in the inset of Fig. 3E. This embedding was performed by assigning child programs to parent programs that are as behaviorally similar as possible (while being within a single mutation of one another). 28 key mutations (thick black arrows in C, upper panel) link programs that have low behavioral similarity, and result in the clustering of behaviorally-similar programs on nearby branches of the tree. **(B)** Same as panel A, but the tree embedding was performed without accounting for the behavioral similarity between programs. The resulting tree exhibits a patchy distribution of programs from different behavioral subgroups, as marked by the patchiness of colors across the tree. **(C)** Embeddings in panels A and B (upper and lower trees, respectively), illustrated to highlight key mutations that link programs with behavioral similarity less than the maximum value of 10 (Methods). The embedding in panel A leads to far fewer key mutations, as expected. **(D)** Histograms of the similarity between child and parent programs in each tree embedding, measured by how confusable they are with one another. Note that the embedding in panel A leads to a greater similarity between child and parent programs, as expected.

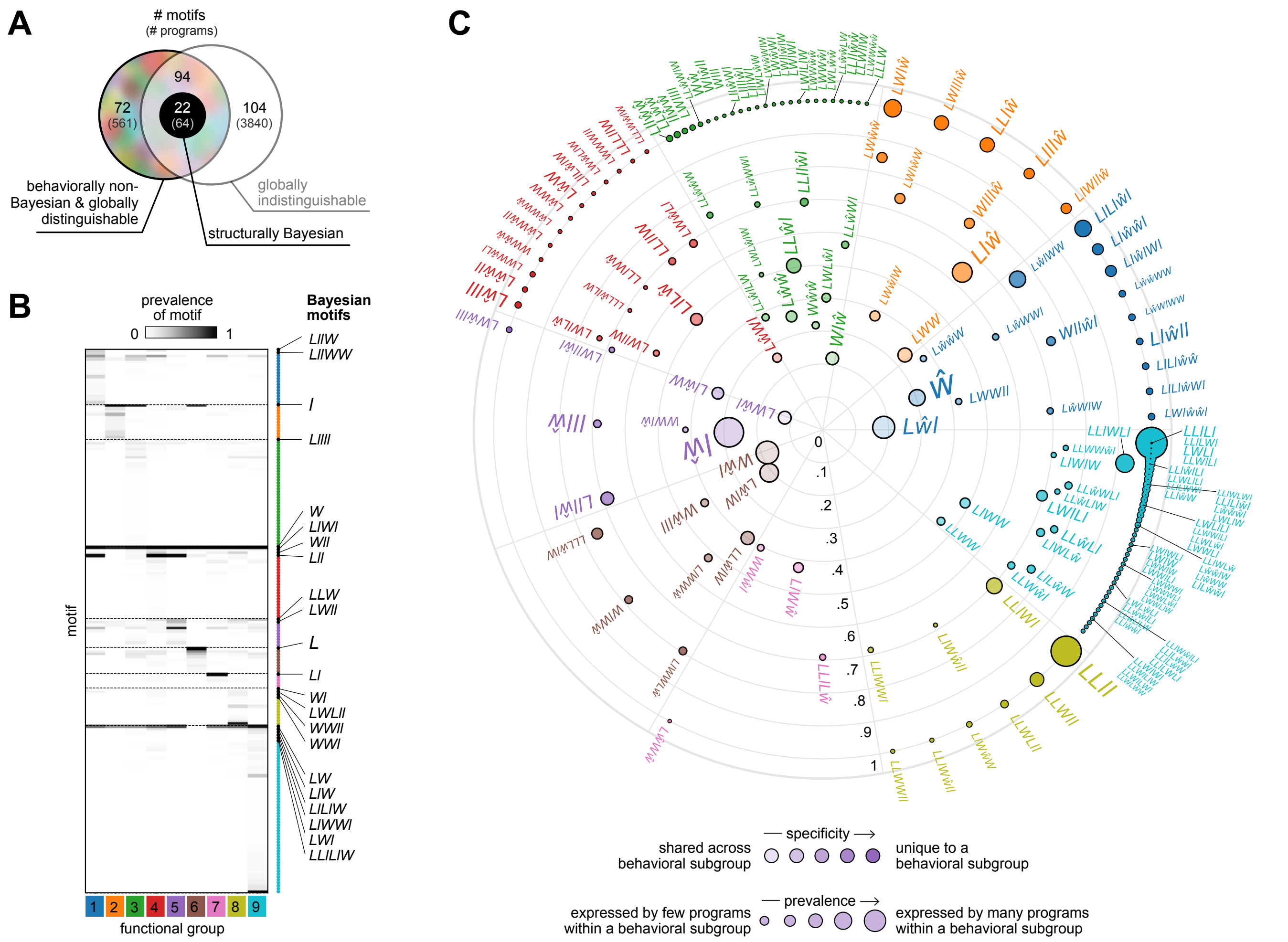

Figure S11: Globally-distinguishable programs generate a diversity of non-Bayesian motifs. (A) Numbers of motifs expressed by different groups of programs, copied from Fig. 4D. Here, we focus on the set of globally distinguishable, behaviorally non-Bayesian programs (colored circle in Venn diagram) that we previously clustered into 9 behavioral subgroups (see the inset of Fig. 3E for clustering). **(B-C)** Distributions of motifs expressed by each of the 9 behavioral subgroups. Individual motifs are assigned a color based on the behavioral subgroup for which they have the greatest prevalence. We compute this prevalence by determining the fraction of programs within a behavioral subgroup that are express a particular motif; the motif is then assigned to the group with the highest fraction. We use black to denote the motifs that are produced by the ensemble structurally-Bayesian programs, excluding one outlier (program 11; see Methods). **(B)** Summary of the occurrences of all 188 motifs expressed by the 9 behavioral subgroups (i.e., the 72 + 94 + 22 motifs within the colored circle in panel (A)). Each entry of the heatmap denotes the prevalence of each motif within each behavioral subgroup. Bayesian motifs are marked in black and listed to the right of the heatmap. Motifs are sorted first by behavioral subgroup, then by whether or not the motif is a Bayesian motif, then by the specificity of a motif for its assigned subgroup (Methods), and finally by the prevalence of the motif within the subgroup. **(C)** Summary of the occurrences of all 166 motifs expressed by the 9 behavioral subgroups, excluding the set of 22 Bayesian motifs. Each wedge highlights the motifs that distinguish a particular behavioral subgroup. The opacity and radial coordinate of each marker denotes the specificity of each motif for the given behavioral subgroup; high values of specificity indicate that programs are uniquely expressed within a given subgroup. The size of each marker denotes the fraction of programs within the subgroup that express the motif; larger markers correspond to motifs that are expressed by a large fraction of programs within a subgroup.

OVERVIEW

This supplementary text is structured in two parts: Part I provides a broader perspective on other methodologies that could be adopted to enumerate and study a space of strategies; Part II provides details of the current methodologies that we chose to adopt in the main text. We have intended for this to be readable as a standalone document, and as such, some of the information in Part II overlaps with, and expands upon, the main Methods.

Part I
IDEAS & PERSPECTIVES

In the following sections, we discuss alternative methodologies related to embedding algorithms, sloppiness, compositionality, and efficient search.

1 Why adopt a tree embedding algorithm?

1.1 Breaking down a large dataset into manageable chunks

One major goal of embedding a large dataset is to decompose it into smaller and more manageable chunks, in order to uncover relationships among individual entities in the dataset. In this way, the same embedding algorithm can be used to group entities based on different different attributes (e.g. “performance”, “structural complexity”, and “degree of functional difference from a Bayesian agent”), which can reveal different relationships between entities based on these attributes. For example, these embeddings might reveal relationships such as “structural complexity” does not predict “performance,” but “the degree of functional difference from a Bayesian agent” does. And since these common attributes are assigned to individual entities, one can simultaneously capture more detailed relationships among individual entities. Moreover, after establishing these relationships, one is left with multiple ways of grouping entities, and can thereby select and analyze each group separately.

1.2 Deciding which relationships to capture

For our purposes, a set of relevant attributes for describing a program can be grouped into the following:

1. attributes related to the *structure* of a program:
   (a) program size
   (b) number of mutations from a base program (e.g., a discretized version of Bayesian inference, the WSLG program, etc.)
   (c) wiring features (e.g., bidirectional integration, action-outcome loops, etc.)
2. attributes related to *function* of a program:
   (a) performance
   (b) joint state-belief occupancy
   (c) action-outcome statistics

Using the above attributes, our tree embedding (TE) algorithm uncovers the following relationships:

1. Nearly all *high-performing* programs are closely connected through single *structural mutations*;
2. A *mutation to a good program* is likely to create another *functionally similar* program;
3. A large *functional change* happens rarely, and follows a *key mutation*;
4. Nearly all *functionally-distinct* programs are clustered near leaf nodes, because it takes a several *key mutations from the root* to accumulate sufficiently many new behavioral sequences to become functionally distinct.

1.3 Capturing the nature of functional relationships between good programs

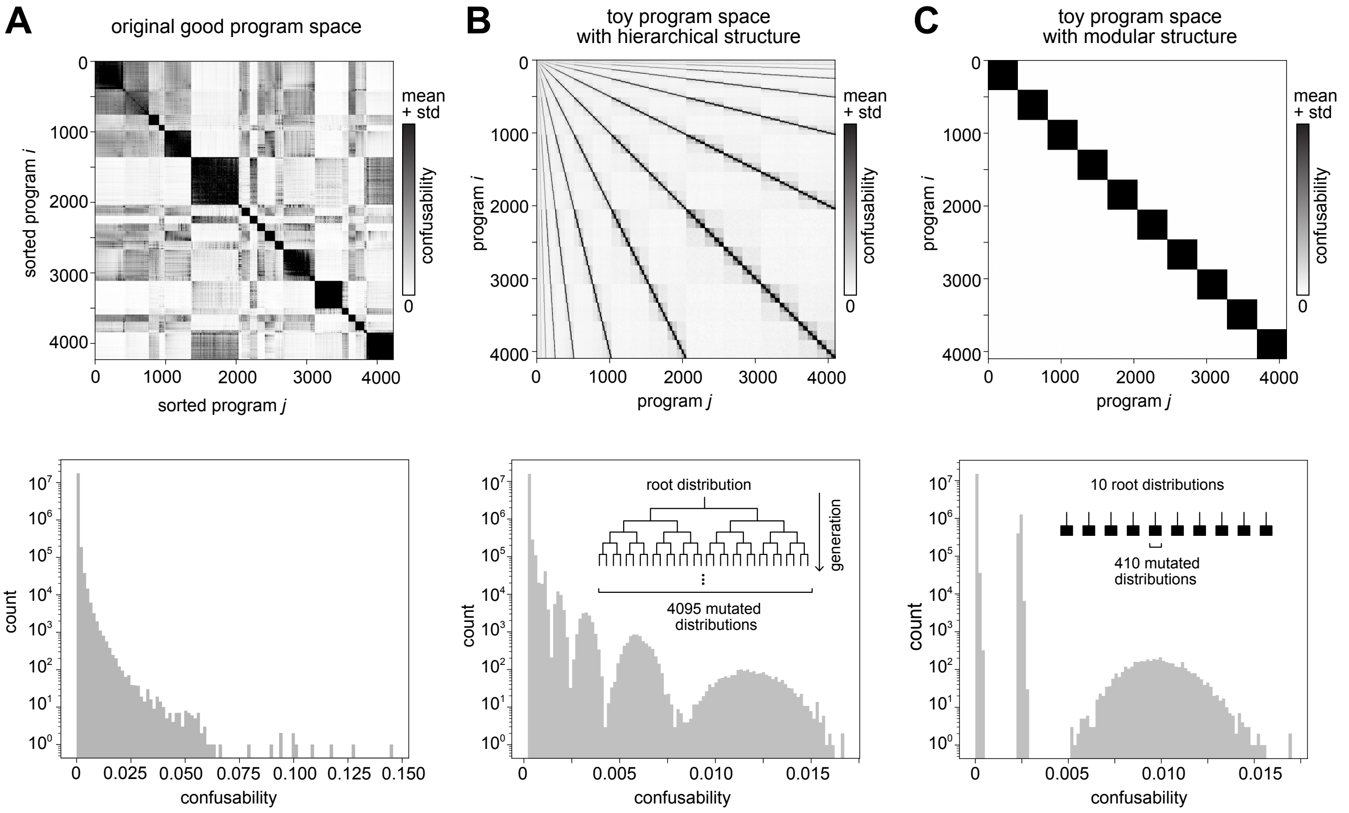

Figure S12: The space of good programs is hierarchical in nature. (A) Upper panel: The confusion matrix computed between behavioral sequences between all pairs of good programs, sorted with the `community_louvain` [55] clustering algorithm. The matrix is not fully block-diagonal, which indicates that the space is not fully modular. Lower panel: histogram of all values in the matrix. **(B)** A toy program space with tree-like structure contains 4095 hierarchically-connected distributions. This space produces a non-modular confusion matrix (upper panel), and the corresponding distribution of entries has a long tail (lower panel). **(C)** A toy program space with modular structure contains ten orthogonal groups, each with 410 programs (upper panel). The corresponding distribution of entries is multimodal, rather than long-tailed (lower panel). Note that for visualization purposes, we saturated the heatmap color at one std above the mean (computed over the set of values in each confusion matrix).

In this work, we adopted a tree embedding algorithm after discovering that the program space is inherently non-modular. To see this, we construct two toy program spaces (Figure S12): 1) a space with an *iterative branching structure* (i.e, a tree); and 2) a space with a *modular structure*. As we discuss below, the tree-like program space better captures the action-outcome statistics that we observe in the full program space.

The full program space is not modular. In Figure S12A, one can see that the confusion matrix computed from the action-outcome statistics of all 4,230 good programs does not have a clear block-diagonal structure, which suggests that the space of good programs cannot be broken down into a few mutually orthogonal clusters within which programs are functionally similar. To support this observation, one can see that the corresponding histogram (Figure S12A, lower panel) shows a continuous, long-tailed distribution. This long tail implies that (i) the size of program clusters, if they exist, could vary extensively (i.e., a larger cluster—within which most programs are confused with each other—would contribute to lower values of the distribution, whereas a smaller cluster would contribute to higher values); and (ii) most clusters, if they exist, could be partially mixed with neighboring clusters, yielding an overall distribution that is smooth. If the latter factor dominates relationships within the program space, it is not particularly helpful to view the space as modular.

A toy program space with hierarchical structure displays a long-tailed distribution of confusability values. Figure S12B illustrates the confusion matrix for a toy program space that is hierarchical in nature. To generate this

space, we grew a toy tree from a root program using a sparse random distribution of behavioral sequences. Next, we added small binary noise to the root distribution (`0+1=1, 1+1=0`) to generate two descendants. These two newly-generated distributions differ slightly from their parent. We apply this rule to iteratively mutate distributions up to 11 generations, thereby creating a space of $1+2+2^2+\ldots+2^{11}=4095$ programs. In Figure S12B, one can see a nested non-modular structure in the confusion matrix (upper panel), and each successive generation of programs creates a new lump in the corresponding distribution of confusability values (lower panel). This distribution closely resembles what we observe in the original space of good programs (Figure S12A, lower panel).

A toy program space with modular structure displays a multi-modal distribution of confusability values A counterexample is provided in Figure S12c, where 10 orthogonal distributions of behavioral sequences are seeded to generate an entire set of toy programs. By adding moderate noise to one of the distributions, we fill in one-tenth of the program space. By doing the same for remaining 9 distributions, we have a complete modular program space. To see how the histogram in the lower panel of Figure S12C comes to be, we can imagine a toy space without any additive noise. In such a space, all programs should have the same level of confusability within their own groups, and zero confusability between groups; this would manifest as two delta-function spikes at the locations of the left and middle peaks in the lower panel of Figure S12C. If one then adds noise to each cluster, some fraction of these two peaks will shift to the right, thereby forming an overall trimodal distribution (note that this trimodality is not visible in the heatmap because of the color range that we choose). This distribution much less resembles what we observe in Figure S12A than does the toy tree example

In retrospect, the lack of modular structure in the program space is not altogether surprising, given that several relevant program attributes operate within near-continuous domains. For example, the full program space is inherently unstructured because it includes all possible deterministic Markov chains. Moreover, for a large enough program space (268,536 programs with $M \leq 5$), a given task can be solved in nearly infinitely many ways, and thus the performance spectrum is effectively continuous. From these properties, one can infer that there is no obvious way to partition the program space into distinct clusters.

1.4 Tree embeddings with different objectives can capture different hierarchical structures

Knowing that the space of good programs is better described as hierarchical than modular, it is reasonable to perform a tree embedding on the program space to extract that hierarchical structure (in the form of relationships between pairs of programs). It's important to keep in mind that there could be more than one hierarchical structure embedded within the program space, and using a tree embedding with different objectives could reveal different hierarchical structures. For example, in the main text, we perform two different tree embeddings: one based on performance (TE), and another based on behavior (bTE).

Our tree embedding (TE) algorithm has two objectives for each child program: 1) preferentially find a parent program of a smaller size, and 2) preferentially find a parent program with a higher performance. Applying these two objectives reveals that programs with similar *performance* and *wiring* are hierarchically connected together (Figure 2D in the main text).

Our behavioral tree embedding (bTE) algorithm, on the other hand, has a different set of objectives for each child program: 1) preferentially find a parent program of a smaller size, and 2) preferentially find a parent program with a similar set of behavioral sequences. Applying these objectives reveals that most algorithmic mutations do not change program behavior (and are thus "sloppy"); however, a small set of "key" mutations scattered throughout the tree is sufficient to generate a large number of functionally-distinct programs near the leaf nodes of the tree.

1.5 Tree embeddings provide a useful way to make sense of a high-dimensional space

It is worth pointing out that by performing an embedding, one often forces a certain artificial structure onto their dataset. Although we argue that the inherent structure of the program space is hierarchical, the minimal relationships that are captured by TE or bTE are not guaranteed to fully capture the structure of the program space. For example, consider the fact that a tree has on average only two neighbors per node; one parent and one descendant. Individual

programs in the good program space, by contrast, can have between 2 and 340 single-mutation neighbors (with an average of 14 neighbors). Although our TE algorithm does not capture all of these relationships, it nevertheless has enabled us to extract several different insights from the program space, as discussed above.

More broadly, this type of tree embedding could provide a useful tool for making sense of other high-dimensional spaces, and could generate different insights compared to commonly-used clustering, dimensionality reduction, or embedding algorithms. This is because, in contrast to conventional clustering algorithms that typically reduce the dimensionality of a space by erasing the attributes of individual entities within the space, a tree embedding instead focuses on simplifying the relationships among entities while preserving their individual attributes. In this way, the goal of a tree embedding is to extract relational structures that could, in turn, comprise a set compact rules for iteratively generating the original space. As a result, it could be a more compatible approach for understanding a high-dimensional space in its entirety. In the main text, we used this idea to construct an evolutionary search algorithm that can efficiently discover good programs by searching only a fraction of the full space. In Section 4, we discuss another potential avenue for efficient program enumeration based on outcome-action motifs.

1.6 Comparisons to a commonly-used clustering algorithm, tSNE

Above, we discussed why conventional dimensionality reduction algorithms might be problematic in extracting relational structures in high-dimensional spaces. Here, we discuss one of our initial attempts to use tSNE to make sense of the program space. tSNE has well-known limitations; here, we discuss these limitations in the context of our program space, and we contrast this method with our tree embedding algorithms.

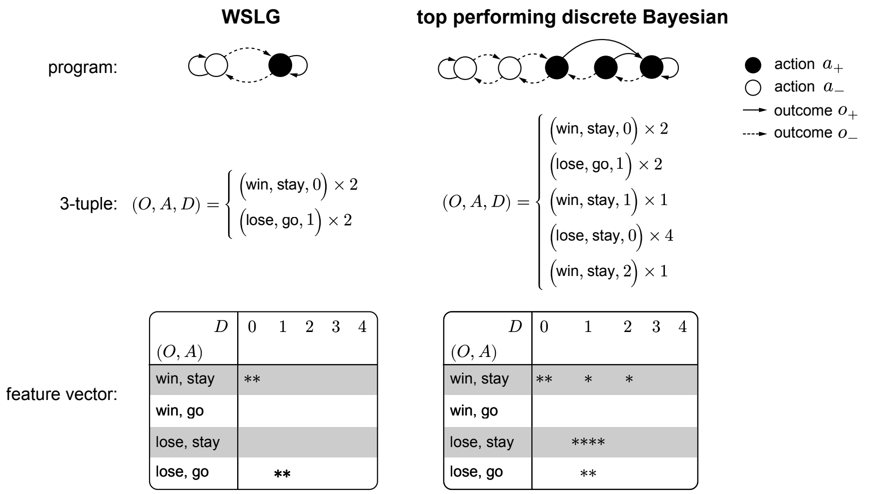

Figure S13: tSNE requires a formalized feature vector that is compatible with all program sizes. We construct a feature vector based on the wiring of each program (top row). This feature vector is constructed by enumerating all transitions in the program in terms of the action A that they originate from, the outcome O that they specify, and the distance D to the node on which they terminate (middle row). This vector can be viewed as a flattened version of a one-hot structure matrix (bottom row).

In order to use tSNE, we must first construct a feature vector that is identical in size despite the fact that programs will vary in size (note that this issue of program-size compatibility is something that we need to solve in designing our tree embedding as well; see Section 9.1 for details). To construct a compatible feature vector, we first note that every transition in a program can be labeled by a 2-tuple (O, A), where $O \in \{\text{win}, \text{lose}\}$, and $A \in \{\text{stay}, \text{go}\}$ (the WSLG program exemplifies this in its naming). However, this labeling alone is ambiguous, and it largely erases the wiring structure of a program (e.g., a "stay" transition can happen either through a self-loop, or if the destination node is labeled with the same action). To preserve more structural information, we instead use a 3-tuple (O, A, D) to label each transition. In this tuple, $D \in \{0, 1, 2, 3, 4\}$ measures the distance to a destination node, with $D = 0$ denoting a self-loop. To apply this labeling, we first find a specialized node ordering that minimizes the overall transition distance in the program; we refer to this overall transition distance as the "wiring length" of the program. Minimizing the wiring length ensures that all programs are as structurally similar as possible, such that the distance is most likely to capture real differences between the underlying graphs (invariant to different

representational choices; note that we don't have this problem in designing TE, since the number of mutations from one program to another is invariant across different representations). In Figure S13, we show examples of such 3-tuples and their corresponding feature vectors for two programs: the WSLG program, and the top-performing discrete Bayesian program.

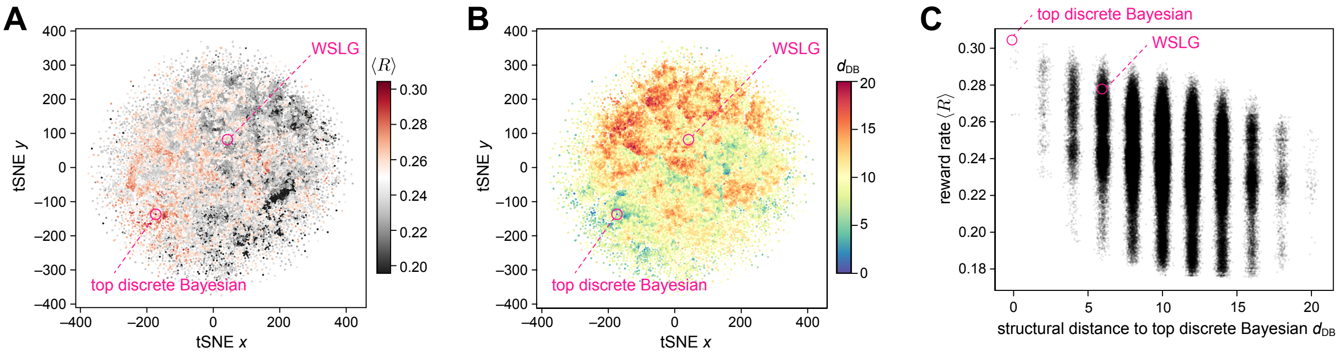

Figure S14: TSNE on the full program space. (A) The embedding with each program colored by its performance. **(B)** The embedding with each program colored by its structural distance from the top-performing discretized Bayesian program. **(C)** We observe a rough correlation between lower performances and larger distances from the top-performing discretized Bayesian program.

Figure S14 shows the results of tSNE, using the feature vector defined above. From these results, one can make a few immediate observations. First, without coloring the nodes, the embedding does not show a clear sign of distinguishable clusters. Second, while coloring each node by its reward rate or its distance to the top performing discretized Bayesian program reveals some intricate relationships between a program's structure and performance, the color separations are largely salt-and-pepper in nature. And finally, if a program is more structurally-similar to the top performing program (a discretized Bayesian program), it is more likely to perform well (note that this conclusion can be reached from Figure S14C, and does not rely on tSNE). From these results, one can see that tSNE serves as a useful first analysis step, and provides some intuition about the program space. In our case, tSNE shows that there is not any obvious clustering structure that can be used to break the program space into manageable chunks. This motivated us to construct a tree embedding algorithm, rather than to use or customize an existing clustering algorithm. In the table below, we summarize and compare some aspects of tSNE and TE applied to our problem:

	tSNE	TE
input	a list of programs with multiple attributes	a list of programs with multiple attributes
main embedding attribute	a formalized structure vector (note that this vector varies with different program representations)	a list of program IDs sorted by program size and performance
parameters	a list of initial tSNE coordinates, and a value of perplexity (note that results can vary greatly with a different initial condition or perplexity)	various schemes for sorting attributes (e.g., primary sort: program size; secondary sort: behavioral similarity; tertiary sort: performance)
output	a list of tSNE coordinates: $(x_{\text{tSNE}}, y_{\text{tSNE}})$	a list of edges: (program ID, parent ID)
distance in embedding	local distances are more informative; global distances are less informative	distances measured from a parent program to a direct descendant (child program) are informative; distances between two programs on different branches are less informative
clusters in embedding	tSNE is capable of extracting clusters if they exist in a dataset	TE does not partition a space by clustering entities based on their attributes; instead, it finds the minimal hierarchical relationships that link these entities

Table S1: Comparing tSNE with TE.

2 Notions of sloppiness, and how they could inform the study of generalizable strategies

In the main text, we focused on a single behavioral task, and we compared the performance and functionality of different behavioral strategies that solve this task in different ways. To study generalizability, one would need a complementary view of how performance and function persist under changes to the task. To explore this idea, we introduce the notion of sloppiness in either the space of behavioral sequences (functional sloppiness) or in the space of tasks (task sloppiness), with the goal of describing generalizability in these terms. But first, we discuss how generalizability could be understood by studying relational structures in a large solution space.

2.1 The value of studying relationships among good solutions

In the main text, we discussed how generalizability is often studied by constructing and dissecting a single versatile strategy that is capable of performing multiple tasks. While such an approach provides a realizable solution that can be immediately deployed to a practical problem, we argue that it offers limited insight into the behavioral features that enable generalizability.

To see this, consider searching for a strategy X that can jointly solve both Task 1 or 2 under some resource constraint. It is difficult to make any useful statements about generalizability without contrasting this strategy to another, even if this strategy is consistent with our intuition. To overcome this limitation, consider searching for two strategies Y and Z that can individually solve Task 1 and 2, respectively. Now if one contrasts strategies X and Y, or strategies X and Z, one can make a statement such as:

> "*Nearly all behaviors commonly shared between X and Y do not exist in Z, and vice versa; this suggests a strong tradeoff between implementing behaviors for Task 1 and those for Task 2. The relationship between these two tasks, and the minimal requirements needed to generalize across them, can be quantified in terms of this tradeoff.*"

From even this simplest example, one can already see the importance of studying relationships among different strategies if one wants to study generalizability. Building upon this argument, we believe that capturing various relational structures in both task and strategy spaces remains key to understanding generalizability in various problem domains. For this reason, we propose that an enumeration approach capable of discovering many good solutions, in combination with tools that can extract relational structures among those solutions, is best suited to study generalizable behavioral strategies.

2.2 Task sloppiness and functional sloppiness are closely related

When thinking about a single task and a single behavioral strategy, it is difficult to intuit how changing one affects another. In contrast, when both the task and the strategy are jointly parameterized, variations to these shared parameters create a coupled space of tasks and strategies. A common example is an Bayesian agent optimized on a parameterized task; in this case, both the Bayesian observer and the task are parameterized in the same way (for example, in our two-armed bandit task, the task and the optimal Bayesian observer are parameterized in terms of the hazard rate, reward contrast, and baseline reward rate). However, to go beyond a Bayesian observer, one needs to be able to link task and strategy spaces that do not share a common parameterization.

The tools and conceptual framework that we describe in the main text aim to enable such a connection. More specifically, we use an enumeration scheme to find the space of good behavioral strategies for a fixed task; we then perform Motif Decomposition (MD) on these strategies to create a *behavioral space* described in terms of the repertoire of each strategy. We can then morph the task to generate a *task space*, and we can repeat the above two steps to observe how the functional and task spaces relate to one another. Specifically, for a fixed task, we observe functional sloppiness when changes in behavioral repertoires do not compromise performance—we have observed this in the behavioral tree embedding, where one can traverse different lineages to reach a certain set of functions without compromising performance. Conversely, for a fixed behavioral repertoire, we expect to observe task sloppiness when changes to the task along do not compromise performance. Taken together, it could be

more sensible to talk about sloppiness in a joint task-function space in which many tasks map to many functions, and vice versa. In fact, this many-to-many mapping between tasks and functions resembles the mapping between perturbations and responses in robust biological systems [56].

2.3 Persistent axes in a high-dimensional function space hint at generalizable behaviors

To make these ideas more concrete, we use a minimal example to illustrate how the notion of sloppiness could relate to generalizability. In Figure S15, we plot the statistics of the lowest-order behavioral motifs (extracted from the ensemble of programs in the good program network), and we observe how the “shape” of the function space changes with variations to a single parameter of the task (here, the hazard rate). Conceptually, morphing a single aspect of this task can be viewed as moving toward a different region of the task space. A generalizable behavior then refers to a persistent set of functional axes in the function space. For example, “win-go” is a function that exists only transiently along this axis in task space, and thus would not generalize well, whereas “lose-stay” persists for much longer. In ongoing efforts, we are working to formalize this conceptual framework, and use it to better understand the origin of generalizability in terms of features that are shared among many biological computations and behavioral strategies.

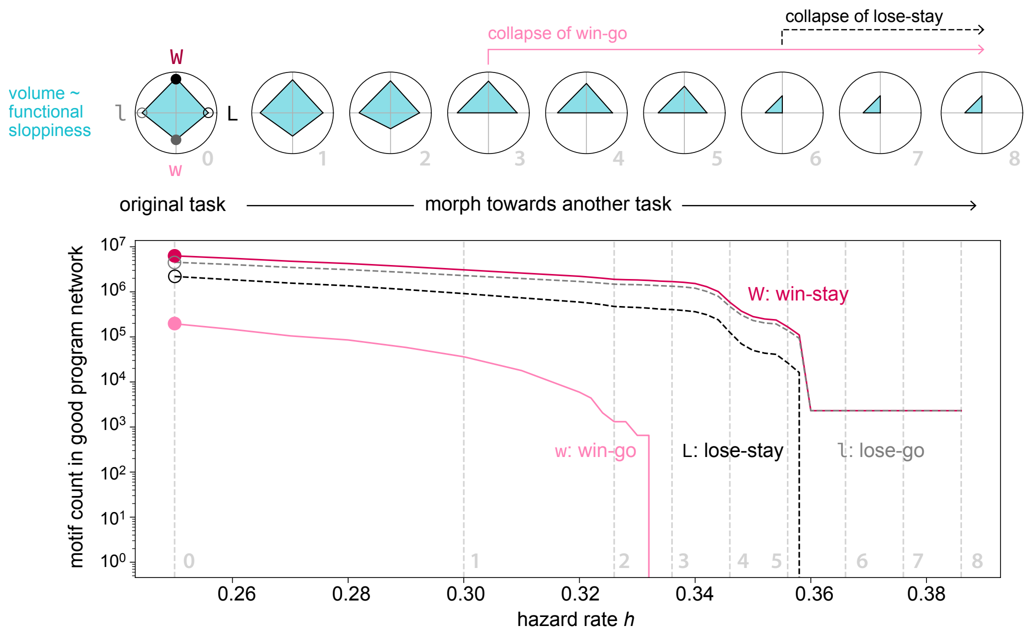

Figure S15: Generalizability could be understood in terms of how different functional axes vary with task structure. We use the statistics of the lowest-order motifs, observed within the ensemble of good programs, to capture a set of existing functions for a particular task (here, the same task and parameters used in the main text). We then morph this task by changing the hazard rate, and observe the relative persistence of these functions across changes in the task. The more persistent the behavior, the more it can generalize to different tasks along a particular task axis.

3 When and why to use Motif Decomposition (MD)

Motif Decomposition (MD) aims to bridge observed behaviors with the underlying rules that generate them. For example, in connectomics, the goal would be to link synaptic connectivity with neural activity. In the main text, we perform MD to bridge the structure a program (wiring) with its function (behavioral sequences). The details of the MD algorithm are discussed in Section 10.5. Here, we try to outline some insights that we obtained from performing MD on our problem.

3.1 Motifs bridge structure and function

Provided that we define a motif to be an "action-outcome loop" that can be observed in a long behavioral sequence, and provided that a loop is also directly depicted in the wiring of a program, it is straightforward to see how a change in the wiring of a program can alter the set of motifs that generate behavioral sequences. This enables us to understand how a single key mutation (a small change in structure) can lead to significant changes in the behavioral repertoire of a program. However, it is import to point out that this reasoning does not necessitate using loops as motifs. Alternatively, one might perform a decomposition using all possible short sequences with a fixed length—i.e., n-grams—as illustrated in Table S2:

example motifs as loops	example motifs as bigrams
`W`	`LL`
`LW`	`Ll`
`l`	`LW`
`Lll`	`Lw`
`LLW`	`ll`
`LllW`	`lW`
`Lwl`	`lw`
`lw`	`WW`
`Wwl`	`Ww`
`LWW`	`ww`

Table S2: example motifs and bigrams. `W`: win-stay, `L`: lose-stay, `w`: win-go, and `l`: lose-go.

However, the somewhat arbitrary choice of bigrams in Table S2 does not explicitly take advantage of the structure of behavioral sequences, and is thus a highly redundant representation of behavior. In Section 3.3, we will quantify this redundancy when we discuss motif compositionality.

3.2 The utility of Motif Decomposition

The reason that we find loops provide such an effective decomposition of behavioral sequences is in part because 1) we consider programs that are relatively small in size ($M \leq 5$), and 2) we consider a task that is relatively stable over time (hazard rate $h = 1/20$). Under these conditions, nearly all small loops through a program can be reached relatively quickly, and therefore a long behavioral sequence can be decomposed into a set of short loops (a majority of which are less than or equal to length=5). As a result, loops provide a highly compressed representation that can capture a majority of the behaviors we observe without significant loss of information. However, if the above two criteria are not met, the specific motif decomposition that we adopt is not particularly useful. For example, a large dataset of short video clips might predominantly consist of a set of short open sequences rather than loops; such a dataset require a very different decomposition algorithm to extract open-ended sequence as motifs. Similarly, decomposing an ensemble of static images into a set of linearly-combined feature vectors can be better addressed by using a deep feedforward neural network.

3.3 Structured compositional motifs constrain task sloppiness

In the main text, we used Motif Decomposition (MD) to enumerate different ways of constructing a good program from a set of motifs (here, action-outcome loops). This revealed different degrees of sloppiness in terms of the multiple ways that different program structures could produce the same motifs, and the multiple ways that these motifs could be combined to generate behavioral sequences. This finding relies on the *compositionality* of the space, defined in terms of the many different ways of constructing a *composite* from a set of *components*. In the discussion below, we compare MD with a commonly used n-gram analysis to show how MD generates motifs that efficiently capture the set of behaviors produced by the ensemble of good programs. We then discuss how compositional relationships among motifs constrain the degree of functional sloppiness—i.e., the number of good solutions—for this particular task.

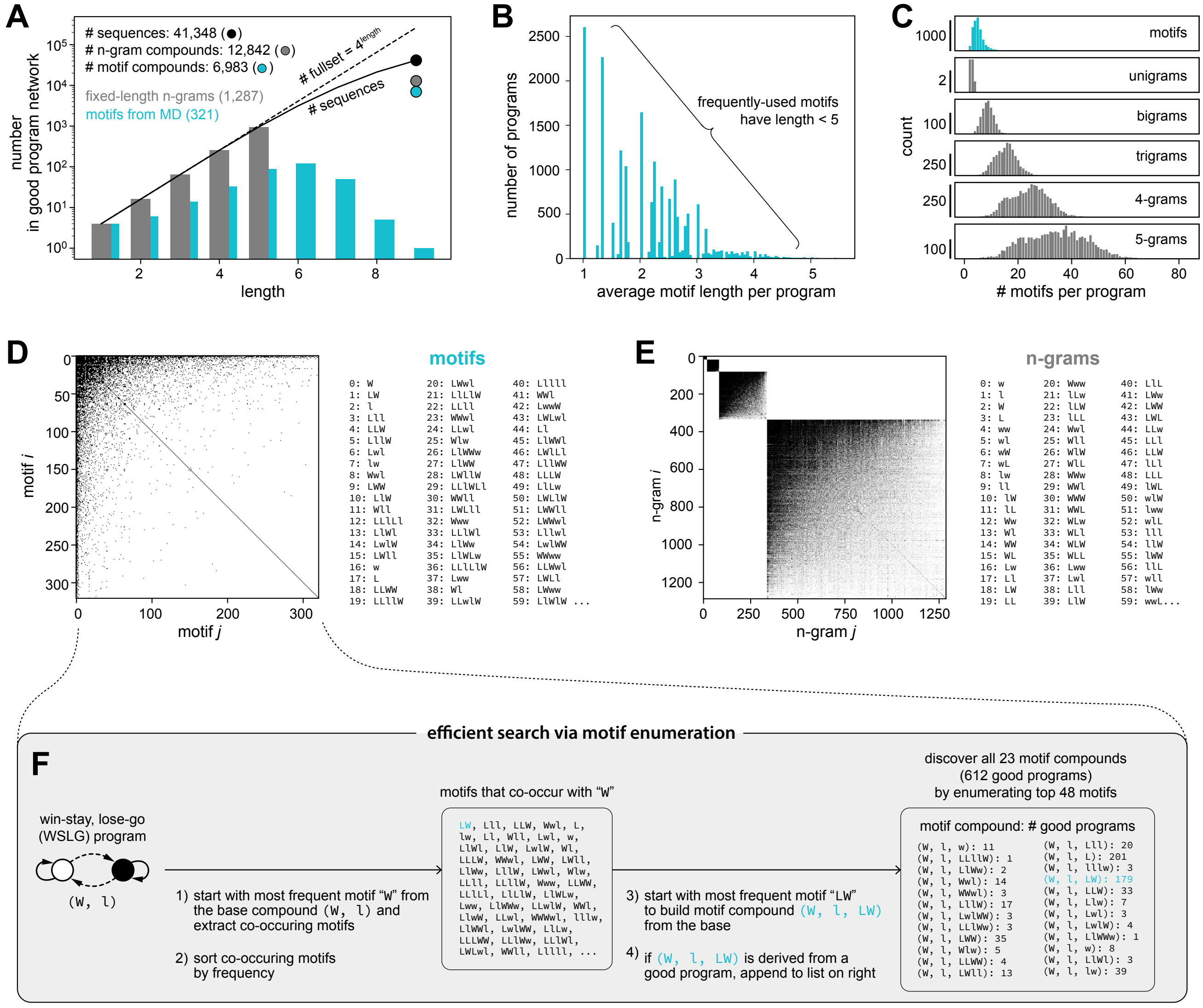

Figure S16: Motif Decomposition (MD) efficiently captures the behavioral repertoire of good programs and enables the exploration of a larger program space. (A) The set of motifs extracted from MD result in a more efficient representation of the good program space than do sets of n-grams. **(B)** We use the statistics of motif lengths to select the set of n-gram lengths. **(C)** A program can be represented with just a few motifs, in comparison to representing it with n-gram. **(D-E)** A sparse co-occurrence matrix for motifs shows that a compositional rule for motifs is highly structured. In contrast, such a rule for n-gram is much less structured. **(F)** The structured compositional rules for motifs could enable an efficient search via motif enumeration.

Motif Decomposition (MD) efficiently captures the structure of the good program space. To get a sense of how functional sloppiness might relate to the compositionality of motifs, one needs to ensure that a set of motifs (here, termed a "motif compound") does indeed faithfully and efficiently represent a behavioral sequence. If this representation is highly redundant, it could give the false impression that the space of motifs is larger than it actually is. Here, we demonstrate this by contrasting the results from MD with those obtained from an n-gram analysis.

n-gram analysis. n-gram analysis, commonly used in language processing, is a generic approach for decomposing a long string of text into multiple manageable statistical distributions. Here, we use this approach to decompose a long behavioral sequence into distributions over shorter elements of that sequence. We can start by constructing 1st and 2nd-order unigram and bigram statistics:

	unigram	bigram
1st-order statistic	$p\,(\text{seq})\ \forall\text{seq} \in \{L, W, l, w\}$	$p\,(\text{seq})\ \forall\text{seq} \in \{LL, Ll, LW, \ldots, ww\}$
2nd-order statistic	$p\,(\text{seq}\ i,\ \text{seq}\ j)$ $\forall\,(\text{seq}\ i,\ \text{seq}\ j) \in \{(L, L), (L, W), \ldots\}$	$p\,(\text{seq}\ i,\ \text{seq}\ j)$ $\forall\,(\text{seq}\ i,\ \text{seq}\ j) \in \{(LL, LL), (LL, Ll), \ldots\}$

Table S3: Example of unigram and bigram statistics.

As one can see, unigrams and bigrams have limited expressive power in capturing the complexity of all program behaviors. To improve the granularity of these descriptions, one can consider higher-order n-grams. As shown in Figure S16, we consider up to 5-grams (this is because nearly all of the frequently-used motifs are of lengths less than or equal to 5; see Figure S16B). Note that for each program, there is a small set of possible n-grams that could exist with a nonzero probability; this set of n-grams serves as the lowest-order description for a program. The next higher-order description can be expressed as a correlation matrix for nonzero co-occurrences between two existing n-grams, as shown in Figure S16E. After sweeping from unigrams to 5-grams and from lowest-order to higher-order statistics, each program can then be collectively described by a set of n-gram statistics.

Motifs are highly efficient in capturing the behavior of small programs. Using this approach, we can count how many unique n-grams are required to describe the entirety of the good program space, and we can compare that to the results obtained from MD. In Figure S16A, one can see that the total number of n-grams (1,287) that one needs to capture the good program space is much larger than the total number of motifs (321), indicating that MD provides a more concise description of the space.

Next, we focus on the compositional properties of these n-grams by counting the number of different ways that n-grams can be combined to summarize a program. n-grams can be combined to form 12,842 possible compounds; motifs, on the other hand, form 6,983 compounds (note that for motifs, we count how many compounds are used to describe the sequences produced by a program). Once again, this comparison shows that MD provides a more efficient description than do n-grams. Lastly, at individual program level, one can use far fewer motifs to describe any given program (Figure S16C).

It's worth noting that MD provides a more efficient description of the good program space because it exploits the fact that all long sequences are built from a small number of relatively short action-outcome loops. MD uses this knowledge of the space to compress the set of behavioral sequences (summarized by 41,348 sequences in total) down to a much more concise description (summarized by 6,983 compounds in total).

3.4 Motif compositional rules are highly structured

From the results discussed above, one gets a sense about how structured the compositional rules can be for combining motifs or n-grams into permissible compounds. For example, there could in principle be up to 51,360 ways to combine two motifs, and 5,461,280 ways to combine three motifs. Yet, the observed rules only allow 6,983 motif compounds to describe the good program space. Here, we take a closer look at the properties of these compositional rules. In Figure S16D-E, one can see that the motif co-occurrence matrix is highly sparse and skewed (a few motifs co-occur with many, while most motifs co-occur with a few), whereas the n-gram co-occurrence matrix is much denser. This comparison implies that the compositional rules for building a motif compound are highly structured. Note that the top co-occurring motifs have mixed lengths ranging from 1 to 5; such mixed-length compositional structures are absent in our n-gram analysis, which could contribute to the inefficiency of an n-gram description.

3.5 Multi-layered compositional structures constrain the good solution space

To conclude this section, we examine how compositional structures among motifs constrain the size of good program space. A full analysis of motif statistics is beyond the scope of this work, but we hope to provide the main intuition behind our observations. Below, we list some examples about the compositional properties of motifs and compounds.

From wiring to motif

- A set of motifs, is used as a functional fingerprint to capture the wiring of a program. Most programs can be described as just a few motifs (Figure S16C).
- An identical set of motifs can represent many structurally different programs—e.g., 130 good programs can be described by (W, l), which is also a description of the 2-state WSLG program.

From motif to compound

- Not only are motifs compositional, but many compounds are as well—e.g., a compound (W, l) that represents a specific program can be further combined with 23 other motifs to describe another good program (see Figure S16F motif enumeration for examples).

From compound to sequence

- An identical sequence can be described by different compounds—e.g., a sequence "$LlWlWLllL$" (which exists in 201 good programs) can be described in 25 different ways:

$$LlWlWLllL \xrightarrow{\text{MD}} \begin{cases} (L,\ W,\ Lll) \\ (L,\ W,\ Lll,\ LlWl) \\ (L,\ W,\ Lll,\ Wll) \\ (W,\ LW,\ LlWl,\ LLll) \\ (W,\ LW,\ Lll) \\ (W,\ LW,\ Lll,\ LlWl) \\ (W,\ LW,\ Lll,\ LlWl,\ LllW) \\ (W,\ LW,\ Lll,\ LlWlW) \\ (W,\ LW,\ Lll,\ LllW) \\ (W,\ LW,\ Lll,\ WWl) \\ (W,\ LW,\ Lll,\ WWl,\ LlWl) \\ (W,\ LW,\ Lll,\ Wll) \\ (W,\ LW,\ Lll,\ Wll,\ LlWlW) \\ (W,\ LW,\ WWl,\ Lll) \\ (W,\ LW,\ Wll,\ LLll) \\ (W,\ LW,\ Wll,\ LLll,\ LlWlW) \\ (W,\ LW,\ Wll,\ Lll) \\ (W,\ Lll,\ LlWl) \\ (W,\ Lll,\ LlWl,\ LllW) \\ (W,\ Lll,\ LllW) \\ (W,\ Lll,\ WWl,\ LlWl) \\ (W,\ WWl,\ LLll) \\ (W,\ l,\ LW) \\ (W,\ l,\ LW,\ Lll) \\ (W,\ l,\ Lll) \end{cases}$$

Taken together, one can see how these multi-layered, compositional structures constrain the space of good solutions. In the next section, we provide a potentially powerful approach for exploring a much larger solution space using a constrained enumeration that leverages this compositional structure.

4 Efficient search via Motif Enumeration

In the previous section, we illustrated how Motif Decomposition could provide a compact description of program behaviors, and we showed how it can be used to understand several different sources of sloppiness within the ensemble of good programs. In this final section, we sketch out how motifs could be used to construct another class of efficient search algorithms, beyond the evolutionary algorithm that we demonstrated in the main text (see Section 9.4 for details).

4.1 A search that enumerates behaviors instead of wiring structures

There are two important differences between enumerating in the space of motifs, and in the original space of programs. First, in program enumeration, one does not add any additional constraints (beyond a resource constraint that limits number of program states) that could bias the enumeration; in motif enumeration, one takes advantage of the structure of good programs to search more efficiently. Second, in program enumeration, one enumerates the wiring directly; in motif enumeration, one enumerates at a level that is closer to behavior. Motif enumeration thus requires a mapping from behavioral motifs back to the wiring of a program. In the discussion below, we assume that such a mapping exists and can be performed efficiently, and we focus instead on the diversity that emerges at the level of motifs.

4.2 Leveraging known functions to constrain an enumeration could enable exploration in a much larger solution space

In the previous section, we showed that the compositional structures of motifs can be complex (e.g., there exist additional higher-order co-occurrence matrices, beyond the pairwise one that we show in Figure S16D), and thus translating those structures into an efficient search algorithm is in itself an interesting and challenging problem. Below, we use a minimal example (Figure S16F) to demonstrate how such a search could be performed in principle:

1. One could start from the WSLG program, with its motif compound $(W,\ l)$.
2. For an individual motif, one could then list and sort the corresponding co-occurring motifs based on their frequency—e.g., "LW," "Lll," and "LLW" are the top three motifs that co-occur with "W."
3. Next, one could concatenate these motifs to form new compounds—e.g., $(W,\ l) + LW \ \rightarrow\ (W,\ l,\ LW)$, etc.
4. Each new compound now represents a set of new programs (assuming the mapping from a compound to a program can be performed efficiently) that can be readily evaluated.

As shown in Figure S16F, after searching only 48 of the top co-occurring motifs, this algorithm finds all 23 existing length-3 compounds that cover 612 good programs (i.e., that cover 14% of the good program space in a single step).

Note that in this example, we only construct a relatively small compound, which is likely to represent a relatively small program. One can imagine using a larger compound as a starting point, or using a more elaborate enumeration rule to output a larger compound. This approach then presents an intriguing opportunity to enumerate towards a much larger solution space (with programs that have many more states, beyond $M = 5$). Since both motifs and their compositional rules are derived directly from the enumerated set of good programs, motif enumeration leverages the structure in a known space of good program space to constrain the search in an unknown space. Moreover, by separating the search into two steps—first, enumerating and evaluating behavioral motifs, and second, finding realizable programs for each motif compound—the search at each step could yield a much smaller enumeration that is less computationally costly.

Taken together, motif decomposition and motif enumeration could provide one set of approaches for exploring, analyzing, and understanding relationships within large solution spaces. The insights gained from this exploration could then be leveraged and translated into a generative search algorithm to discover new and previously unreachable solutions, which can in turn be used to gain a deeper understanding of the space of good solutions. It remains to be seen how such bootstrapping should best be constructed, and what level of understanding can be reached in different problem domains spanning biology, neuroscience, cognitive science, or AI.

Part II
MATHS & CODES

In the following sections, we provide a detailed discussion of the methods that were used in the main text. This is intended to be readable as a standalone document; as a result, some of the information is repeated from the main Methods.

5 Bayesian formalism

In this section, we discuss all approaches related to the Bayesian formalism, including how one can leverage the concise description of a Bayesian agent to efficiently find a small set of good resource-limited programs, which we term discretized Bayesian (DB) agents. At the end of this section, we discuss how it is possible, from the Bayesian perspective, to have a complex nonlinear scaling between the size of the good program space and a given task parameter, even when optimal Bayesian performance changes smoothly with respect to that same parameter.

5.1 Bayesian inference

Task structure. A behavioral task can be described by two parameterized conditional probability distributions: 1) the world dynamics, and 2) the reward delivery. We refer to these two probability distributions as the "task structure".

World dynamics	Reward delivery
$p(s_t \mid s_{t-1}) = \begin{pmatrix} 1-h & h \\ h & 1-h \end{pmatrix}$	$p(o \mid s, a) = \frac{1+o\ (s\ a\Delta p+\Delta\overline{p})}{2}$

Table S4: Task structures.

where all variables and parameters are listed below:

Three binary variables	Task parameters	Utility
action: $a \in \{a_-, a_+\}$<br>outcome: $o \in \{o_-, o_+\}$<br>world state: $s \in \{s_-, s_+\}$ (hidden)	reward gain: $\Delta\overline{p} \in [-1,\ 1]$<br>reward contrast: $\Delta p \in [0,\ 1-\lvert\Delta\overline{p}\rvert]$<br>hazard rate: $h \in [0,\ 0.5]$	$\max_\pi \langle o = o_+ \rangle$

Table S5: Task variables, parameters, and utility functions.

Note that from the table above, the reward gain $\Delta\overline{p} \equiv 2\overline{p} - 1$ is defined as a centered and normalized baseline reward rate $\overline{p}$.

Using Bayesian inference to map from task structure to an iterative belief update. A single step in a Bayesian inference algorithm involves using a single piece of evidence of update an internal belief about an external state of the world. This statement can be described as follows:

$$
\begin{aligned}
\underbrace{p(s_t \mid a_<, o_<)}_{\text{posterior at } t} &= \sum_{s_{t-1}} p\left(s_t \mid s_{t-1}, \cancel{a_<}, \cancel{o_<}\right)\ p(s_{t-1} \mid a_{t-1}, o_{t-1}, a_<, o_<) && \text{chain rule + marginalization} \\
&= \sum_{s_{t-1}} p(s_t \mid s_{t-1})\ \frac{1}{\Omega}\ p\left(o_{t-1} \mid s_{t-1}, a_{t-1}, \cancel{a_<}, \cancel{o_<}\right)\ p\left(s_{t-1} \mid \cancel{a_{t-1}}, a_<, o_<\right) && \text{Bayes' rule} \\
&= \sum_{s_{t-1}} \underbrace{p(s_t \mid s_{t-1})}_{\text{world dynamics}}\ \frac{1}{\Omega}\ \underbrace{p(o_{t-1} \mid s_{t-1}, a_{t-1})}_{\text{reward delivery}}\ \underbrace{p(s_{t-1} \mid a_<, o_<)}_{\text{prior at } t-1}
\end{aligned}
\tag{S1}
$$

Since $p(s_t = s_+ \mid \dots) + p(s_t = s_- \mid \dots) = 1$, one only needs to keep track of a one-dimensional *belief value*:

$$u_t \equiv p(s_t = s_+ \mid \dots) - p(s_t = s_- \mid \dots) \in [-1, 1] \tag{S2}$$

Substituting the task structure into Equation (S1) yields the following iterative belief update:

$$\boxed{\begin{aligned} & u \leftarrow U(u, a, o) \\ \text{where} \quad & U(u, a, o) \equiv (1 - 2h) \cdot \frac{a\ o\ \Delta p + (1 + o\Delta\overline{p})\ u}{a\ o\ \Delta p\ u + (1 + o\Delta\overline{p})} \end{aligned}} \tag{S3}$$

Note that in the above expressions, we use the following simplified notation for all binary variables:

$$\begin{aligned} a &\in \{a_-, a_+\} = \{-1, 1\} \\ o &\in \{o_-, o_+\} = \{-1, 1\} \\ s &\in \{s_-, s_+\} = \{-1, 1\} \end{aligned} \tag{S4}$$

5.2 Bayesian reinforcement learning problem

A Bayesian reinforcement learning (RL) problem is a factorization of a behavioral task into the two separate problems of 1) deriving an optimal inference and 2) finding an optimal policy. The former can be done using Bayesian formalism, as discussed above. The latter requires optimization. Fortunately, this problem is equivalent to a standard RL problem with a fully observable Markov Decision Process (MDP) over (infinite) belief states (these belief states can be thought of as a fine-grained discretization of the belief value in Equation (S2)). With this equivalence, a standard RL algorithm such as value iteration can be used to efficiently find the optimal policy.

Mapping the task to an RL problem. Given knowledge of the task, a Bayesian agent uses Bayesian inference, via Equation (S3), to recursively update its belief:

$$p(s' \mid s, a, o) \xrightarrow{\text{mapping with Bayesian formalism}} p(u' \mid u, a, o) \tag{S5}$$

The remaining problem is to find a policy that optimizes cumulative reward:

$$\max_{\pi} \langle o = o_+ \rangle \text{ with } \pi(a \mid u) \text{ and } u \in [-1, 1] \tag{S6}$$

Equation (S5) and Equation (S6) can be then mapped to a standard RL problem with a fully-observable state space u:

$$\begin{aligned} p(u', o \mid u, a) &= \underbrace{p(u' \mid u, a, o)}_{\text{belief update}} \times \underbrace{p(o \mid u, a)}_{\text{outcome probability}} && \text{MDP} \\ &= \delta\left(u' - (1 - 2h) \cdot \frac{a\ o\ \Delta p + (1 + o\Delta\overline{p})\ u}{a\ o\ \Delta p\ u + (1 + o\Delta\overline{p})}\right) \times \frac{1 + o\ (a\ \Delta p\ u + \Delta\overline{p})}{2} \\ r(o) &= \begin{cases} 1 & \text{if } o = o_+ \\ 0 & \text{if } o = o_- \end{cases} && \text{reward function} \\ & q(u, a) && \text{q-value function to be optimized} \\ a &\leftarrow \operatorname*{argmax}_a q(a, u) && \text{greedy policy} \end{aligned} \tag{S7}$$

where the outcome probability can be derived from the original *reward delivery* in the task structure:

$$
\begin{aligned}
p(o \mid u, a) &= \sum_s \underbrace{p\,(o \mid s, \not{u}, a)}_{\text{reward delivery}} \; \underbrace{p\,(s \mid u, \not{a})}_{=p(s \mid a_<, o_<)} \\
&= \sum_s \frac{1 + o\,(s\;a\;\Delta p + \Delta\overline{p})}{2} \cdot \frac{1 + s\;u}{2} \\
&= \frac{1 + o\,(a\;\Delta p\;u + \Delta\overline{p})}{2}
\end{aligned}
\tag{S8}
$$

Value Iteration. In order to use RL to derive an optimal policy, one needs to discretize a continuous belief value into finite number of belief states. Here, we use 200 non-overlapping, equally-sized bins to tile the belief space: $u \in [-1, 1]$. Since our problem is relatively small, it is efficient to use value iteration to optimize the policy:

$$
v(u, t+1) = \max_a \underbrace{\sum_{u', o} p(u', r(o) \mid u, a) \left[\frac{r(o)}{t+1} + \frac{t}{t+1} v(u', t)\right]}_{q(u,a)}
\tag{S9}
$$

Note that here, we modify standard value iteration [29] with a built-in running average of reward over an infinite horizon. The optimal policy is then:

$$
\begin{aligned}
a^* &= \operatorname*{argmax}_a q(u, a) \\
&= \operatorname{sgn} u
\end{aligned}
\tag{S10}
$$

This optimal policy corresponds to a naive reward-seeking policy:

$$
\begin{aligned}
a_{\text{rewardseek}} &\equiv \operatorname*{argmax}_a \langle r(o \mid u, a) \rangle \\
&= \operatorname*{argmax}_a p(o = o_+ \mid u, a) \times 1 \\
&= \operatorname*{argmax}_a \frac{1 + a\;\Delta p\;u + \Delta\overline{p}}{2} \\
&= \operatorname{sgn} u
\end{aligned}
\tag{S11}
$$

Given that the optimal policy does not change with task parameters, the particular two-armed bandit task that we study here is relatively simple from the Bayesian perspective. It is for this reason that we were surprised to find that modifying the Bayesian inference machinery can induce such complexity in the observed solution space.

5.3 Discretizating the optimal Bayesian agent

The point of discretizing a Bayesian agent is to 1) qualitatively capture Bayesian behavior with finite resources, and 2) derive an efficient search algorithm that can identify good solutions without full enumeration. With these goals in mind, we sought to avoid excessive details in our discretization algorithm that might dilute the key behavior of the Bayesian computation, or that might lead to excessive coverage of the full program space.

To preserve the essence of the Bayesian computation, we directly discretized the one-dimensional Bayesian belief value into $M \in \{2, 3, 4, 5\}$ equal bins, and we derived the finite-state transition matrix by finding the discrete state m that is closest to a transitioned belief value:

$$
\begin{aligned}
u \in [-\alpha u_{ub}, \alpha u_{ub}] &\xrightarrow{\text{uniformly discretized}} m \in \{m_0, m_1, \ldots, m_{M-1}\} \\
u \leftarrow U(u, a, o) &\xrightarrow{\text{transition into closest state}} m \leftarrow U_{\text{DB}}(m, a, o)
\end{aligned}
\tag{S12}
$$

where $\alpha \in (0, 1]$ is a *sweeping parameter* that controls the full range of discretization, and

$$u_{ub} = \sqrt{(1-2h) + \left(\frac{1+\Delta\overline{p}}{\Delta p}h\right)^2} - \frac{1+\Delta\overline{p}}{\Delta p}h < 1 \quad \forall h \in (0, 0.5] \tag{S13}$$

is the belief fixed-point upon continual winning (see Section 5.5 for derivation).

The last step in constructing a discretized Bayesian (DB) agent is to attach a deterministic action to each state m (note that the optimal policy found via value iteration is deterministic). Here, we simply use the optimal (reward-seeking) policy:

$$a(u) = \operatorname{sgn} u \quad \xrightarrow{\text{simple mapping}} \quad a(m) = \operatorname{sgn} u_m \tag{S14}$$

where u_m is the corresponding belief value of the discrete state m. This discretization process generates a handful of DBs that are then filtered by a set of "rule-out rules" that eliminate invalid DBs (discussed in detail in Section 7.2).

5.4 Constrained enumeration for discretized Bayesians (DBs)

The uniform discretization discussed above can lead to ambiguities if a belief transition happens to fall between two discrete states. Simply picking the nearest state may result in unwanted bias in the resulting DBs. To counter this bias, we considered all combinations of nearest and next nearest state pairs when constructing DBs. For example, consider enumerating nearest and next nearest state pairs in a three-state DB:

$$\text{DB\_0}: \begin{cases} m_0 \xrightarrow{\text{upon winning}} (m_0, m_1) \\ m_0 \xrightarrow{\text{upon losing}} (m_1, m_2) \\ \quad \ldots \end{cases} \xrightarrow{\text{enumerate pairs}} \begin{cases} \text{DB\_1}: \begin{cases} m_0 \xrightarrow{\text{upon winning}} m_0 \\ m_0 \xrightarrow{\text{upon losing}} m_1 \end{cases} & \text{DB\_2}: \begin{cases} m_0 \xrightarrow{\text{upon winning}} m_0 \\ m_0 \xrightarrow{\text{upon losing}} m_2 \end{cases} \\ \text{DB\_3}: \begin{cases} m_0 \xrightarrow{\text{upon winning}} m_1 \\ m_0 \xrightarrow{\text{upon losing}} m_1 \end{cases} & \text{DB\_4}: \begin{cases} m_0 \xrightarrow{\text{upon winning}} m_1 \\ m_0 \xrightarrow{\text{upon losing}} m_2 \end{cases} \end{cases} \tag{S15}$$

This enumeration results in a larger ensemble of DBs that all retain the bi-directional integration (i.e., the inherent node ordering for a monotonic belief update upon consecutive winning or losing) that is characteristic of the original Bayesian inference.

To see how Bayesian performance scales with the available resources (states), we enumerate and evaluate all DBs up to $M = 8$. Table S6 below shows that the performance rapidly saturates with increasing numbers of states—indicating that this task can be solved with far fewer resources than are required by the optimal Bayesian agent. This type of calculation can be used to measure task complexity, something that is not informed by the optimal Bayesian agent.

M	reward rate	optimality
2	0.277911	0.878
3	0.288177	0.911
4	0.298633	0.944
5	0.304524	0.963
6	0.310728	0.982
7	0.313015	0.989
8	0.31534	0.997
∞	0.31638	1

Table S6: Increasing performance upon increasing size of discretized Bayesian programs.

5.5 Fixed point analysis of the optimal Bayesian agent

The aim of this fixed point analysis is to categorize the behavior of an optimal Bayesian agent in different regions of the task space; we will build on this analysis in the next section. Here, we sweep the hazard rate while fixing the reward gain and reward contrast:

$$\text{task space} \equiv \begin{cases} \Delta p = 0.3 \\ \Delta \overline{p} = -0.5 \\ \quad h \in [0.05,\ 0.5] \end{cases} \tag{S16}$$

Special belief values $\mathbf{u}$. There are five special belief values that enable us to categorize Bayesian behaviors, defined in terms of the belief update function U given in Equation (S3) (see Figure S17 for a graphical description of these belief values):

definition	solution	name
$U(u_{ub}, a_+, o_+) = u_{ub}$	$u_{ub} = \sqrt{(1-2h) + \left(\frac{1+\Delta\overline{p}}{\Delta p}h\right)^2} - \frac{1+\Delta\overline{p}}{\Delta p}h$	belief upper bound
$U(u_{cr}, a_+, o_-) = 0$	$u_{cr} = \frac{\Delta p}{1-\Delta\overline{p}}$	critical belief
$u_{ll} \equiv U(u_{ub}, a_+, o_-)$	$u_{ll} = (1-2h) \cdot \frac{-\Delta p + (1-\Delta\overline{p})\ u_{ub}}{-\Delta p\ u_{ub} + (1-\Delta\overline{p})}$	belief loss upon losing
$u_{gw} \equiv U(0, a_+, o_+)$	$u_{gw} = (1-2h) \cdot \frac{\Delta p}{1+\Delta\overline{p}}$	belief gain upon winning
$u_{gl} \equiv U(0, a_-, o_-)$	$u_{gl} = (1-2h) \cdot \frac{\Delta p}{1-\Delta\overline{p}}$	belief gain upon losing

Table S7: Definitions of five special belief values.

The belief upper bound u_{ub} is a fixed point belief value that is reached upon successive winning; in the infinite horizon limit, the belief cannot exceed this value (it similarly cannot fall below $-u_{ub}$). If the belief initially begins above this value, successive wins will decrease the belief and drive it to this value.

The critical belief $u_{cr} = \Delta p/(1-\Delta\overline{p})$ specifies the value at which the optimal Bayesian agent switches from repeating the same action upon a loss (lose-stay) to switching actions upon a loss (lose-go):

$$o = o_- \rightarrow \begin{cases} l : \text{lose-go},\ a_t = \neg a_{t-1} & \text{if } u < u_{cr} \\ L : \text{lose-stay},\ a_t = a_{t-1} & \text{if } u > u_{cr} \end{cases} \tag{S17}$$

When $u_{cr} \rightarrow 0$, "lose-stay" will be a dominant action pattern. This occurs when a losing outcome provides little information about the current world state; i.e., when 1) reward gain is negative ($\Delta\overline{p} \ll 0$), so that losing is expected; or 2) reward contrast is low ($\Delta p \approx 0$), so that neither losing nor winning is informative.

Critical hazard rates. We define four critical hazard rates for which any two special belief values cross each other:

definition	solution	value
$u_{ll}(h) = u_{gw}(h)$	$h_{c0} = \frac{1}{2} + \frac{1-\Delta\overline{p}}{1+\Delta p^2-\Delta\overline{p}^2} - \frac{4}{\Delta p^2+(3-\Delta\overline{p})(1+\Delta\overline{p})}$	0.112
$u_{ll}(h) = u_{cr}(h)$	$h_{c1} = \ldots$	0.203 (analytical expression in Box S1 below)
$u_{gw}(h) = u_{cr}(h)$	$h_{c2} = \frac{-\Delta\overline{p}}{1-\Delta\overline{p}}$	$1/3$
$u_{ub}(h) = u_{cr}(h)$	$h_{c3} = \frac{1-\Delta\overline{p}}{4} - \frac{\Delta p^2}{4(1-\Delta\overline{p})}$	0.36

Table S8: Definitions of four critical hazard rates.

Box S1 | Analytical expression of h_{c1}

`h_c1 =`

$$(1/(12*(\mathtt{dpm}-1)^3)) * (2*(\mathtt{dpm}-1)*(\mathtt{dp}^2+3*(\mathtt{dpm}-1)^2) + 2^{1/3}*(\mathtt{dp}^6*(9*\mathtt{dpm}-5)*(\mathtt{dpm}-1)^3 - 18*\mathtt{dp}^4*(\mathtt{dpm}-1)^6 + 9*\mathtt{dp}^2*(\mathtt{dpm}-5)*(\mathtt{dpm}-1)^7 + 3*3^{1/2}*((\mathtt{dpm}-1)^8*(-(\mathtt{dp}^2-(\mathtt{dpm}-1)^2)^2)*(\mathtt{dp}^8*(2*\mathtt{dpm}-1) - 2*\mathtt{dp}^6*(\mathtt{dpm}-1)^2*(4*\mathtt{dpm}-3) + \mathtt{dp}^4*(\mathtt{dpm}-1)^2*(12*\mathtt{dpm}^3-23*\mathtt{dpm}^2+50*\mathtt{dpm}-23) - 8*\mathtt{dp}^2*(\mathtt{dpm}-2)*(\mathtt{dpm}-1)^4*(\mathtt{dpm}+1)^2 + 2*(\mathtt{dpm}-1)^6*(\mathtt{dpm}+1)^3))^{1/2})^{1/3)} + (2^{2/3}*(\mathtt{dp}^4*(3*\mathtt{dpm}-1)*(\mathtt{dpm}-1)^2 - 6*\mathtt{dp}^2*(\mathtt{dpm}-1)^5 + 3*(\mathtt{dpm}+1)*(\mathtt{dpm}-1)^6))/(\mathtt{dp}^6*(9*\mathtt{dpm}-5)*(\mathtt{dpm}-1)^3 - 18*\mathtt{dp}^4*(\mathtt{dpm}-1)^6 + 9*\mathtt{dp}^2*(\mathtt{dpm}-5)*(\mathtt{dpm}-1)^7 + 3*3^{1/2}*((\mathtt{dpm}-1)^8*(-(\mathtt{dp}^2-(\mathtt{dpm}-1)^2)^2)*(\mathtt{dp}^8*(2*\mathtt{dpm}-1) - 2*\mathtt{dp}^6*(\mathtt{dpm}-1)^2*(4*\mathtt{dpm}-3) + \mathtt{dp}^4*(\mathtt{dpm}-1)^2*(12*\mathtt{dpm}^3-23*\mathtt{dpm}^2+50*\mathtt{dpm}-23) - 8*\mathtt{dp}^2*(\mathtt{dpm}-2)*(\mathtt{dpm}-1)^4*(\mathtt{dpm}+1)^2 + 2*(\mathtt{dpm}-1)^6*(\mathtt{dpm}+1)^3))^{1/2})^{1/3}),$$

$$\texttt{where} \begin{cases} \Delta p \rightarrow \mathtt{dp} \\ \Delta \overline{p} \rightarrow \mathtt{dpm} \end{cases}$$

`This expression is acquired by using the Solve and Simplify functions in Mathematica [57]`

Box S2 | Minor numerical error as a result of a finite discretization

In Figure S17B-iii, we illustrate how a finite discretization can affect the convergence of the belief distribution near u_{ub}. This convergence can happen much faster with coarser bins, which results in an exaggerated probability at u_{ub} (instead of peaking at $u_{ub} - \epsilon$, with vanishingly small probability at u_{ub}).

In the next section, we will use the special belief values and critical hazard rates to analyze changes in the program space under variations in task parameters.

6 Sloppiness and the collapse of the good program space

6.1 Sloppiness as a property of task and strategy spaces

Previously, in Section 2, we discussed how task and functional sloppiness are related to the number of good strategies for solving the task. In this section, we take a closer look at how this number scales with 1) different task structures and 2) different constraints on the strategy space:

$$\text{sloppiness} \sim N_{\text{good strategies}}(\text{task}, \text{ constraints}) \tag{S18}$$

There are many different ways to formalize the dependence of sloppiness on the task and constraints. To formalize the task dependence, we compute the behavioral difference $d_{\text{opt-B, WSLG}}$ between the win-stay-lose-go (WSLG) program and an optimal Bayesian (opt-B) agent; the latter encodes the task structure in the belief update. To formalize the impact of constraints, we use discretized Bayesian programs (DBs) to study how many resources are required to approach the performance of the optimal Bayesian agent (quantified by the number of discrete states used to construct each DB; we consider DBs that have up to $M = 30$ states). This enables us to gain insight into the expected size of the good program space beyond $M = 5$ states; if more resources are required to approach a certain fixed performance threshold with respect to the optimal Bayesian agent, we expect a larger space of good programs. We then evaluate all programs (i.e., the entire space of good programs of size $M \leq 5$, and the set of DBs of size $M > 5$) on all tasks to see how well the good program space captures the optimal Bayesian behavior, and how much freedom (sloppiness) these good programs have in deviating from the optimal Bayesian behavior. Formally, we try to show:

$$\begin{aligned} \min M_{\text{DB}} &\sim 1/d_{\text{opt-B, WSLG}} \\ N_{\text{good programs}} &\sim d_{\text{opt-B, WSLG}} \cdot M \end{aligned} \tag{S19}$$

where $\min M_{\text{DB}}$ denotes the minimum number of states needed for a discretized Bayesian program to achieve a performance close to that of the optimal Bayesian agent. Note that the above formalization is meant to describe an approximate, rather than precise, relationship. Specifically, we would like to test the intuition that 1) when $d_{\text{opt-B, WSLG}}$ is small in a given task, many states are needed for a discretized Bayesian program to achieve optimal Bayesian performance, and 2) the size of the good program space grows with increasing $d_{\text{opt-B, WSLG}}$ or increasing M.

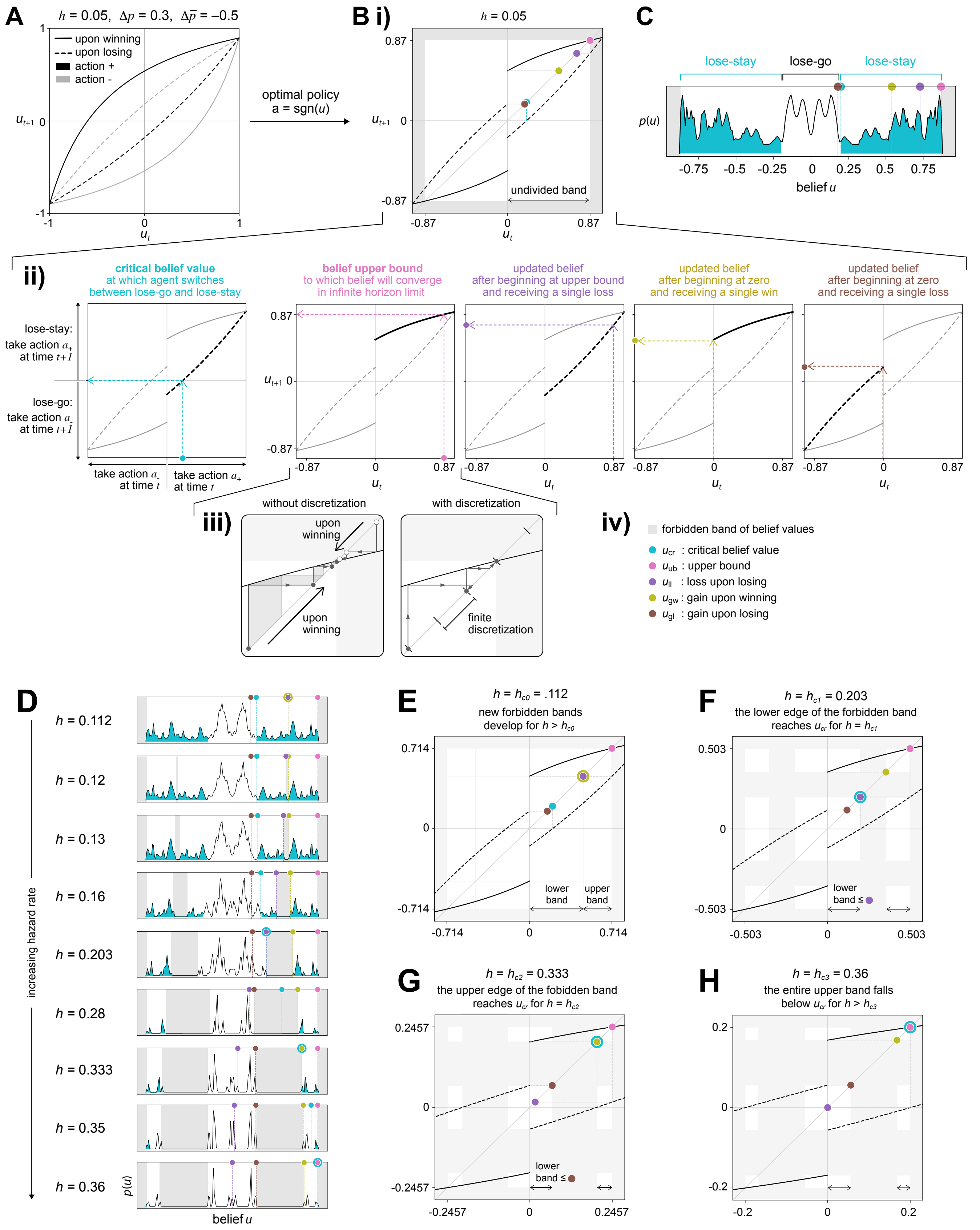

Figure S17: Fixed point analysis of optimal Bayesian agent. (A) Optimal belief update derived via Bayesian formalism. The four monotonic curves specify four different action-outcome pairs, respectively. **(B)** Belief update under the optimal behavioral policy. (i) The shaded regions mark forbidden belief values that cannot be attained given the structure of the task (i.e., the hazard rate, which specifies the volatility of the

Figure S17: (continued from previous page) environment, limits the maximum level of confidence that can be attained by an ideal Bayesian observer). Five colored dots mark five special belief values at a given hazard rate (see Table S7 for more detail). (ii) Explanation of each of the five special belief values. (iii) Discretizing the belief value can lead to an overestimation of the probability of reaching the belief upper bound (u_{ub}); see Box S2. (iv) Key denoting the five special belief values. **(C)** Infinite-horizon distribution of belief values. Turquoise shaded region depicts the total probability devoted to the lose-stay action-outcome contingency, as specified by the critical belief value. White region depicts the total probability devoted to the lose-go action-outcome contingency. Note that the belief distribution is normalized such that it sums to one; for visualization purposes, the vertical scale was chosen to highlight the shape of the distribution. **(D-H)** As the hazard rate changes (D), the relationship between the five belief values will also change, leading to changes in the forbidden belief values (gray bands in E-H) and the steady-state distribution of belief values (D). As in panel C, each belief distribution in panel D is normalized such that it sums to one, and vertical scales were chosen to highlight the shape of each distribution.

6.2 The many stages of behavioral convergence

Having defined the sets of special belief values and critical hazard rates in the previous section, we can examine how they impact the behavioral convergence between the optimal Bayesian agent (opt-B) and the win-stay-lose-go (WSLG) program as we increase the hazard rate from $h = 0.05$ to 0.5 (see Figure S17 for a graphical depiction). We observe the following behavioral changes as we sweep the hazard rate:

1. For small hazard rates, all five belief values are well spread out, and the optimal Bayesian agent can occupy a continuum of belief values that span a single continuous band (we will refer to these as the allowable belief values).
2. Above the first critical hazard rate h_{c0}, a forbidden band of belief values emerges and splits the allowable belief values into two separate bands (what we will refer to as 'upper' and 'lower' bands). The forbidden band emerges because only belief values above u_{ub} (which are not allowed) can transition into this band upon losing.
3. As the hazard rate increases, the forbidden band grows in size, and an increasingly large portion of the lower band falls below u_{cr}; this causes a gradual decrease in the probability of a lose-stay action (i.e., a decrease in $p\left(L\right)$)
4. Above the second critical hazard rate h_{c1}, the lower band falls completely below u_{cr}, causing a large reduction in $p(L)$.
5. As the hazard rate continues to increase, u_{cr} passes through the forbidden band, and the ratio $p\left(L\right)/p\left(l\right)$ (i.e., the relative probability of lose-stay vs lose-go actions) remains approximately constant.
6. Above the third critical hazard rate h_{c2}, u_{cr} enters the upper band, and the probability of a lose-stay action begins to decrease again.
7. Above the fourth critical hazard rate h_{c3}, the probability of a lose-stay action drops to zero, and the optimal Bayesian agent converges to the win-stay lose-go program.

The above transitions are summarized in the following table:
It is worth noting that the optimal Bayesian agent, with its reward-seeking policy, never switches actions upon winning; i.e., $p(w) = 0$, where w denotes the "win-go" action. A useful coarse-grained description for optimal Bayesian behavior can therefore be written as a vector of first-order sequence probabilities:

$$\mathbf{p}_{\text{opt-B}} \equiv \left(p_{\text{opt-B}}(W),\ p_{\text{opt-B}}(l),\ p_{\text{opt-B}}(L)\right) \tag{S20}$$

Similarly, a description of the WSLG program can be written as:

$$\mathbf{p}_{\text{WSLG}} \equiv \left(p_{\text{WSLG}}(W),\ p_{\text{WSLG}}(l)\right) \tag{S21}$$

We can use these descriptions to define the difference between the WSLG program and the optimal Bayesian agent:

task range	belief bands	band evolution
$h \in [0, h_{c0})$	$u \in [-u_{ub}, u_{ub}]$	single continuous band
$h \in [h_{c0}, h_{c1})$	$u \in [-u_{ub}, u_{ub}]$	onset of forbidden band at h_{c0} $\rightarrow$ forbidden band grows with increasing h
$h \in [h_{c1}, h_{c2})$	$u \in [-u_{ub}, -u_{ll}],\ [-u_{ll}, u_{ll}],\ [u_{ll}, u_{ub}]$ if $u_{ll} > u_{gl}$ $u \in [-u_{ub}, -u_{gl}],\ [-u_{gl}, u_{gl}],\ [u_{gl}, u_{ub}]$ if $u_{ll} < u_{gl}$	entire lower band falls below u_{cr} at h_{c1} $\rightarrow$ u_{cr} falls within forbidden band
$h \in [h_{c2}, h_{c3})$	$u \in [-u_{ub}, -u_{gl}],\ [-u_{gl}, u_{gl}],\ [u_{gl}, u_{ub}]$	upper band reaches u_{cr} at h_{c2} $\rightarrow$ upper band falls below u_{cr}
$h \in [h_{c3}, .5]$	$u \in [-u_{ub}, -u_{gl}],\ [-u_{gl}, u_{gl}],\ [u_{gl}, u_{ub}]$	entire upper band falls below u_{cr} at h_{c3}

Table S9: Evolution of allowed and forbidden belief values as a function of increasing hazard rate.

$$\begin{aligned} d^{-1}_{\text{opt-B, WSLG}} &\sim p\left(\text{WSLG} \mid \text{opt-B}\right) \\ &= \sum_{x \in \{W,l,L\}} \frac{p_{\text{opt-B}}(x) \times p_{\text{WSLG}}(x)}{p_{\text{opt-B}}(x) + p_{\text{WSLG}}(x)} \\ &\approx \frac{1}{2} - \frac{p_{\text{opt-B}}(L)}{4} - \frac{p^2_{\text{opt-B}}(L)}{8 p_{\text{WSLG}}(l)} \qquad \text{assuming } p_{\text{opt-B}}(l) \gg p_{\text{opt-B}}(L) \rightarrow 0 \end{aligned} \tag{S22}$$

Note that we choose instead to use the confusion probability (see Section 10.1 for a derivation of the confusion matrix) to capture the behavioral difference between WSLG and the Bayesian optimum:

$$p\left(\text{WSLG} \mid \text{opt-B}\right) \in [0, 0.5] \text{ where } \begin{cases} 0 & \text{if fully distinguishable} \\ 0.5 & \text{if opt-B and WSLG are identical} \end{cases}$$

In Figure S18D, we plot $p\left(\text{WSLG} \mid \text{opt-B}\right)$ as a function of hazard rate. One can see that the behavior of the optimal Bayesian agent converges to that of WSLG as $h \rightarrow h_{c3} = 0.36$. Crucially, before the convergence, the distinction between the two strategies is large if one uses longer behavioral sequences (length=10) to characterize them. When decreasing the hazard rate below h_{c3}, this behavioral difference rapidly increases without substantially changing performance. This implies a high degree of sloppiness, because there could be many other programs whose behavior and performance falls between these extremes. This point is discussed in more detail below.

6.3 Increasingly more resources are required to marginally improve performance at high hazard rates

The stiffness of the solution space near the last critical hazard rate, $h \approx h_{c3} = 0.36$, can be understood by estimating the amount of additional resources that are required to further improve performance beyond that of the WSLG program. To estimate this, we evaluate the performance of all 528 discretized Bayesian programs with sizes ranging from $M = 2$ to $M = 30$ states (note that the WSLG program is the smallest of these). These discretized Bayesian programs are found using two different discretization processes (see Box S3) across various hazard rates $h \in [0.05, 0.5]$. In Figure S18E-F, one can see that a 3-state discretized Bayesian program can improve performance beyond WSLG for low hazard rates ($h < h_{c2} = 0.33$). As the hazard rate increases above h_{c2}, this marginal improvement begins to decrease. For $h \geqslant 0.346$, discretized Bayesian programs with 3, 4, or even 5 states fail to exceed the performance of WSLG.

At first glance, it seems counterintuitive that making a smaller improvement at a higher hazard rate requires more resources than a larger improvement at a lower hazard rate. After all, a low-resource WSLG program seems to perform well in such a high hazard-rate task. To see why this is the case, consider the first-order behavioral difference between the optimal Bayesian agent and the WSLG program, i.e., $p_{\text{opt-B}}(L)$. At $h = 0.1$, the ratio between the probabilities of lose-stay vs lose-go actions is $p_{\text{opt-B}}(L)/p_{\text{opt-B}}(l) = 1.1$. For this ratio, one can sketch

a Bayesian-like program that has a similar proportion of lose-stay and lose-go edges (Figure S18G) and expect a large improvement over the performance of WSLG. In contrast, for $h = 0.35$, one has $p_{\text{opt-B}}(L)/p_{\text{opt-B}}(l) = 0.08$, which is a rather small adjustment from WSLG. To achieve such a skewed ratio, one needs to use more states to construct a Bayesian-like program in order achieve the right proportion of lose-go edges so as to further improve upon the WSLG program. In Figure S18F (dashed line), we see that a simple estimate of the number of states required for any improvement over WSLG roughly captures the results from our numerical experiment:

$$\min M_{\text{DB}} \sim \frac{p_{\text{opt-B}}(l)}{p_{\text{opt-B}}(L)} \tag{S23}$$

In our numerical experiment, no discretized Bayesian programs with more than 8 states (and fewer than 30 states) can improve performance over WSLG for $h \geqslant h_{c2}$.

It is interesting to note that the space of good programs can capture nearly all Bayesian-optimal behaviors, as illustrated in Figure S18K (compare "opt-B" vs "shared" curves); however, these deviate from one another when the hazard rate is either very low ($h < 0.1$) or very high ($h \approx 0.35$). A low hazard rate necessitates a long window of integration, and therefore requires a discretized Bayesian program with many states in order to approach the Bayesian optimum. As a result, a program space with $M \leqslant 5$ is not sufficient to capture all Bayesian behaviors. At the other extreme, a large hazard rate necessitates a very small percentage of lose-stay actions, which again requires a discretized Bayesian program with many states (and thus a much larger program space than the one considered here) in order to approach the Bayesian optimum.

Box S3 | Two discretization processes to generate a library of discrete Bayesian agents

In Figure S17D, we note that a band gap of forbidden belief values emerges at a hazard rate of $h = h_{c0}$. This forbidden band persists, and grows in width, until the hazard rate surpasses h_{c3}. It is therefore beneficial to construct a discretization scheme that does not waste any resources on these forbidden belief values. To achieve this, we removed the forbidden bands, concatenated the remaining belief values, and discretized them uniformly. We then combined these discretized agents with those generated from our earlier discretization scheme. We evaluated the ensemble of such agents in Figure S18E-F.

6.4 A sudden collapse of the good program space

In this section, we focus on a narrow range of hazard rates, $h \in [h_{c2}, h_{c3}]$, that display a phase transition in the behavior of the optimal Bayesian agent. In Figure S18D, we saw a sharp increase in the confusion probability $p\,(\text{WSLG} \mid \text{opt-B})$ once h exceeded h_{c2}. This is accompanied by a sudden collapse of the good program space—i.e., the WSLG program is the only remaining "good" strategy (see Figure S18H-I and Box S4):

$$N_{\text{good programs}} \approx 287 \text{ for } h = 0.33$$
$$N_{\text{good programs}} \to 1 \quad \text{for } h \to 0.36$$

It is interesting to note that there is a very narrow plateau in both $p\,(\text{WSLG} \mid \text{opt-B})$ and $N_{\text{good programs}}$ for $h \approx 0.35$. For variations about this hazard rate, the optimal Bayesian behavior does not change much, nor does the size of the good program space. This can be understood by examining how the belief distribution evolves from $h = 0.33 \to 0.35 \to 0.36$ in Figure S17D.

By relating our fixed point analysis of the optimal Bayesian agent to the changing size of the good program space, we have demonstrated that 1) the simple analytic Bayesian belief update does not translate into "simple" Bayesian behavior (see, e.g., the complexity of the belief distributions in Figure S17D); and 2) smooth changes in task structure (e.g., hazard rate) and performance do not necessarily translate into smooth changes in the behavior of the optimal Bayesian agent or in the size of the good program space.

Box S4 | A space of good programs that is shared across different hazard rates implies shared computations

In order to compute the size of the good program space shown in Figure S18H-I, one does not need to re-evaluate the full program space for changes in hazard rate; this is because the good program space at high hazard rates is contained within the good program space at low hazard rates (up to small numerical errors due to the finite discretization of belief values; see Box S2). We verified this through a full enumeration at three hazard rates: $h \in \{0.15,\ 0.25,\ 0.35\}$. Given this observation, one can in principle re-evaluate the 4,230 good programs at $h_{\text{min}} = 0.05$ for all higher hazard rates. To be on the safe side, we relaxed our criterion level of performance, so as to include programs whose performance exceed 99% of that achieved by WSLG. This expands the space of good programs from 4,230 to 6,961. After evaluating these 6,961 programs at different hazard rates, we found that there are only 3 programs that were not in the original 4,230 good programs, but whose performance exceeds that of WSLG. This consistency of the good program space implies that the task is qualitatively similar despite changes in hazard rate. It is therefore important to move away from a simple parameterized task if we wish to study generalizability in a task space where the sets of good strategies for each task overlap in a more complex way.

6.5 The number of permissible behaviors explodes with small deviations from the optimal Bayesian agent

To close this section, we examine the origin of this sudden collapse of the good program space. More intuitively, we view this collapse as an explosion in reverse. In Figure S18H-I, we see that the sequences produced by the optimal Bayesian agent completely overlap with those produced by the WSLG program if $h \geqslant 0.36 + \epsilon$ (here, $\epsilon \neq 0$ because of the discretization errors discussed in Box S2). As one decreases the hazard rate below $h \lesssim 0.36$, the optimal Bayesian agent produces an increasingly large number of sequences that show a decreasingly small overlap with the WSLG program. The right panel of Figure S18J illustrates how this can happen, even with a small change in the frequency of the lowest order behavioral statistics:

$$\frac{p_{\text{opt-B}}(L)}{p_{\text{opt-B}}(l)} = \quad 0 \quad \text{for } h \approx 0.36$$
$$\frac{p_{\text{opt-B}}(L)}{p_{\text{opt-B}}(l)} \to 0.04 \quad \text{for } h \lesssim 0.36$$

Although the proportion of lose-stay (L) actions is small, it generates a large number of new behavioral sequences when combined with the original two actions, win-stay and lose-go ($W,\ l$). This kind of sloppiness also exists within the space non-Bayesian behavior. In Figure S18K, one can see that 1) the good program encompasses nearly all optimal Bayesian sequences; and 2) there are many more sequences beyond what are produced by the optimal Bayesian agent. The text panel on the right of Figure S18K shows how a small proportion of win-go actions (actions than an optimal Bayesian agent would never take) creates a large number of new sequences.

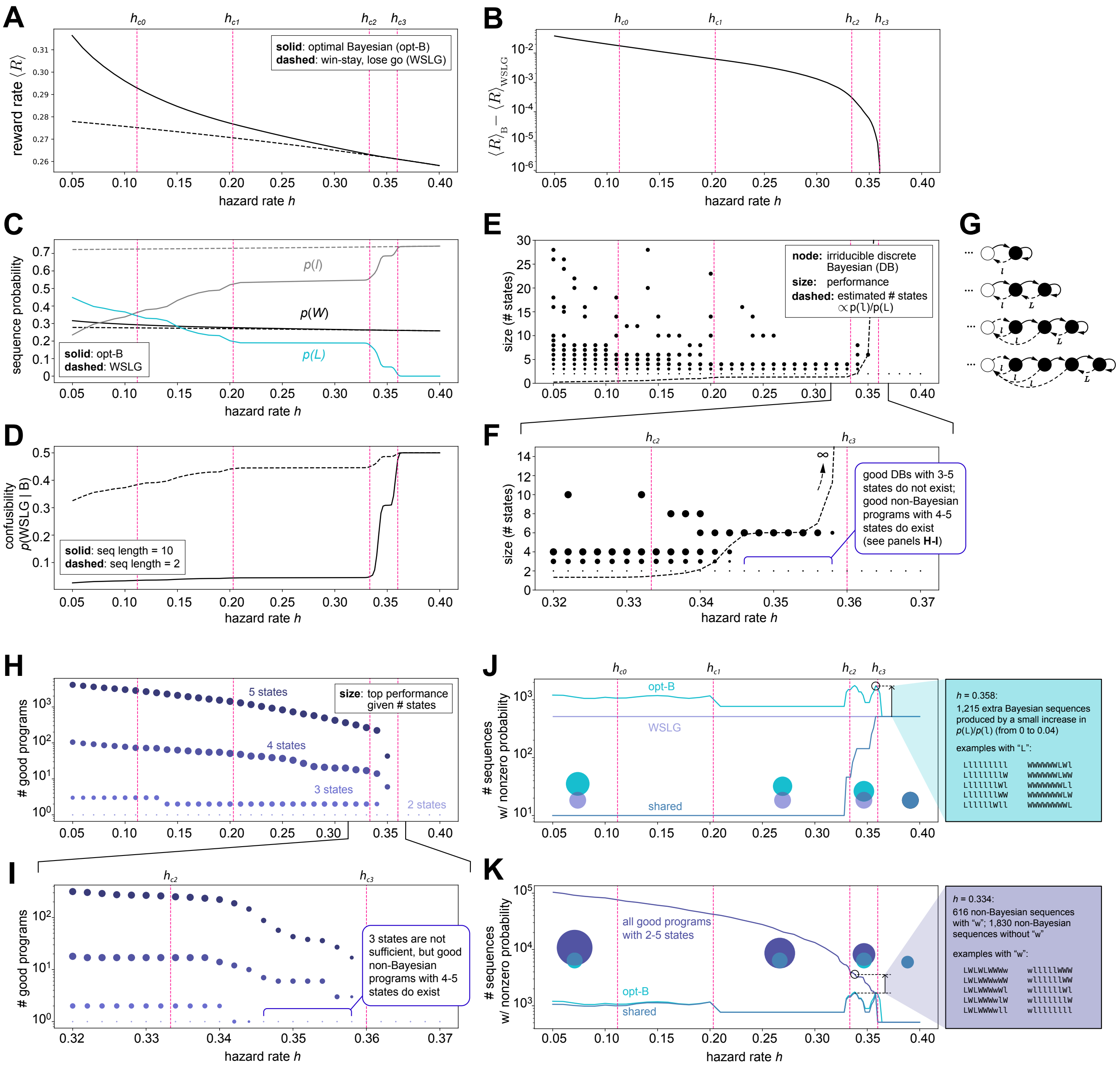

Figure S18: Task-related sloppiness emerges from the structure of the task and the constraints on the program space. **(A)** As hazard rate increases, the performance of the optimal Bayesian (opt-B) agent converges to that of the WSLG program. **(B)** The difference in performance between the optimal Bayesian agent and the WSLG program rapidly drops to zero as $h \rightarrow h_{c3}$. Note that this curve is smooth, and does not show abrupt changes in behavior. **(C)** Statistics of first-order sequences produced by the optimal Bayesian agent and the WSLG program as a function of hazard rate. Note that the optimal Bayesian agent shows step-like changes in these lowest-order behavioral sequences, and specifically in the prevalence of lose-stay versus lose-go sequences (i.e., $p(L)$ versus $p(l)$). **(D)** The probability of confusing the optimal Bayesian agent with the WSLG program, computed with short (length = 2) and long (length = 10) behavioral sequences. For long behavioral sequences, the optimal Bayesian agent undergoes abrupt transitions near h_{c2} and h_{c3} (consistent with the results shown in panel C). **(E-F)** At each hazard rate, we mark each discretized Bayesian (DB) program that can incrementally improve performance over the closest smaller DB program. As the hazard rate increases, it becomes increasingly more difficult to find a DB program that can improve performance with the addition of a small number of states (see blue inset in panel F). The dashed line marks an estimate of the number of states needed to improve performance over the WSLG program, estimated by tracking the ratio $p(L)/p(l)$ that a DB program has to achieve. **(G)** Schematic illustrating how a larger DB program is better able to achieve a skewed ratio of $p(L)/p(l)$. **(H-I)** The good program space begins to collapse as the hazard rate increases (see blue inset in panel I, which highlights the collapse of 3-state programs). The size of the good program space—and hence the degree of sloppiness in the set of strategies that can achieve good performance—closely tracks the changing behavior of the optimal Bayesian agent (see panel C). **(J)** Small changes in $p(L)/p(l)$ (induced by decreasing the hazard rate below h_{c3}) enable a combinatorial increase in the number of permissible behavioral sequences, which in turn leads to rapid deviations between the optimal Bayesian agent and the WSLG program. Circle diagrams illustrate the overlap between the sequences generated by the optimal Bayesian agent and the WSLG program (which corresponds to the number of shared sequences; blue curve). Turquoise box: illustration of the new sequences that emerge following a small increase in $p(L)/p(l)$. **(K)** Analogous to panel J, but now comparing the optimal Bayesian agent with the ensemble of good programs. Purple box: illustration of the new sequences that emerge following a small increase in $p(w)$.

7 Program enumeration

A key challenge of enumeration is identifying relevant constraints that can be used to make the enumeration manageable. For example, one might need to store all of the enumerated quantities within the available memory on a computer cluster, or one might require the enumeration to be complete within a matter of hours, rather than days. The constraints that enable this efficiency can be broadly categorized into two groups:

1. *parametric constraints*; e.g., *resource constraints* that specify the number of program states
2. *non-parametric constraints*; e.g., *rule-out rules* that eliminate unwanted programs (for example, a large programs that behaves identically to a smaller program)

In this study, we imposed a maximum resource constraint of 5 program states, and enumerated programs subject to this constraint. Within this enumeration, we imposed a set of rule-out rules to remove invalid and redundant programs. In what follows, we discuss the steps of this enumeration in detail.

7.1 Multiple tables represent a single program

We construct behavioral programs as deterministic Markov chains, which are graphs without any inherent notion of node ordering. However, to enumerate a graph, one has to embed it with some choice of node labeling. This is partly why the full program enumeration requires rule-out rules to eliminate programs that appear distinct (based on the choice of representation) but generate identical behavior. Below is an example that highlights two different representations of the same program:

	m	$m' \mid$ losing	$m' \mid$ winning	m	$m' \mid$ losing	$m' \mid$ winning
tabular representation	$a_- \leftarrow 0$	1	1	$a_+ \leftarrow 0$	1	0
	$a_+ \leftarrow 1$	0	1	$a_- \leftarrow 1$	0	0
		$\Downarrow$			$\Downarrow$	
program tuple: (`outmap, inmap`)	$\left((a_-, a_+), \begin{pmatrix} 1 & 1 \\ 0 & 1 \end{pmatrix}\right)$			$\left((a_+, a_-), \begin{pmatrix} 1 & 0 \\ 0 & 0 \end{pmatrix}\right)$		

Table S10: Two distinct representations of the same program.

Note that each M-state program can be represented as a tuple with an M-dimensional "`outmap`" vector with binary action labels and a $M \times 2$ "`inmap`" array with state labels $m \in [0, M-1]$. Below we list two types of rule-out rules that operate at the level of program tuples and remove invalid tuples. What remains is then a complete set of unique programs.

7.2 Rule-out rules

The first set of three rule-out rules are constructed based on generic features of Markov chains, and are thus task-independent. These three rules filter out Markov chains that are ill-behaved or that are identical under node permutation. The second set of rule-out rules eliminate programs that are equivalent under symmetries or structures present in our task, and are thus task-dependent. These rules are summarized in Table S11 and Table S12:

rule	note
Rule 1) Remove identical graphs under node permutation	The most obvious rule-out rule recognizes that the underlying graph structure of a program is unchanged regardless of how one indexes the nodes to construct the corresponding tabular representation.
Rule 2) Remove reducible Markov chains	Rules 2 & 3 retain only well-behaved Markov chains. A reducible Markov chain contains sinks—a subset of nodes that absorbs all occupancy during random walk, and drains—the subset of remaining nodes.
Rule 3) Remove periodic Markov chains	A periodic Markov chain results in a non-converging distribution of node occupancy. Rules 2 & 3 can be checked using an open python library: QuantEcon [52]

Table S11: Task-independent rule-out rules.

rule	note
Rule 4) Remove identical graphs under node inversion	Since our task is symmetric to the exchange of actions, flipping the policy ($a_\pm \to a_\mp$) would neither change the behavior nor the performance.
Rule 5) Remove programs that contain mergers	Mergers are sets of nodes that can be combined without changing a program's behavior. In the section below, we show how this rule can detect all types of mergers.

Table S12: Task-dependent rule-out rules.

7.3 Algorithmic steps to rule out a merger program

Below, we detail the steps involved in applying Rule 5 to identify merger programs. We focus on this rule because 1) it is hard to intuit, and 2) it provides a way to measure the structural distance between two programs of different sizes (we will return to this in Section 9.1). This rule involves first enumerating all possible ways to merge multiple groups of nodes, and then checking to see if the resulting merged program produces identical behavior as the original program. The first step is thus to identify all unique node partitions for a given program.

Step 1) determine all possible partitions of M program states (nodes). For a given number of states, say $M = 5$, one can create either 1, 2, 3, or 4 groupings over nodes. We refer to these groupings as "partition types":

# groups	possible partition types
1	`(5,)`
2	`(4,1), (3,2)`
3	`(3,1,1), (2,2,1)`
4	`(2,1,1,1)`

For a particular partition type, one can generate all possible groupings, or partitions, of node IDs; we then remove all repeated groupings. For example, the following two partitions are identical:

$$\text{partition type} = (2,2,1) \to \text{partitions} : \begin{cases} ((0,1),\ (2,3),\ (4,)) \\ ((2,3),\ (0,1),\ (4,)) \end{cases}$$

Step 2) remove partitions that contain different actions within any group. If a group within a partition contains multiple nodes that are not labeled by the same action, these node cannot be merged. For example, given an `outmap=(-,+,+,+,+)`, a `partition=((0,1), (2,3), (4,))` can be relabeled as `((-,+), (+,+), (+,))` . Since the first group contains different actions, this partition does not constitute a valid merger.

Step 3) relabel node IDs with group IDs, and reduce the `inmap` accordingly. In this step, we create a mapping from the original node IDs, say `m=(1,2,3,4,5)`, to a smaller set of group IDs, say `g=(A,B,C)`. For example,

$$((2,3,4),\ (0,),\ (1,)) \rightarrow \texttt{map} = \{\texttt{2 : A},\ \texttt{3 : A},\ \texttt{4 : A},\ \texttt{0 : B},\ \texttt{1 : C}\}$$

We then replace the node IDs with the group IDs within the `inmap`, in order to create a reduced map `inmap_r`:

$$\texttt{inmap} = \begin{pmatrix} 1 & 0 \\ 0 & 2 \\ 1 & 3 \\ 1 & 4 \\ 1 & 2 \end{pmatrix} \xrightarrow{\texttt{map}} \texttt{inmap\_r} = \begin{pmatrix} C & B \\ B & A \\ C & A \\ C & A \\ C & A \end{pmatrix} \tag{S24}$$

Step 4) check if each group in the `inmap` has consistent winning & losing transitions. From the above example, one can see that for group A (i.e., group `(2,3,4)`, corresponding to rows 3-5 of `inmap_r`), the first column (corresponding to a losing outcome) contains only a single transition to C; similarly, the second column (corresponding to a winning outcome) contains only a transition to `A`:

$$\texttt{inmap\_r} = \begin{pmatrix} C & B & \\ B & A & \\ C & A & \leftarrow 2 \\ C & A & \leftarrow 3 \\ C & A & \leftarrow 4 \end{pmatrix} \tag{S25}$$

The consistency of transitions within a group confirms that this partition creates a valid a merger program, and thus the original program can be reduced to a smaller program under this particular partition.

7.4 Optimizing algorithmic steps for a full enumeration.

Having specified the set of rule-out rules, one can combine them in the most efficient order for optimizing the speed of enumeration, the cost of memory, etc., on a given computer cluster. Below, we detail the steps of our particular optimization:

Step 1) enumerate all possible `outmaps` with standardized ordering. If two tabular representations coincide after a given node permutation, they have the same `outmap`. Given this, one can fix the `outmap` to be of the following vectors:

```
standard_outmap_set = {(-,+), (-,+,+), (-,+,+,+), (-,-,+,+), (-,+,+,+,+), (-,-,+,+,+)}
```

Note that this set is constructed to have a greater or equal number of actions a_+ compared to a_-, and gathers all actions a_- to the left side.

Step 2) enumerate `inmaps`. For a given program size M, there are M^{2M} different `inmaps`. Each `inmap` can then be paired with all compatible `outmaps` to generate all possible program tuples: `program=(outmap, inmap)`. The total number of program tuples scales with M as follows:

$$N_{\text{tuples}} = 2^M \times M^{2M} \tag{S26}$$

Step 3) remove `inmaps` with sinks and drains. Because this step is the fastest and does not involve the `outmap`, we use it as the first filter to eliminate a majority of invalid programs.

Step 4) remove mergers. For each program tuple `program`, we use Rule 5 (discussed above) to eliminate all merger programs.

Step 5) remove repeated programs. This step requires comparing a given program and all other valid programs in an ensemble, and is thus a much step than the previous steps. We first collect all existing program tuples `(outmap, inmap)`. For a given tuple, we generate all permuted versions that do not change the `outmap` (i.e., node permutations that would output the same or mirror versions of the original `outmap`). We then check if any of these permuted tuples already exist within the ensemble of valid program. If not, we append this tuple to the list of valid programs.

Step 6) remove reducible and periodic `inmaps`. Checking these features of Markov chain with QuantEcon is slow compared to the previous steps, and removes far fewer programs. Because of this, we apply this rule last.

With the above rules, we can enumerate up to $M = 5$ with ease; this becomes difficult (but still doable) for $M = 6$, and impossible for $M = 7$. Below, we summarize the size dependence of this enumeration:

M	# unique tuples	# unique programs
2	64	5
3	5,832	124
4	1,048,576	4,979
5	312,500,000	263,428

Table S13: Number of unique programs for increasing program sizes.

From this result, one can see that full enumeration becomes infeasible as M increases. It is therefore important to constrain the enumeration either through an evolutionary algorithm (discussed in Section 9.4 below) or by leveraging other features that could be beneficial for solving this task (see discussion in Section 4.2).

8 Program evaluation

8.1 Belief Distribution Propagation (BDP)

The most common way to evaluate a Markov model is to use Monte Carlo simulation, in which one needs a long trajectory in order for any given statistic (e.g., the state occupancy) to converge. In our case, we can bypass this issue by propagating an entire belief distribution over time (what we refer to as “Belief Distribution Propagation”, or BDP). The convergence of BDP is orders-of-magnitude faster than that obtained via Monte Carlo simulation, but it comes at the cost of needing to store the full distribution in memory, rather than needing to store a single belief value.

BDP for the optimal Bayesian agent. To evaluate the optimal Bayesian agent, we begin by initializing the belief distribution to be uniform across belief values $u \in [-1, 1]$, discretized into 200 bins. In the first iteration at $t = 0$, we propagate the probability of each belief value based on all four action-outcome pairs; this then generates four new belief values at $t = 1$ with corresponding probabilities:

$$p(u_{t+1}, a_t, o_t) = p(u_t) \times \underbrace{p(o_t \mid u_t, a_t)}_{\text{outcome probability}} \; \underbrace{\delta(u_{t+1} - U(u_t, a_t, o_t))}_{\text{Bayesian inference}} \; \underbrace{\pi(a_t \mid u_t)}_{\text{optimal policy}} \tag{S27}$$

where $U(u, a, o)$ is the belief update derived in Equation (S3), $p(o \mid u, a)$ is the outcome probability derived in Equation (S8), and $\pi(a \mid u)$ is the optimal policy derived in Equation (S10). The updated belief distribution $p(u_{t+1})$ is obtained by summing over all action-outcome pairs and integrating over the previous belief distribution $p(u_t)$:

$$
\begin{aligned}
p\left(u_{t+1}\right) &= \int du_{t+1} \sum_{a,o} p\left(u_{t+1}, a_t, o_t\right) \\
&= \int du_{t+1}\, p\left(u_t\right) \times \sum_{a_t,o_t} p\left(o_t \mid u_t, a_t\right)\, \delta\left(u_{t+1} - U\left(u_t, a_t, o_t\right)\right)\, \pi\left(a_t \mid u_t\right)
\end{aligned} \tag{S28}
$$

BDP for a small program. BDP for small program follows the same logic as derived above; the only extension is to construct a joint distribution over both program states and belief states. The update rule is therefore:

$$
\begin{aligned}
p(m_{t+1}, s_{t+1}) &= \sum_{a,o} p(m_{t+1}, s_{t+1}, a_t, o_t) \\
&= \sum_{m_t,s_t} p(m_t, s_t)\, p(s_{t+1} \mid s_t) \times \sum_{a_t,o_t} p(o_t \mid s_t, a_t)\, \delta(m_{t+1} - V(m_t, a_t, o_t))\, \pi(a_t \mid m_t)
\end{aligned} \tag{S29}
$$

where m is a state within a specific program, and V and π denote the corresponding state update rule and policy specified as a deterministic Markov chain (see Section 7 for details). Note that the output of BDP is equivalent to finding the leading eigenvector (i.e., the belief distribution) of a joint transition matrix of a given Markov chain. The eigenvector approach is faster than BDP, but it is harder to scale up to larger joint distributions, as we discuss in Section 8.3 below when trying to propagate distributions of behavioral sequences.

8.2 From belief distribution to performance

Having found the converged belief distribution $p\left(u\right)$ or $p\left(u, m\right)$ for a given agent, it is straightforward to compute the corresponding reward rate:

$$
\begin{aligned}
\langle R \rangle &= \int du\, p(u) \sum_{a} p(o_+ \mid u, a)\, \pi(a \mid u) && \text{Bayesian agent} \\
\langle R \rangle &= \sum_{m,s} p(m, s) \sum_{a} p(o_+ \mid s, a)\, \pi(a \mid m) && \text{for a small program}
\end{aligned} \tag{S30}
$$

8.3 Sequence Distribution Propagation (SDP)

The goal of SDP is to iteratively build a distribution of behavioral sequences by updating a belief distribution while simultaneously increasing its depth. Unlike BDP, which only retains the distribution from the previous time step, SDP keeps up to l-steps in the past, to correctly generate a distribution over sequences of length l.

Two auxiliary transition matrices. To simplify the analytical expression for the update rules, we defined two auxiliary transition matrices, A and B:

$$
\begin{aligned}
A &\equiv p(a_t, o_t \mid m_t, s_t) && \text{action-outcome from belief} \\
&= \underbrace{p(o_t \mid s_t, a_t)}_{\text{reward delivery}}\ \underbrace{\pi(a_t \mid m_t)}_{\text{policy from prog } i} \\
B &\equiv p(m_t, s_t \mid m_{t-1}, s_{t-1}, a_{t-1}, o_{t-1}) && \text{joint belief-state update} \\
&= \underbrace{p(s_t \mid s_{t-1})}_{\text{world dynamics}}\ \underbrace{p(m_t \mid m_{t-1}, o_{t-1})}_{\text{inmap of prog } i}
\end{aligned} \tag{S31}
$$

We can then use these matrices to initialize the $l = 1$ sequence distribution, Y_0, in terms of the prior X_0 obtained from BDP:

$$
\begin{aligned}
X_0 &\equiv p(m_0, s_0) \\
Y_0 &\equiv p(a_0, o_0) \\
&= \sum_{m_0, s_0} p(a_0, o_0 \mid m_0, s_0)\, p(m_0, s_0) \\
&= AX_0
\end{aligned}
\tag{S32}
$$

Iterative update rules. The belief and sequence distributions at time t (X_t and Y_t, respectively) can now be iteratively defined in terms of the previous quantities:

$$
\begin{aligned}
X_t &\equiv p(m_t, s_t \mid a_{t-1}, o_{t-1}, \ldots, a_0, o_0) \\
&= \sum_{m_{t-1}, s_{t-1}} \underbrace{p(m_t, s_t \mid m_{t-1}, s_{t-1}, a_{t-1}, o_{t-1}, \ldots)}_{B}\; p(m_{t-1}, s_{t-1} \mid a_{t-1}, o_{t-1}, \ldots) \\
&= \sum_{m_{t-1}, s_{t-1}} B \overset{1}{-}\; \underbrace{p\left(a_{t-1}, o_{t-1} \mid m_{t-1}, s_{t-1}, \cancel{a_{t-2}}, \cancel{o_{t-2}} \ldots\right)}_{A}\; \underbrace{p(m_{t-1}, s_{t-1} \mid a_{t-2}, o_{t-2} \ldots)}_{X_{t-1}} \\
&= BAX_{t-1} \\
\\
Y_t &\equiv p(a_t, o_t, a_{t-1}, o_{t-1}, \ldots, a_0, o_0) \\
&= p(a_t, o_t \mid a_{t-1}, o_{t-1}, \ldots, a_0, o_0)\; \underbrace{p(a_{t-1}, o_{t-1}, \ldots, a_0, o_0)}_{Y_{t-1}} \\
&= \sum_{m_t, s_t} \underbrace{p\left(a_t, o_t \mid m_t, s_t, \cancel{a_{t-1}}, \cancel{o_{t-1}}, \ldots\right)}_{A}\; \underbrace{p(m_t, s_t \mid a_{t-1}, o_{t-1}, \ldots)}_{X_t}\; Y_{t-1} \\
&= AX_t Y_{t-1}
\end{aligned}
\tag{S33}
$$

SDP for the optimal Bayesian agent To compute the distribution of sequences produced by the optimal Bayesian agent, we apply similar update rules as derived above:

$$
\begin{aligned}
X_t &\leftarrow BAX_{t-1} \\
Y_t &\leftarrow AX_t Y_{t-1}
\end{aligned}
\tag{S34}
$$

where $A = p\left(o_t \mid u_t, a_t\right) \pi\left(a_t \mid u_t\right)$, $B = p\left(u_t \mid u_{t-1}, a_{t-1}, o_{t-1}\right)$, $X_0 \equiv p\left(u_0\right)$, and $Y_0 = AX_0$.

In this study, we propagate a distribution of sequences up to a maximum length of $l_{\text{max}} = 10$, which generates a distribution of $4^{10} = 1,048,576$ distinct sequences.

9 Extracting structural relationships within the program space

9.1 Measuring structural distances

When two programs have the same size. It is relatively straightforward to define the structural distance between two programs of the same size. One simply chooses one of the two programs to have a fixed tabular representation (i.e., a fixed node ordering), and permutes the nodes of the other program until the difference between the two tabular representations is minimized (node that this permutation includes flipping the action labels of all nodes, since this task is symmetric to the exchange $a_{\pm} \rightarrow a_{\mp}$). Here, we compute binary differences d_k between all entries of the tabular representation:

$$
\begin{aligned}
d_k\left(\text{prog } i, \text{prog } j\right) &= \begin{cases} 0 & \text{if entry } k \text{ is identical for both programs} \\ 1 & \text{otherwise} \end{cases} \\
d\left(\text{prog } i, \text{prog } j\right) &\equiv \sum_k d_k\left(\text{prog } i, \text{prog } j\right)
\end{aligned} \tag{S35}
$$

When two programs differ in size. Having previously defined equivalent "merger" programs (see the description of Rule 5 in Section 7.3 for more detail), it is now straightforward to define the structural distance between two programs that differ in size. Consider two programs, program A and B of sizes M and $M + \Delta M$, respectively. To measure the distance between A and B, one can first enumerate all valid merger programs of size $M + \Delta M$ that are equivalent to program A, and then find the merger program that minimizes the structural distance to program B.

9.2 Extracting minimal relationships via a Tree Embedding (TE)

In the main text, we constructed a tree embedding (TE) with the goal of capturing a minimal yet informative set of relationships within the space of programs. With this goal in mind, we chose to embed the space using a tree structure, rather than another graph structure, because of the minimal number of edges it contains. We constructed this tree embedding in a manner that prioritizes smaller programs closer to the root of the tree. This allowed us to capture the functional diversity that emerges toward the leaves of the tree, in programs with larger numbers of states.

The TE algorithm consists of two steps (illustrated in Figure S4A): 1) construct a list of programs, sorted first according to increasing program size, and next according to decreasing performance; and 2) for each program in the list, find one parent from the top of the list that is within a single mutation. The first step sorts programs according to size and performance; this sorting ensures that all five unique two-state programs are root nodes of the tree, and that most of the larger programs are closer to the leaf nodes. In the second step, one loops through the list of all programs (program i in Figure S4A) and searches for the first program j on the same list that is within a single mutation. Because of the sorting applied in step 1, this algorithm preferentially connects a child program i to a parent program j that is relatively small in size and high in performance.

9.3 A randomized Tree Embedding predicts the robustness of evolutionary algorithms

Using the standard TE described above, we discovered that nearly all good programs are closely connected through single mutations (see Figure 2 in the main text, and Figure S5). One might question whether this observation is simply an artifact of the secondary performance-based sorting that we applied above. To address this, we randomized the performance of all programs in the list, and we repeated the same embedding. The result is shown in Figure S8. We find that the results are largely unchanged, and the program space retains a highly-structured relationship between program structure and performance. This finding predicts that an evolutionary algorithm should be successful in iteratively discovering good programs through single mutations, something that discuss in more detail in the following section.

9.4 Efficient search via an evolutionary algorithm

In Section 7, we described the steps needed to enumerate a complete ensemble of unique programs. Here, we describe an evolutionary algorithm that can be used to recover a large fraction of good programs by searching a relatively small fraction of this space. This algorithm could enable us to extend this work to larger program spaces, where full enumeration is not possible.

Our evolutionary algorithm has three key features: 1) it considers all possible mutations of a given program at each generation (rather than doing random mutations); 2) it uses a variable threshold to select mutated programs; and 3) it hibernates unselected mutants to allow for selection at a later time. These features can be implemented with a simple set of operations, as illustrated in Figure S9A and described below.

Operations within the evolutionary algorithm. The local enumeration operates on a single data frame that we refer to as the `program_reservoir`. This data frame is a spreadsheet in which each row stores all relevant attributes for a unique program. Below is an example:

prog id	parent prog id	prog size	reward rate	t_born	partition_born	t_morph	partition_morph	status
3	None	2	.263867	0	None	[1]	[(1,1), (2,1)]	morph
4	None	2	.277911	0	None	[ ]	[ ]	idle
...	...	...	...	...	...	...	...	...

To grow this list, we start from a small set of valid programs, and we use the following four operations in sequence at each epoch. Each operation takes in a set of individual programs from the current list, and updates the `program_reservoir` according to a set of specific rules. Although these rules can be arbitrarily complex based on program attributes, we aimed to finding the simplest set of effective rules.

`morph_op`. This operation closely resembles the random mutation step in an evolutionary algorithm. The only difference is that in our case, we enumerate every possible mutation without any randomness or sub-sampling. Below is the pseudocode:

1. Grab all programs with status "`morph`."
2. Assign the current epoch "`t_morph`" to the selected programs.
3. Assign the two simplest partition types, i.e., `partition_morph=[(1,1,...,1), (2,1,...,1)]`, to individual programs of a given size M. Note that first partition type will generate all mutants of the same size M, while the second partition type will generate all mutants of size $M+1$ (these are generated across all possible groupings of two nodes within the partition type; see Section 7.3 for more details regarding partitions).
4. Based on `partition_morph`, generate mutants of the appropriate size (these mutants comprise the set of all programs of greater or equal size that are within a single mutation).
5. Filter invalid mutants with the standard rule-out rules (see Table S11 and Table S12 for details).
6. Output a list of mutants with the attributes "`t_born`" (the epoch at which the mutant was generated) and "`partition_born`" (the corresponding `partition_morph`).
7. Change the status of all programs with label "`morph`" to "`frozen`".

`append_op`. This operation checks whether a new mutant is already within the `program_reservoir`. To perform this operation, we can reuse two rule-out rules from the full enumeration:

1. Remove identical graphs under node inversion.
2. Remove identical graphs under node permutation.
3. Assign a status of "`idle`" to newly appended programs.

`eval_op`. This operation determines the reward rate for each program by searching the database generated from the full enumeration. For $M \geq 6$ where such a database doesn't exist, one can run BDP (see Section 8.1) to evaluate the reward rate.

`select_op`. This operation determines the fraction of mutated programs that will be selected in each epoch. This is a crucial step in our local enumeration; depending on the number and reward rates of the selected programs, this evolutionary algorithm will explore different regions of the program space with different rates. We choose the simplest selection rule that allows us to cover a large fraction of good programs identified through a minimal exploration of the program space:

1. Grab all programs with status "`idle`."
2. Compute $\texttt{num\_morph} = \texttt{log}_2(\texttt{num\_idle})/2$
3. Change the status of the top `num_morph` performing programs from "`idle`" to "`morph`"; leave the status of the remaining programs as "`idle`".

This specific selection rule has several advantages. First, it is equivalent to flexibly adjusting the reward threshold for selecting good programs. There could be a particular epoch where new mutants don't perform well. Because this rule selects `~log(num_idle)` programs, such a local barrier won't hinder the local enumeration. Second, by selecting `~log(num_idle)` programs, the resulting enumeration grows at a polynomial rate, rather than an exponential one, thereby allowing us to extend this algorithm to larger numbers of epochs. Third, this selection rule does not discard any program within the `program_reservoir`; rather, it hibernates unselected programs to be selected in later epochs.

Results. From Figure 2E in the main text, and from Figure S9B, one can see that the evolutionary algorithm efficiently finds a majority of good programs after searching only a fraction of the full program space. Interestingly, in Figure S9B, one can see that the larger tree is actually rooted around the second highest performing two-state program, instead of the highest performing (WSLG) two-state program. This highlights why it can be advantageous to retain programs with slightly lower performance during the evolutionary search.

9.5 Visualizing the full program space with a reduced Tree Embedding (rTE)

In Figure 2 in the main text, we directly visualized a smaller program space ($M \leqslant 4$, with 5,108 programs) using Gephi [53]. However, visualizing the full space with $M \geqslant 5$, which consists of 268,536 programs, is not doable in Gephi. We therefore constructed a reduced Tree Embedding that groups neighboring programs based on their local and global relationships (the results are shown in Figure S5).

Local relationships between programs. As discussed in Section 9.1, two nearby programs with the same or different sizes can be categorized by a partition of their corresponding merger program. For example:

size change upon mutation	all possible partition types
$M = 3 \to 3$	(1, 1, 1)
$M = 3 \to 4$	(2, 1, 1)
$M = 2 \to 4$	(3, 1), (2, 2)

The partition type is therefore a way to measure whether two descendants are structurally similar. We consider two programs to be similar if they share the same "merger structure", or if they share the same partition type after mutating from their parent.

Global relationships between programs. We use two global relationships to construct our rTE. The first is a "`branch`", in which we trace backwards from an individual program to the root node. The second is "`eccentricity`", in which we trace forwards from an individual program to the farthest leaf node.

`branch`. As discussed above, every program in our TE (aside from the root program of WSLG) is defined by a partition. The first step in rTE is to find the sequence of partitions, or the branch, that traces from the root node to a target program. To do this, one simply follows the immediate parent of the target program, and finds the parent of

the parent, etc., until reaching the root. Next, one can simply replace this chain of programs with their corresponding partition types:

$$\texttt{branch}(\texttt{prog i}) \equiv (\texttt{partition i}, \texttt{partition j}, \ldots, \texttt{root})$$

`eccentricity`. The second step in rTE is to compute the eccentricity by traversing from a target program to the farthest reachable leaf node. We do this by searching for the longest segment within a set of branches (where here, we define branches using program IDs, rather than partitions). For example, for program `42`:

1. Grab all branches that contain program `42`:

   [11, 22, 33, **42**, 56, 78]

   [11, 22, 33, **42**, 77, 99, 111]

   [11, 22, 33, **42**, 77, 88]

2. Get the segments towards the leaf node:

   `[56, 78]`

   `[77, 99, 111]`

   `[77, 88]`

3. Compute the length of the longest segment:

   `eccentricity(42) = 3`

Grouping programs with identical `(branch, eccentricity)`. Having defined a new label for each program, `(branch, eccentricity)`, we group all programs with the same label into a single compound node. The parent of each compound node is another compound node without the leaf partition, and with a strictly larger eccentricity. Finally, having assigned parents to all compound nodes, we have a reduced tree that is ready to be visualized. The result is shown in Figure S5, constructed with 5,837 nodes (reduced from the original 268,533 nodes).

9.6 Extracting the Good Program Network (GPN) from a Tree Embedding (TE)

From the tree embedding (TE) discussed earlier,we found that nearly all good programs are closely connected to form a single sub-tree. One can easily see that this is indeed the case in Figure S5, in which the full program space is visualized using the reduced tree embedding (rTE) described above. We use this finding to extract what we call the “Good Program Network”, or GPN, which consists of 4,230 programs whose performance exceeds that of WSLG, and 262 “connection programs” that are used to connect good programs into a single connected sub-tree. We include these connection programs in our analysis because they enable us to study how all 4,230 good programs are connected though different evolutionary lineages.

10 Extracting functional relationships within the good program space

In this section, we discuss all of the mathematics needed to perform a behavioral Tree Embedding (bTE) of the Good Program Network (GPN). We then use this embedding to define key and sloppy mutations, which can be used to understand the emergence of functional diversity within the GPN (see Section 9.6). Finally, we detail the algorithmic steps for Motif Decomposition (MD), and we discuss how it can be used to understand whether a group of programs can be locally or globally distinguished.

10.1 *confusion matrix, confusability,* and *distinguishability*

Both confusability and distinguishability measure how well a program can be identified among a group of other programs based on its behavioral sequences. Formally, it can be defined as shown in Table S14:

name	definition	note
confusion matrix	$C_{ij}(\{\mathcal{P}\}) \equiv p_{\{\mathcal{P}\}}\,(\text{prog } i \mid \text{prog } j)$	probability of mistaking program j for program i within an ensemble $\{\mathcal{P}\}$
confusability	$C_{ij}(\{\mathcal{P}\})\ \forall\, i \neq j$	off diagonal terms
distinguishability	$C_{ii}(\{\mathcal{P}\})$	diagonal terms

Table S14: Definitions of confusability and distinguishability.

where we define the confusion matrix as:

$$
\begin{aligned}
C_{ij}(\{\mathcal{P}\}) \equiv p_{\{\mathcal{P}\}}\,(\text{prog } i \mid \text{prog } j) &= \sum_{a_<, o_<} p\,(\text{prog } i \mid a_<, o_<)\ \underbrace{p\,(a_<, o_< \mid \text{prog } j)}_{ao\text{-distribution from prog } j} \\
&= \sum_{a_<, o_<} \frac{p\,(a_<, o_< \mid \text{prog } i)\ \cancel{p\,(\text{prog } i)}}{\sum_{i' \in \{\mathcal{P}\}} p\,(a_<, o_< \mid \text{prog } i')\ \cancel{p\,(\text{prog } i')}}\, p\,(a_<, o_< \mid \text{prog } j) \\
&= \sum_{a_<, o_<} \frac{p\,(a_<, o_< \mid \text{prog } i)\ p\,(a_<, o_< \mid \text{prog } j)}{\sum_{i' \in \{\mathcal{P}\}} p\,(a_<, o_< \mid \text{prog } i')}
\end{aligned}
\tag{S36}
$$

Here, we assume that all programs within a group are observed with the same probability, whereas all programs outside of the group are observed with zero probability:

$$
p_{\{\mathcal{P}\}}\,(\text{prog } i) = \begin{cases} 1/N & \text{if program } i \text{ is in an ensemble } \{\mathcal{P}\} \text{ consisting of } N \text{ programs} \\ 0 & \text{otherwise} \end{cases}
\tag{S37}
$$

10.2 *structural distance matrix* and *behavioral similarity matrix*

In our previous tree embedding and evolutionary algorithm, we only computed structural distances when checking if a candidate parent program is separated by a distance $d = 1$ from a child program (see definition of distance in Section 9.1). Here, we will perform the same computation but for all pairs of programs within GPN (this is because in our bTE algorithm, one must search for a parent amongst all programs that differ by a single mutation, which necessitates computing the full pairwise distance matrix).

To compute the behavioral similarity B_{ij}, we note that given a program i that generates behavioral sequences of fixed length l, the inverse distinguishability tells us how many programs j would be confused with program i; i.e., $n_{\text{confused}} \equiv \mathrm{round}\left(1/C^l_{ii}\right)$. We thus select the top n_{confused} programs, and assign them a value $B^l_{ij} = 1$. We repeat this procedure for sequences of length $l \leq 10$, and we sum the results to get a total measure of behavioral similarity:

$$
B_{ij} \equiv \sum_{l=1}^{10} B^l_{ij} \in [1, 10]
\tag{S38}
$$

This definition captures the intuition that some programs are sufficiently functionally similar that they cannot be differentiated even with long behavioral sequences, whereas other programs are sufficiently distinct that can easily be distinguished even with short sequences. Table S15 summarizes the definitions of structural distance and behavioral similarity:

name	definition	note
structural distance matrix	$d_{ij} \equiv d\,(\text{prog } i,\ \text{prog } j)$	
behavioral similarity matrix	$B_{ij} \equiv \sum_l B^l_{ij}$	B^l_{ij} is defined using fixed sequence length l

Table S15: Definition of structural distance and behavioral similarity matrices.

10.3 A behavioral Tree Embedding (bTE) of the Good Program Network (GPN)

One major goal of bTE is to ensure that the embedding is as functionally smooth as possible; that is, we want to maximize the number of neighbors (i, j) with behavioral similarity $B_{ij} = 10$. The steps of the bTE algorithm are as follows (see Figure S4B):

1. From the structural distance matrix d_{ij}, append a list of program pairs (prog i, prog j) with $d_{ij} = 1$ and $M_i \leqslant M_j$.
2. For each selected program pair, append the corresponding behavioral similarity B_{ij}.
3. For each program i in the selected pair, append the corresponding reward rate $\langle R \rangle$.
4. Sort the program pair list first by B_{ij}, and then by $\langle R \rangle$.
5. Beginning from the top of the list, check if program i is already in the final list; if not, append the row into the final list.

We use this embedding to define key and sloppy mutations:

name	definition
key mutation	prog $j \to$ prog i with $B_{ij} < l_{\text{max}}$
sloppy mutation	prog $j \to$ prog i with $B_{ij} = l_{\text{max}}$

Table S16: definition of key and sloppy mutations.

As can be seen in Figure S4B, bTE successfully finds a functionally smooth embedding in which most program pairs are functionally similar ($B_{ij} = 10$), with only 28 of 4,492 pairs having $B_{ij} < 10$. It is important to stress that the notion of key and sloppy mutations should not be taken as a binary classification, but rather as a continuum. In this study, we use this binary naming to provide a more intuitive discussion.

10.4 Functional labels of programs in the Good Program Network (GPN)

In the main text (Figure 3), we categorized individual programs within the GPN into 4 distinct classes based on the following two categorizations:

Functionally-Bayesian versus non-Bayesian programs. We define a functionally-Bayesian program to be one whose behavior cannot be distinguished from that produced by the ensemble of structurally-Bayesian programs (structurally-Bayesian programs are constructed by discretizing the Bayesian belief update; see Section 5.3 for details). To measure this, we use Equation (S36) to compute how easily a given program i in the GPN can be distinguished within the ensemble of 65 structurally-Bayesian programs in the GPN. We then select all programs whose distinguishability is less than a fixed threshold $\tilde{C}$. Using the results of the scatter plot in Figure 3B, we choose $\tilde{C} \approx 0.2$, for which there are two outliers (two discretized Bayesian programs whose distinguishability exceeds this threshold).

Globally distinguishable versus indistinguishable programs. We define globally-distinguishable programs as those whose behavior can be distinguished from other programs within the entire GPN. To measure this, we used Equation (S36) to compute how easily a given program i in the GPN can be distinguished from all other programs in the GPN. We then selected the top set of programs that could together account for half of the total distinguishability observed in the entire GPN. All remaining programs were classified as globally indistinguishable.

In Figure 3 in the main text, we further clustered all globally-distinguishable programs using `community_louvain`; their corresponding confusion matrix highlights the resulting 9 nearly-orthogonal program groups (inset of Figure 3D in the main text). Although this clustering gives a sense that the program space can be decomposed into discrete modules, it is important to stress (as discussed in Section 1.6) that the programs within each of these groups differ

in other properties. The clustering we did here is meant to provide an intuitive description of the functional diversity within the GPN.

10.5 Motif Decomposition (MD)

In Section 3, we discussed in depth how motif decomposition (MD) enables us to understand the functional diversity within the ensemble of good programs, and how it could provide a powerful way to explore a larger program space that might be demanded by a more complex task. Below, we detail the algorithmic steps for MD.

Step 1) Extract distinguishable sequences for a given program. We use distinguishable sequences to perform MD because these sequences best capture the behavioral features that distinguish a given program from all other programs, and they constitute a much more compact representation than the ensemble of all sequences that a program generates. We define the set of distinguishable sequences for program i analogously to how we defined a set of distinguishable programs, by selecting the most distinguishable sequences that together account for half of the total distinguishability of the program itself. The distinguishability of a sequence is defined analogously to the distinguishability of a program (see Equation (S36)):

$$C_{ii}^{\text{seq } k}(\{\mathcal{P}\}) = \frac{p\left(\text{seq } k \mid \text{prog } i\right)^2}{\sum_{i' \in \{\mathcal{P}\}} p\left(\text{seq } k \mid \text{prog } i'\right)} \tag{S39}$$

Step 2) Use distinguishable sequences to identify motifs (i.e., action-outcome loops) with length $l_{\textbf{motif}} \leqslant l_{\textbf{max}} = 10$ Given a set of distinguishable sequences, one can convert them into "win-lose-and-stay-go" sequences. For example:

$$\begin{array}{llllllllll}
a_- \, o_+ \, a_- \, o_- \, a_+ \, o_+ \, a_- & \to & a_- & o_+ & a_- & & & \to & W & \\
& & & & a_- & o_- & a_+ & \to & \quad l & \\
& & & & & & a_+ \quad o_+ \quad a_- & \to & \qquad w & \to Wlw
\end{array}$$

where L : lose-stay, W : win-stay, l : lose-go, and w : win-go. Next, we enumerate all possible candidate loops and their cyclic permutations, and we check if all concatenations of these loops produce sequences that are generated by the given program. For example:

$$\text{candidate loop: } Wlw \xrightarrow{\text{cyclic permutation}} \begin{array}{lll} Wlw & \to & WlwWlwWlw \\ lwW & \to & lwWlwWlwW \\ wWl & \to & wWlwWlwWl \end{array} \xrightarrow{\text{if all exist}} \text{append } Wlw \text{ to the motif list}$$

Step 3) Decompose all nonzero sequences in terms of extracted motifs In this final step, we use a soft decomposition approach that aims to find a set of motifs (or motif compound) to explain a given sequence. This decomposition prioritizes shorter motifs over longer ones while ensuring that we do not double-count the contribution of each motif. To illustrate this, consider decomposing the following sequence generated by program 24:

decompose $lLWllwlLl$
using motifs: $\{W,\ Lll,\ LWll,\ LlWl,\ Lllwl,\ Wlw,\ lw\}$

We first start by sorting these motifs based on length:

$$\{W,\ Lll,\ LWll,\ LlWl,\ Lllwl,\ Wlw,\ lw\}$$
$$\xrightarrow{\text{sort by length}} [[W],\ [lw],\ [Lll,\ Wlw],\ [LWll,\ LlWl],\ [Lllwl]]$$

For all motifs of a given length, we then check how much each motif explains the candidate sequence (not including parts of the sequence that have been attributed to shorter motifs):

sub-steps	part explained	amount explained
i) check W in $[W]$	$lL\underline{W}llwlLl$	1/9
ii) check lw in $[lw]$	$lLWl\underline{lwl}Ll$	3/9
iii) check Lll in $[Lll,\ Wlw]$	$lLWllwl\underline{Ll}$	2/9
check Wlw in $[Lll,\ Wlw]$	$lLWllwlLl$	0/9
v) check $LWll$ in $[LWll,\ LlWl]$	$\underline{lLWll}wlLl$	3/9

Table S17: Algorithmic steps of Motif Decomposition (MD). The underline indicates the candidate explanation provided by a given motif; gray letters indicate how much of the sequence has been explained by all motifs. Note that $LWll$ is committed without checking $LlWl$; this is because $LWll$ has explained all remaining parts of the sequence, so it is not possible for $LlWl$ to contribute any additional explanatory power.

The final result of the decomposition shown in Table S17 is thus:

$$lLWllwlLl \xrightarrow{\text{MD}} \left\{W : \frac{1}{9},\ lw : \frac{3}{9},\ Lll : \frac{2}{9},\ LWll : \frac{3}{9}\right\}$$

10.6 Locally distinguishable programs can be globally indistinguishable

In Figure 4 in the main text, we examined local clusters of descendants of programs 9 and 24, and we used this to gain insights into the role of sloppy and key mutations involved in preserving and altering behavior, respectively. There, we used a global perspective to define key and sloppy mutations (see Table S16). However, we can also use a local perspective to define key and sloppy mutations, which could be useful when examining local regions of the program space, or when adopting an evolutionary algorithm that iteratively grows the program space and thus does not have global information.

In Figure S19A-C, we use a local definition of distinguishability to show that many descendants of program 9 can be locally distinguished from one another, and could thus be separated by key mutations. Within these distinct programs, three stand out as the most distinguishable: program 24, 6592, and 8261. Interestingly, only program 24 remains distinguishable at a global level; the other two programs become indistinguishable from a large ensemble of functionally-Bayesian programs. In Figure S19D, we highlight two other functionally-Bayesian programs, 6118 and 5982, that generate overlapping behavioral sequences.

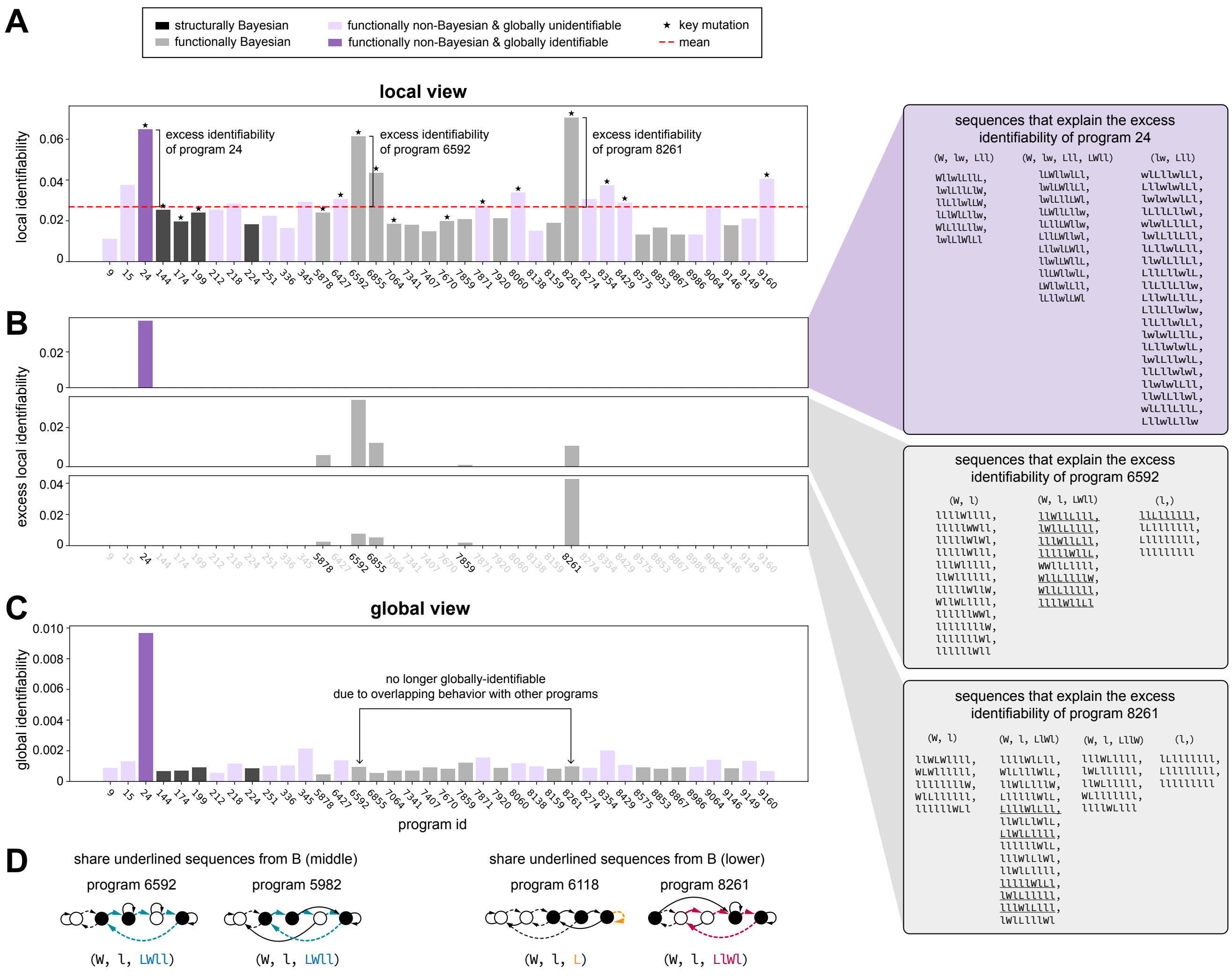

Figure S19: Local vs global notations of key mutation and sloppy mutation. (A) Many locally distinguishable programs emerge from local key mutations. Among those, three programs (program 24, 6592, and 8261) stand out as the most functionally distinct. Here, we compute local distinguishability with respect to the ensemble that includes program 9 and its descendants. **(B)** These three programs are the most distinguishable because they generate many unique sequences that other programs do not share. To illustrate this, we re-plot (A), keeping only those sequences that most contribute the distinguishability of the corresponding programs. E.g., for program 24 (purple), we isolate those sequences that contribute to the distinguishability of program 24 (purple box), and we look for all other programs that share those same sequences. The fact that program 24 is the only program that contributes to the purple histrogram indicates that these sequences can only be generated by program 24. In contrast, the sequences that contribute to the distinguishability of programs 6592 and 8261 are shared with few other programs (as illustrated by the multiple programs that contribute to the gray histrograms). **(C)** When we evaluate distinguishability within respect to all programs in the good program network, only program 24 remains distinguishable at a global level; programs 6592 and 8261 share sequences with other functionally-Bayesian programs. **(D)** Programs 5982 and 6118, which are located on distant regions of the program tree (see Figure 4A) are used as examples to illustrate that many programs in the good program network produce similar behavioral sequences. The sequences underlined in the middle panel of (B) are shared between programs 6592 and 5982; similarly those in the bottom panel of (B) are shared between programs 8261 and 6118. Note that these shared sequences can be realized by the same or a different combination of motifs (discussed in the main text).

11 All codes (ProgEnum)

In this section, we provide basic instructions for generating all of the results in this paper. We encourage readers to modify our code to fit their own needs, for example to develop different enumeration schemes, evolutionary algorithms, or more versatile tree embeddings. We look forward to hearing from you about how these ideas might be useful in different problem domains. For more detailed information and documentation, please visit our github repository `https://github.com/HermundstadLab/ProgEnum`.

11.1 Begin to explore

As a starting point, we provide three main datasets that were generated from sequentially running the main codes. Install `pandas` (we verified that this code runs with versions 1.4-2.1 of `pandas`), and use `example_code_to_begin.py` to load the following dataframes:

filename	codename	selected field	field description
df_enumP_para14_sorted	`df_enumP`	`program`	program tuple = (outmap, inmap)
		`progsize`	number of program state
		`eR`	reward rate
		`t_iter`	number of iteration before BDP converge
		`d2enumDB`	distance to discretized Bayesian
		`id_unique`	fixed id for each program (invariant of sorting)
df_pte_268533	`df_TE`	`target`	program id
		`source`	parent id
		`part`	partition type of merger program
		`eR`	reward rate
		`eR_source`	parent reward rate
		`num_outedge`	number of children
		...	...
df_gpn_pte	`df_bTE`	`pp_sim`	behavioral similarity
		`weight`	indicating key or sloppy mutation
		`pp`	distinguishability
		`iden`	log distinguishability
		`is_iden`	if program is distinguishable
		`branch`	list of program ids towards root
		`partbranch`	list of partition types towards root
		`d2root,`	distance to root
		`if_leaf`	if program is a leaf node
		`ecc`	eccentricity
		`tarP`	program tuple
		`souP`	parent program tuple
		`num_mut`	number of mutations
		`pp_DB`	distinguishability among all discretized Bayesians (DBs)
		`is_fn_DB`	if program is not distinguishable from all DBs
		`fn_comm`	program class: {-1:indistinguishable, -2:functional Bayesian, 0˜8: distinguishable}

Table S18: Selected datasets. Run `example_code_to_begin.py` to start exploring.

11.2 Full usage

Below we provide minimal instructions for running our code (visit our github repository for full documentation):

1. Run all `main_.py` sequentially to generate all necessary datasets.
2. Run `fig_.py` or `print_.py` separately to create plots or display data.

A minimal description of each file is listed in the tables below:

filename	algorithm	note	resource	run time
main_0_df_task.py		generate df_task	laptop	
main_1a_df_task_evalB.py	BDP_B	append reward rates of opt-B to df_task	laptop	
main_1b_df_task_genDB.py		append list of DBs to df_task	laptop	11m
main_1c_df_task_evalDB.py	BDP	append reward rates of DBs to df_task	cluster, 96 cpus	25m
main_2a_df_enumDB.py	DB, enumDB	generate df_enumDB_para14	laptop	12m
main_2b_df_enumDB_eval.py	BDP	append reward rates of enumDBs to df_enumDB_para14	cluster, 96 cpus	13m
main_3a_df_enumP.py	rule-out rules	generate df_enumP	laptop	43m
main_3b_df_enumP_eval.py	BDP	append reward rates of small programs to df_enumP_para14	cluster, 96 cpus	1.8h
main_3c_df_enumP_d2enumDB.py	structural distance	append minimal distance to DBs to df_enumP_para14	laptop	24m
main_3d_df_enumP_sorted_for_pte.py	TE	generate df_enumP_para14_sorted, df_enumP_para14_rand for TE and reward-randomized TE.	laptop	
main_3e_df_enumP_tsne.py	TSNE	generate df_enumP_para14_tsne	cluster, 96 cpus	2h
main_4a_pte.py	TE	generate df_pte_268533 for gephi visualization	cluster, 96 cpus	5h
main_4b_rpte.py	rTE	generate df_rpte_268533	laptop	50m
main_4c_rpte2gpn.py	GPN	generate df_gpn	laptop	
main_5_lenumP.py	evolutionary algorithm	generate df_lenumP5_toplog2	laptop	15m
main_6a_gpn_sdp.py	SDP	generate df_gpn_Y_lists	laptop	30m
main_6b_gpn_pprog.py	confusion matrix	generate df_gpn_pprog_arr	cluster, 96 cpus×45 jobs	10m
main_6c_gpn_simprog.py	behavioral similarity	generate df_gpn_simprog_arr	laptop	
main_6d_gpn_dprog.py	distance matrix	generate df_gpn_dprog_arr	cluster, 96 cpus×45 jobs	5m
main_6e_gpn_pte.py	TE	generate df_gpn_pte	laptop	
main_6f_gpn_fnDB.py	functional Bayesian	append distinguishability among DBs to df_gpn_pte	laptop	
main_6g_gpn_fnComm.py	functional labels	append functional labels to df_gpn_pte	laptop	5m
main_6h_df_gpn_local_keymut.py	local key mutation	generate df_gpn_local_keymut	laptop	
main_6i_gpn_seq2motif.py	MD	generate df_gpn_seq_motif_0, df_gpn_seq_motif_1, and df_gpn_seq_motif_2	cluster, 96 cpus	30m
main_7a_df_enumTaskB.py	enumerate hazard rate	generate df_enumTaskB_special_h	laptop	5m
main_7b_df_enumTaskDB.py	enumerate hazard rate; two types of DB	generate df_enumTaskDB_data	cluster, 96 cpus	3h
main_7c_df_enumTaskP.py	enumerate hazard rate; BDP on extended GPN	generate df_enumTaskP_ data	cluster, 96 cpus×68 jobs	5m
main_8a_te_alt.py	TE on alternative feature	generate df_pte_268536_alt, df_pte_268536_alt_branch	cluster, 128 cpus	6h
main_8b_rte_alt.ipynb	rTE on alternative feature	df_rpte_268536_alt	laptop	30m
main_8c_trees_er_vs_alt.ipynb		generate csv files for Gephi visualization	laptop	
main_8d_gpn_te_keymut.ipynb		extract key mutations for TE of GPN, and update df_gpn_te.csv	laptop	

Table S19: Main codes. These codes have to be run sequentially.

filename	figures, tables, boxes
fig_bar_keymut & iden_local vs global.py	Figure S19
fig_bar_local keymut.py	Figure S19
fig_bar_mut leading to fn sim vs dissim.py	Figure 3, main text
fig_bar_novel motifs inherited.py	Figure 4, main text
fig_fineDB.py	Figure S1
fig_imshow_fnComm.py	Figure 3, main text
fig_scatter_fnDB.py	Figure 3, main text
figS_boxplot_rTE.py	Figure S5
figS_imshow_why TE.py	Figure S12
figS_plot_why motif.py	Figure S16
figS_scatter_enumTaskB_DB.py	Figure S18
figS_scatter_enumTaskP_highDimSloppiness.py	Figure S15
figS_scatter_enumTaskP_seqVenn.py	Figure S18
figS_scatter_enumTaskP.py	Figure S18
figS_scatter_tsne.py	Figure S14
figS_table_lenumP.py	Figure 2, main text
figS_te vs bte.ipynb	Figure S10
figS_eR_thres_sweep.ipynb	Figure S7
figS_eR_vs_alt hist & count.ipynb	Figure S6
figS_nonB_motif.ipynb	Figure S11
print_critical h.py	Table S8
print_descendants of prog 9 and 24.py	Figure 4, main text; Figure S19

Table S20: Codes for figures and displaying data. These codes can be run independently.

filename	note
core_bdp.py	BDP for evaluating small program
core_bdpB.py	BDP for evaluating optimal Bayesian
core_enum.py	enumerating program
core_enumDB.py	discretizating and enumerating Bayesian
core_pte.py	all functions required for TE
core_sdp.py	SDP for small program
core_sdpB.py	SDP for optimal Bayesian

Table S21: Source codes.

REFERENCES AND NOTES

1. P. R. Montague, P. Dayan, C. Person, T. J. Sejnowski, Bee foraging in uncertain environments using predictive hebbian learning. *Nature* **377** 725–728 (1995).

2. M. Vergassola, E. Villermaux, B. I. Shraiman, ‘Infotaxis’ as a strategy for searching without gradients. *Nature* **445**, 406–409 (2007).

3. S. D. Boie, E. G. Connor, M. McHugh, K. I. Nagel, G. B. Ermentrout, J. P. Crimaldi, J. D. Victor, Information-theoretic analysis of realistic odor plumes: What cues are useful for determining location? *PLOS Comput. Biol.* **14**, e1006275 (2018).

4. E. Fujioka, I. Aihara, M. Sumiya, K. Aihara, S. Hiryu, Echolocating bats use future-target information for optimal foraging. *Proc. Natl. Acad. Sci. U.S.A.* **113**, 4848–4852 (2016).

5. S. B. M. Yoo, J. C. Tu, S. T. Piantadosi, B. Y. Hayden, The neural basis of predictive pursuit. *Nat. Neurosci.* **23**, 252–259 (2020).

6. M. M. Botvinick, Hierarchical models of behavior and prefrontal function. *Trends Cogn. Sci.* **12**, 201–208 (2008).

7. P. Shamash, S. F. Olesen, P. Iordanidou, D. Campagner, N. Banerjee, T. Branco, Mice learn multi-step routes by memorizing subgoal locations. *Nat. Neurosci.* **24**, 1270–1279 (2021).

8. A. Loisy, C. Eloy, Searching for a source without gradients: How good is infotaxis and how to beat it. *Proc R. Soc. A* **478**, 20220118 (2022).

9. J.-J. O. de Xivry, S. Coppe, G. Blohm, P. Lefevre, Kalman filtering naturally accounts for visually guided and predictive smooth pursuit dynamics. *J. Neurosci.* **33**, 17301–17313 (2013).

10. A. Solway, C. Diuk, N. Córdova, D. Yee, A. G. Barto, Y. Niv, M. M. Botvinick, Optimal behavioral hierarchy. *PLOS Comput. Biol.* **10**, e1003779 (2014).

11. F. Attneave, Some informational aspects of visual perception. *Psychol. Rev.* **61**, 183–193 (1954).

12. H. B. Barlow, “Possible principles underlying the transformation of sensory messages” in *Sensory Communication*, vol. 1, W. Rosenblith, Ed. (MIT, 1961), pp. 217–234.

13. E. P. Simoncelli, B. A. Olshausen, Natural image statistics and neural representation. *Annu. Rev. Neurosci.* **24**, 1193–1216 (2001).

14. E. Vul, N. Goodman, T. L. Griffiths, J. B. Tenenbaum, One and done? Optimal decisions from very few samples. *Cognit. Sci.* **38**, 599–637 (2014).

15. N. Tishby, D. Polani, “Information theory of decisions and actions” in *Perception-Action Cycle: Models, Architectures, and Hardware* (Springer, 2010), pp. 601–636.

16. G. Gigerenzer, W. Gaissmaier, Heuristic decision making. *Annu. Rev. Psychol.* **62**, 451–482 (2011).

17. B. Sauce, L. D. Matzel, The causes of variation in learning and behavior: Why individual differences matter. *Front. Psychol.* **4**, 395 (2013).

18. J. O’Doherty, M. L. Kringelbach, E. T. Rolls, J. Hornak, C. Andrews, Abstract reward and punishment representations in the human orbitofrontal cortex. *Nat. Neurosci.* **4** 95–102 (2001).

19. P. Vertechi, E. Lottem, D. Sarra, B. Godinho, I. Treves, T. Quendera, M. N. Oude Lohuis, Z. F. Mainen, Inference-based decisions in a hidden state foraging task: Differential contributions of prefrontal cortical areas. *Neuron* **106**, 166–176.e6 (2020).

20. L. P. Sugrue, G. S. Corrado, W. T. Newsome, Matching behavior and the representation of value in the parietal cortex. *Science* **304**, 1782–1787 (2004).

21. V. D. Costa, V. L. Tran, J. Turchi, B. B. Averbeck, Reversal learning and dopamine: A bayesian perspective. *J. Neurosci.* **35**, 2407–2416 (2015).

22. B. A. Bari, C. D. Grossman, E. E. Lubin, A. E. Rajagopalan, J. I. Cressy, J. Y. Cohen, Stable representations of decision variables for flexible behavior. *Neuron* **103**, 922–933.e7 (2019).

23. M. P. Karlsson, D. G. Tervo, A. Y. Karpova, Network resets in medial prefrontal cortex mark the onset of behavioral uncertainty. *Science* **338**, 135–139 (2012).

24. Y. Liu, Y. Xin, N.-l. Xu, A cortical circuit mechanism for structural knowledge-based flexible sensorimotor decision-making. *Neuron*, **109**, 2009–2024.e6 (2021).

25. C. C. Beron, S. Q. Neufeld, S. W. Linderman, B. L. Sabatini, Mice exhibit stochastic and efficient action switching during probabilistic decision making. *Proc. Natl. Acad. Sci. U.S.A.*, **119**, e2113961119 (2022).

26. A. Rajagopalan, R. Darshan, J. E. Fitzgerald, G. C. Turner, Expectation-based learning rules underlie dynamic foraging in *Drosophila*. *bioRxiv*, 2022.05.24.493252 (2022).

27. K. J. Åström, Optimal control of Markov processes with incomplete state information. *J. Math. Anal. Appl.* **10**, 174–205 (1965).

28. L. P. Kaelbling, M. L. Littman, A. R. Cassandra, Planning and acting in partially observable stochastic domains. *Artif Intell* **101**, 99–134 (1998).

29. R. S. Sutton, A. G. Barto, *Reinforcement Learning: An Introduction* (MIT Press, 2018).

30. R. L. Rivest, R. E. Schapire, Diversity-based inference of finite automata. *J. ACM* **41**, 555–589 (1994).

31. E. C. Tolman, Cognitive maps in rats and men. *Psychol. Rev.* **55**, 189–208 (1948).

32. T. E. Behrens, T. H. Muller, J. C. Whittington, S. Mark, A. B. Baram, K. L. Stachenfeld, Z. Kurth-Nelson, What is a cognitive map? organizing knowledge for flexible behavior. *Neuron* **100** 490–509 (2018).

33. W. F. Młynarski, A. M. Hermundstad, Adaptive coding for dynamic sensory inference," *eLife* **7** e32055 (2018).

34. M. Zacksenhouse, R. Bogacz, P. Holmes, Robust versus optimal strategies for two-alternative forced choice tasks. *J. Math. Psychol.* **54** 230–246 (2010).

35. F. Lieder, T. L. Griffiths, Resource-rational analysis: Understanding human cognition as the optimal use of limited computational resources. *Behav. Brain Sci.* **43**, e1 (2020).

36. J. Pearl, *Heuristics: Intelligent Search Strategies for Computer Problem Solving* (Addison-Wesley Longman Publishing Co. Inc., 1984).

37. G. Gigerenzer, R. Selten, *Bounded Rationality: The Adaptive Toolbox* (MIT Press, 2002).

38. E. Marder, A. L. Taylor, Multiple models to capture the variability in biological neurons and networks. *Nat. Neurosci.* **14**, 133–138 (2011).

39. T. Biswas, J. E. Fitzgerald, Geometric framework to predict structure from function in neural networks. *Phys. Rev. Res.* **4**, 023255, 2022.

40. K. J. Miller, M. M. Botvinick, C. D. Brody, From predictive models to cognitive models: Separable behavioral processes underlying reward learning in the rat. *bioRxiv*, 461129 (2021).

41. P. Krueger, F. Callaway, S. Gul, T. Griffiths, F. Lieder, Discovering rational heuristics for risky choice. PsyArXiv [Preprint] (2022). https://doi.org/10.31234/osf.io/mg7dn.

42. W. Młynarski, M. Hledík, T. R. Sokolowski, G. Tkačik, Statistical analysis and optimality of neural systems. *Neuron* **109**, 1227–1241.e5, (2021).

43. J. Najemnik, W. S. Geisler, Optimal eye movement strategies in visual search. *Nature* **434** 387–391 (2005).

44. A. J. Calhoun, S. H. Chalasani, T. O. Sharpee, Maximally informative foraging by caenorhabditis elegans. *eLife* **3**, e04220 (2014).

45. K. S. Brown, J. P. Sethna, Statistical mechanical approaches to models with many poorly known parameters. *Phys. Rev. E* **68**, 021904 (2003).

46. A. A. Prinz, D. Bucher, E. Marder, Similar network activity from disparate circuit parameters. *Nat. Neurosci.* **7**, 1345–1352 (2004).

47. B. C. Daniels, Y.-J. Chen, J. P. Sethna, R. N. Gutenkunst, C. R. Myers, Sloppiness, robustness, and evolvability in systems biology. *Curr. Opin. Biotechnol.* **19**, 389–395 (2008).

48. C. R. Gallistel, S. Fairhurst, P. Balsam, The learning curve: Implications of a quantitative analysis, *Proc. Natl. Acad. Sci. U.S.A.*, **101**, 13124–13131 (2004).

49. M. Rosenberg, T. Zhang, P. Perona, M. Meister, Mice in a labyrinth show rapid learning, sudden insight, and efficient exploration. *eLife* **10**, e66175 (2021).

50. G. R. Yang, M. R. Joglekar, H. F. Song, W. T. Newsome, X.-J. Wang, Task representations in neural networks trained to perform many cognitive tasks. *Nat. Neurosci.* **22**, 297–306 (2019).

51. S. Reed, K. Zolna, E. Parisotto, S. G. Colmenarejo, A. Novikov, G. Barth-maron, M. Giménez, Y. Sulsky, J. Kay, J. T. Springenberg, T. Eccles, J. Bruce, A. Razavi, A. Edwards, N. Heess, Y. Chen, R. Hadsell, O. Vinyals, M. Bordbar, N. de Freitas, A generalist agent. arXiv:2205.06175 [cs.AI] (2022).

52. QuantEcon, Quantecon: A high performance open source python code library for economics (2021); https://github.com/QuantEcon/QuantEcon.py.

53. M. Bastian, S. Heymann, M. Jacomy, "Gephi: An open source software for exploring and manipulating networks" in *Third International AAAI Conference on Weblogs and Social Media* (Association for the Advancement of Artificial Intelligence, 2009).

54. Y. Hu, Efficient, high-quality force-directed graph drawing. *Math. J.* **10**, 37–71 (2005).

55. T. Aynaud, python-louvain x.y: Louvain algorithm for community detection (2020); https://github.com/taynaud/python-louvain.

56. J. M. Whitacre, Biological robustness: Paradigms, mechanisms, and systems principles. *Front. Genet.* **3**, 67 (2012).

57. I. Wolfram Research, Mathematica, version 12.3 (2022); https://wolfram.com/mathematica.
